# Safety and Immunogenicity of a Replication-Defective Adenovirus Type 5 HIV Vaccine in Ad5-Seronegative Persons: A Randomized Clinical Trial (HVTN 054)

**DOI:** 10.1371/journal.pone.0013579

**Published:** 2010-10-27

**Authors:** Laurence Peiperl, Cecilia Morgan, Zoe Moodie, Hongli Li, Nina Russell, Barney S. Graham, Georgia D. Tomaras, Stephen C. De Rosa, M. Juliana McElrath, the NIAID HIV Vaccine Trials Network

**Affiliations:** 1 University of California San Francisco, San Francisco, California, United States of America; 2 Vaccine and Infectious Disease Division, Fred Hutchinson Cancer Research Center, Seattle, Washington, United States of America; 3 Bill & Melinda Gates Foundation, Seattle, Washington, United States of America; 4 Vaccine Research Center, National Institute of Allergy and Infectious Diseases, National Institutes of Health, Bethesda, Maryland, United States of America; 5 Duke University, Durham, North Carolina, United States of America; 6 University of Washington, Seattle, Washington, United States of America; The University of Chicago, United States of America

## Abstract

**Background:**

Individuals without prior immunity to a vaccine vector may be more sensitive to reactions following injection, but may also show optimal immune responses to vaccine antigens. To assess safety and maximal tolerated dose of an adenoviral vaccine vector in volunteers without prior immunity, we evaluated a recombinant replication-defective adenovirus type 5 (rAd5) vaccine expressing HIV-1 Gag, Pol, and multiclade Env proteins, VRC-HIVADV014-00-VP, in a randomized, double-blind, dose-escalation, multicenter trial (HVTN study 054) in HIV-1-seronegative participants without detectable neutralizing antibodies (nAb) to the vector. As secondary outcomes, we also assessed T-cell and antibody responses.

**Methodology/Principal Findings:**

Volunteers received one dose of vaccine at either 10^10^ or 10^11^ adenovector particle units, or placebo. T-cell responses were measured against pools of global potential T-cell epitope peptides. HIV-1 binding and neutralizing antibodies were assessed. Systemic reactogenicity was greater at the higher dose, but the vaccine was well tolerated at both doses. Although no HIV infections occurred, commercial diagnostic assays were positive in 87% of vaccinees one year after vaccination. More than 85% of vaccinees developed HIV-1-specific T-cell responses detected by IFN-γ ELISpot and ICS assays at day 28. T-cell responses were: CD8-biased; evenly distributed across the three HIV-1 antigens; not substantially increased at the higher dose; and detected at similar frequencies one year following injection. The vaccine induced binding antibodies against at least one HIV-1 Env antigen in all recipients.

**Conclusions/Significance:**

This vaccine appeared safe and was highly immunogenic following a single dose in human volunteers without prior nAb against the vector.

**Trial Registration:**

ClinicalTrials.gov NCT00119873

## Introduction

Approximately 2.7 million persons become infected with the human immunodeficiency virus (HIV-1) each year [1]. Although a recent clinical trial in Thailand found that a non-replicating canarypox vector vaccine combined with an envelope protein antigen may have provided limited protection [2], no vaccine has yet been shown to be highly effective in preventing HIV infection in humans. In non-human primate models, gene-based immunization with viral vectors, alone or in combination with DNA plasmid vaccines, have protected against infection or disease progression following challenge with immunodeficiency retroviruses; such protection is associated with the induction of immunodeficiency virus-specific T-lymphocyte responses [3], [4], [5], [6], [7], particularly CD8^+^ T-cell responses. Accordingly, HIV-1 vaccine development efforts in recent years have focused on recombinant viral vectors containing HIV-1 transgenes, alone or in combination with HIV-1 DNA plasmid vaccines or protein antigens.

The NIAID Vaccine Research Center's recombinant adenovirus 5 (rAd5) HIV-1 vaccine VRC-HIVADV014-00-VP was previously tested in a dose-escalation clinical trial that found local and systemic signs and symptoms increase in frequency and severity with increasing vaccine doses up to 10^11^ particle units (PU), but found no reactions of greater than moderate (grade 2) severity. Although that study did not stratify enrollment by prior Ad5 neutralizing antibody (nAb) titer, HIV-1-specific CD4^+^ and CD8^+^ T-cell responses tended to be of lower magnitude in the Ad5 nAb seropositive participants [8].

Although seroprevalence varies widely from one area to another, a substantial proportion of the world population has evidence of immunity to Ad5, including approximately 80% of adults in sub-Saharan Africa [9], [10], [11], [12]. We hypothesized that reactogenicity would be maximal and immunogenicity would be optimal in the absence of pre-existing nAb against the vector. Accordingly, to further investigate the safety and define the highest tolerable dose of this vaccine in Ad5 nAb-seronegative individuals, we performed a multicenter, randomized, double-blind, placebo-controlled clinical trial of a single intramuscular dose of VRC-HIVADV014-00-VP delivered at each of two escalating doses (10^10^ and 10^11^ PU) in participants with undetectable (<1∶12) titers of pre-existing Ad5 nAb. This study therefore evaluated the highest manufacturable dose of this rAd5 vector for safety and also characterized its immunogenicity by evaluating vaccine-induced T-cell responses against peptide pools reflecting diverse viral isolates, as well as HIV-1 binding and neutralizing antibody responses.

The primary (safety) objective was to characterize the safety and tolerability of a single dose of the adenoviral vector vaccine delivered at each of the two escalating doses in participants with low (<1∶12) titers of pre-existing Ad5 neutralizing antibodies.

The secondary immunogenicity objective was to evaluate the HIV-specific immunogenicity of a single dose of the adenoviral vector vaccine delivered at each of the two escalating doses, as assessed by IFN-γ ELISpot, Intracellular Cytokine Staining (ICS), HIV-1-binding antibodies, and neutralizing antibody assays.

## Methods

### Ethics Statement

The study protocol was approved by institutional review boards at each of the participating sites: Fred Hutchinson Cancer Research Center IRB for the Seattle, WA site; Vanderbilt University IRB for the Nashville, TN site; and University of California San Francisco Committee on Human Research for the San Francisco, CA site. All study participants provided written informed consent prior to participation. The protocol for this trial and supporting CONSORT checklist are available as supporting information; see Checklist S1 and Protocol S1. The trial is registered at ClinicalTrials.gov, registration NCT00119873.

### Participants

Forty-eight male and female study participants were enrolled by clinical staff at three HIV Vaccine Trials Network (HVTN) sites in the United States (Seattle, WA; San Francisco, CA; and Nashville, TN; 16 participants per site). A large pool of subjects was assessed for eligibility for multiple HVTN clinical trials, with candidates offered the opportunity to participate in a study for which they met inclusion criteria. For this study, those criteria included age 18-50 years, good general health, completion of a questionnaire assessing understanding of the study and the nature of participation, and being willing and able to provide informed consent. Laboratory inclusion criteria, tested within eight weeks prior to study enrollment, included negative HIV-1 serum antibody test; AST, ALT, alkaline phosphatase, total bilirubin, and creatinine within institutional upper limits of normal; negative blood tests for chronic hepatitis B and C; blood counts within normal range; and Ad5 nAb titer <1∶12. Pregnant women were excluded.

### Study Procedures

**Figure 1:**
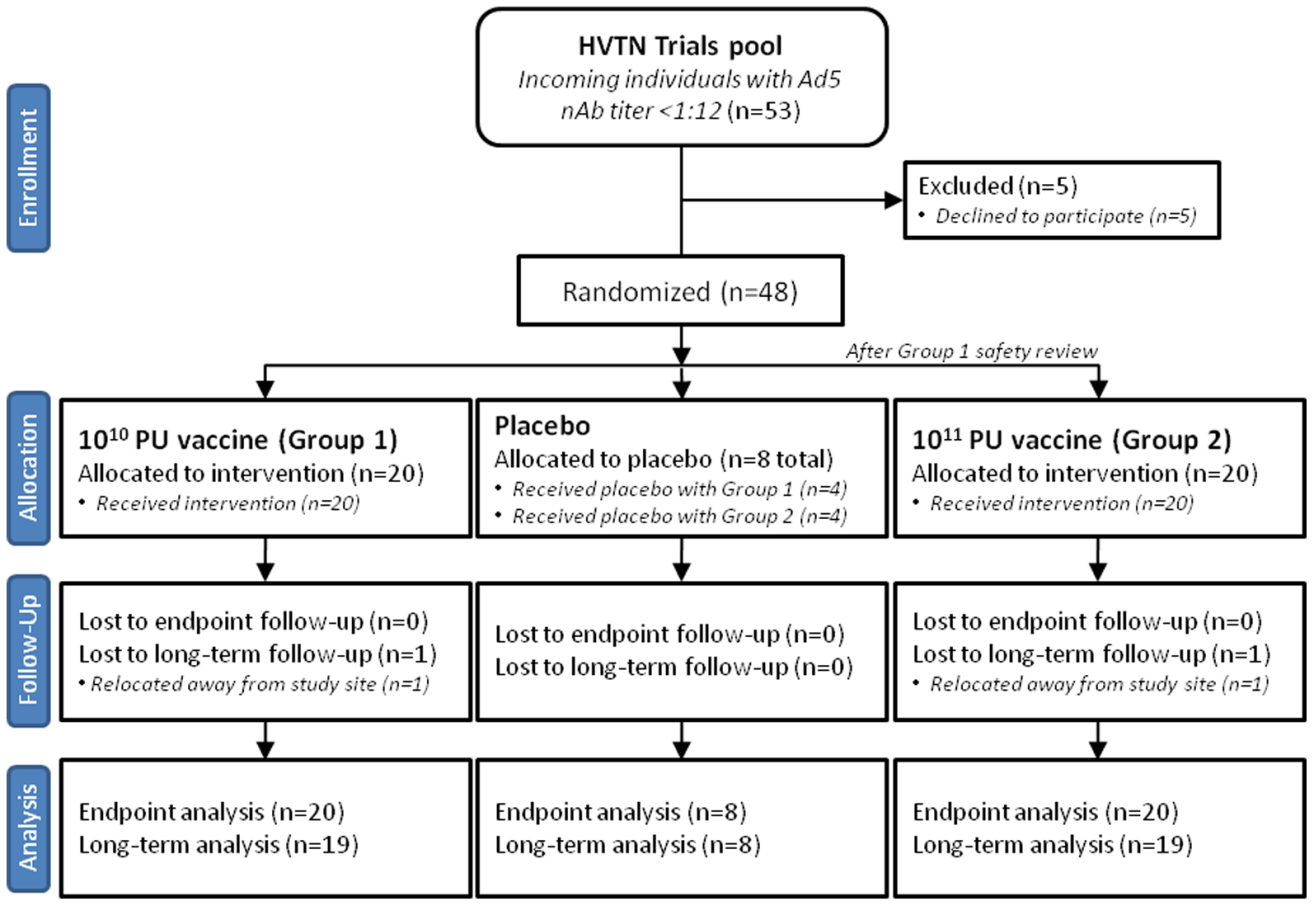
CONSORT statement 2010 flow diagram. Precise enrollment screening numbers are not available due to the candidate pool being screened for eligibility in multiple HVTN trials. Group 2 intervention proceeded only after a safety review of Group 1 interventions. For all analyses, both placebo groups were combined into one pool. Numbers shown in the analysis row are maximum available, with some specific assays using smaller numbers due to assay-specific losses, as detailed in the results.

All participants received a single injection of either adenovector vaccine VRC-HIVADV014-00-VP or buffer placebo, according to randomized treatment assignment, as a 1 ml intramuscular injection in the deltoid on the day of enrollment. The randomization sequence was obtained by computer-generated random numbers and provided to each site by a central data monitoring center. Randomization was done sequentially for each dose group and in blocks within the dose groups. The pharmacist at each site with responsibility for dispensing the appropriate vaccine was responsible for maintaining the security of the randomization code and did not participate in clinical assessment of participants. Participants in Group 1 received a 10^10^ PU dose of the study vaccine (n = 20) or placebo (n = 4). After a planned review of safety data on all Group 1 participants, enrollment was initiated in Group 2, in which participants received a 10^11^ PU dose of the study vaccine (n = 20) or placebo (n = 4). Figure 1 shows a CONSORT Statement flow chart of study enrollment, allocation and analysis [13].

Study clinicians and participants were blinded to the identity (vaccine versus placebo) of study injections. To avoid unblinding due to potential differences in appearance between vaccine and placebo products, the pharmacists placed a yellow overlay over each syringe before dispensing it to study clinicians. To avoid possible unblinding by vaccine-induced anti-HIV antibodies, participants were counseled throughout the trial not to obtain HIV testing outside of the research units, which provided HIV testing through a central laboratory using an algorithm that distinguishes between vaccine-induced seropositivity and actual HIV infection while preserving blinding of participants and site staff.

Participants were evaluated by a study clinician following vaccination, and each participant was instructed to record oral temperature and any symptoms in a standardized written log for at least three days; symptoms present on day 3 were followed to resolution. Injection-site symptoms (pain, tenderness, erythema, and induration) and systemic symptoms (malaise and/or fatigue, myalgia, headache, nausea, vomiting, chills, arthralgia, and temperature) were assessed.

Follow-up visits for clinical assessment, HIV risk reduction counseling, and monitoring of safety and immunogenicity took place at 14 days, 28 days, 3 months, 6 months, and 12 months following vaccination. As part of the study's safety evaluations, HIV antibody screening using commercially available diagnostic assay kits (Abbott HIVAB HIV 1/2 [rDNA], BioRad Genetic Systems HIV 1/2 Plus O EIA, and/or bioMerieux Vironostika HIV-1) as well as HIV RNA PCR assays were performed at the last study visit.

T-cell immunogenicity was assessed by HIV-1-specific interferon-gamma (IFN-γ) enzyme-linked immunosorbent spot (ELISpot) and intracellular cytokine staining (ICS) assays using cryopreserved PBMC obtained at the predetermined endpoint of 28 days post vaccination. IFN-γ ELISpot responses were also assayed at day 364. Assays for binding and neutralizing antibodies to HIV-1 were performed at day 28; binding antibody assays were also performed at day 0.

### Study Vaccine

VRC-HIVADV014-00-VP is a mixture of four replication-deficient, recombinant serotype 5 adenoviral vectors. Each vector expresses one of four HIV-1 antigens—clade B GagPol polyprotein, clade A Env, clade B Env, or clade C Env—and vectors are combined in a 3∶1∶1∶1 ratio, as previously described [8].

The four VRC-HIVADV014-00-VP recombinant adenoviral vectors were constructed by subcloning HIV-1 DNA sequences into an expression cassette in an E1-shuttle plasmid (AdFAST; GenVec, Gaithersburg, MD). The GV11 adenoviral backbone was chosen to reduce the risk of generating replication-competent adenovirus during clinical production. The GV11 backbone contains deletions of two essential regions, E1 and E4, as well as a partial E3 deletion, which renders the vaccine product replication deficient while providing increased transgene capacity [14]. The 293-ORF6 cell line used to propagate these vectors was developed by stably transforming HEK293 cells (of human embryonic kidney origin) with an inducible E4-ORF6 expression cassette, enabling the cells to efficiently complement the E1-, E4-, and partial E3-deleted adenoviral vector [15], [16].

The placebo consisted of the viral formulation buffer, composed of sodium chloride, Tris buffer, trehalose (low endotoxin), magnesium chloride, monooleate (Tween 80) and water for injection.

### Laboratory Methods

#### PBMC sample processing

Peripheral blood mononuclear cells (PBMC) were processed and cryopreserved from whole blood within 8 hours of venipuncture as previously described [17]. PBMC were thawed and rested overnight at 37°C/5% CO_2_ in R10 [RPMI 1640 (Gibco/Invitrogen, Grand Island, NY) containing 10% FCS (Gemini Bioproducts, West Sacramento, CA), 2 mM L-glutamine (Gibco/Invitrogen), 100 U/ml penicillin G, and 100 µg/ml streptomycin sulfate] prior to stimulation. A minimum cell viability of 66% following the overnight rest was required for assay by ELISpot and ICS.

Peptide stimulations. PBMC were assessed for *ex vivo* responses with pools of HIV-1 15-mer peptides covering global potential T-cell epitopes (PTE) representing HIV-1 peptides present in at least 15% of viral isolates in the Los Alamos database for Env, Gag, and Pol [18]. Peptides for a given gene product (Env, Gag, Pol) were pooled according to frequency, with pool one containing the most frequent peptides for a given protein. A total of eight pools (three for Env, two for Gag, and three for Pol), each containing no more than 160 peptides at a final concentration of 1 µg/ml per peptide, were used in this study. Response rates were measured for responses to all eight peptide pools. The magnitudes of responses were evaluated for pool 1 of Env, Gag and Pol, each containing the 160 peptides of highest sequence frequency for these proteins. For use as a positive non-HIV-1 antigen control, a pool of CMV 15-mer peptides overlapping by 11 amino acids spanning the entire p65 protein (kindly provided by the Division of AIDS, NIH) was also used.

For IFN-γ ELISpot assays, PBMC were cultured for 18–22 hours in 96-well plates with a PVDF membrane (Millipore MultiScreen-IP Filter Plate; Millipore, Billerica, MA) at a density of 200,000 cells in 125 µl R10 per well in the presence of the peptide pools. PBMC without added peptide served as the negative control, and phytohemagglutinin (PHA; Remel, Lenexa, KS) stimulation served as the positive control.

For ICS assays, cells were cultured for six hours in 96-well U-bottom plates at a density of one million cells per 200 µl well in the presence of anti-CD28 and anti-CD49d antibodies (each at 1 µg/ml; BD Biosciences, San Jose, CA), and Brefeldin A (10 µg/ml; BD Biosciences). PBMC with peptide diluent (1% DMSO) served as the negative control, and stimulation with 10 µg/ml staphylococcal enterotoxin B (SEB; Sigma-Aldrich, St. Louis, MO) served as a positive control.

#### IFN-γ ELISpot protocol

The IFN-γ ELISpot protocol has been described previously [19]. Before stimulation, the wells were coated with 100 µl of 10 µg/ml anti-human IFN-γ antibody (MabTech, Cincinnati, OH) in sterile phosphate-buffered saline (PBS; GibcoBRL) for 15–24 hours at 4°C. The plates were then washed with PBS, and PBMC were added and stimulated as described above. After stimulation, the wells were washed with wash buffer [PBS with 0.05% Tween 20 (Sigma-Aldrich)] and incubated with 100 µl of 1 µg/ml biotinylated anti-human IFN-γ (MabTech) in assay diluent [PBS with 0.5% bovine serum albumin (Sigma-Aldrich)] for 2.5 hours. The wells were washed with wash buffer and incubated with 100 µl of 1.33 µg/ml alkaline phosphatase-conjugated anti-biotin antibody (Vector Laboratories, Burlingame, CA) in assay diluent for two hours. The wells were then washed with wash buffer and incubated with 100 µl of BCIP/NBT (Pierce, Rockford, IL) for seven minutes, and then washed with deionized water to stop color development. After drying overnight, the wells were imaged and counted using a CTL Immunospot automated plate reader with Immunospot software (version 3.1; Cellular Technology Ltd., Cleveland, OH). Graphs were prepared using JMP software (SAS Institute, Cary, NC).

Data from a given participant were excluded if they met any of the following criteria: average response to PHA <400 spot-forming cells (SFC)/200,000 PBMC; average of negative control wells >20 SFC/200,000 PBMC; day 2 viability less than 66%; or blood obtained outside the allowable visit window. Data were also excluded if the ratio of the variance of the experimental wells for a specific peptide pool to [median+1] was ≥25.

#### ICS protocol

The ICS protocol has been described previously [20]. The eight-color staining panel included the following antibody-fluorophore conjugates: CD4 fluorescein, IL-2 phycoerythrin (PE), CD3 PE-Texas Red, CD8 PerCP-Cy5.5, IFN-γ PE-Cy7, IL-4 allophycocyanin (APC) and TNF-α Alexa-700 (all except CD3 PE-TR from BD Biosciences; CD3 PE-TR from Beckman-Coulter [Marseille, France]). The concentration of all antibodies was optimized by titration studies prior to use. The Cytokine Flow Cytometry (CFC) protocol from BD was used for fixation and permeabilization. Briefly, after stimulation, cells were stained with LIVE/DEAD Fixable Violet Dead Cell Stain (ViViD; Molecular Probes/Invitrogen, Eugene, OR), fixed and frozen at −80°C. Cells were thawed within three weeks of freezing, permeabilized and stained intracellularly. All samples were acquired on an LSR II flow cytometer capable of measuring 18 colors (BD Biosciences), collecting 100,000–300,000 PBMC. A High Throughput Sample Instrument (HTS; BD Biosciences) was used to collect samples from 96-well plates. All FACS analyses were performed using FlowJo software (version 6; Treestar, Ashland, OR).

#### HIV-1 binding antibody assays

Standardized research ELISAs were performed to delineate the antibody response to viral antigens encoded within the vaccine. End-point titers of antibodies were determined using 96-well Immulon2 (Dynex Technologies) plates coated with a preparation of purified recombinant HIV-1 proteins [21]. End-point titer was calculated as the most dilute serum concentration that gave an optical density reading of >0.2 above background.

Validated ELISAs were used to assess binding antibody responses to p24Gag (Quality Biologicals) and MN gp120 (Protein Sciences). Sera from cryopreserved samples were tested in duplicate using microtiter plates (NUNC) coated with antigen. Sera were diluted and incubated with the antigens bound to the plate. The plates were washed with an automated and calibrated plate washer (Bio-Tek) and read on an M2 plate reader (Molecular Devices). Positivity was scored by duplicate antigen-containing and non-antigen-containing wells (OD antigen – OD non-antigen) that had an optical density (OD) greater than or equal to an OD of 0.2 after subtracting background. Standard curves were generated from the plot of absorbance (450 nm) against the log of serum dilution, and sigmoidal curves were fit using a four-parameter logistic equation (Softmax Pro). The integrity of raw data acquisition and data analyses were electronically tracked (21CFR part 11 compliant) [22], [23].

Although the validated ELISA assays used gp120 Env proteins from laboratory-adapted HIV-1 strains, consensus gp140 envelope sequences show greater similarity to the vaccine strains. Therefore, after consensus gp140 envelope oligomers became available, we re-tested stored sera for binding antibodies against these antigens. Serum HIV-1 specific IgG responses (1/20 dilution) against ConS gp140 CFI and BCon.env03 gp140 CF (provided by Dr. L. Liao, Duke University) were measured by a standardized custom HIV-1 Luminex assay as previously described [23]. Antibody measurements were acquired on a Bio-Plex instrument (Bio-Rad), output as mean fluorescent intensity (MFI). The positive control in each assay was an HIV^+^ sera (HIV+16) standard curve (4PL fit) and the negative control was IVIG and blank beads. Samples were deemed positive if both the MFI and MFI minus blank were greater than 3-fold over the baseline (study visit 2) MFI and baseline MFI minus blank, respectively, and if the MFI minus blank was at least 732 MFI (based on the average + 3 standard deviations of 25 seronegative plasma samples).

#### HIV-1 neutralizing antibody assays

Neutralization was measured as a function of reduction in luciferase reporter gene expression after a single round of infection in TZM-bl cells as described [24], [25]. TZM-bl cells were obtained from the NIH AIDS Research and Reference Reagent Program, as contributed by John Kappes and Xiaoyun Wu. Briefly, 200 TCID_50_ of virus was incubated with serial 3-fold dilutions of test sample in duplicate in a total volume of 150 µl for one hour at 37°C in 96-well flat-bottom culture plates. Freshly trypsinized cells (10,000 cells in 100 µl of growth medium containing 75 µg/ml DEAE dextran) were added to each well. One set of control wells received cells and virus (virus control) and another set received cells only (background control). After incubation for 48 hours, 100 µl of cells was transferred to 96-well black solid plates (Costar) for measurement of luminescence using the Britelite Luminescence Reporter Gene Assay System (PerkinElmer Life Sciences). Neutralization titers are the dilution at which relative luminescence units (RLU) were reduced by 50% compared to virus control wells, after subtraction of background RLUs. Values below the limit of detection (1∶10 titer) were assigned a value of one-half the lowest dilution tested (i.e., a value of 5) when calculating geometric mean titer. Assay stocks of the molecularly cloned Env-pseudotyped viruses Bal.26, 92rw020.2 and 97ZA012.29 were prepared by transfection in 293T cells and were titrated in TZM-bl cells as described [25]. An assay stock of HIV-1 MN was produced in H9 cells and titrated in TZM-bl cells.

#### Adenovirus neutralizing antibody screening

Pre-vaccination sera were heat-inactivated and serially diluted in supplemented Dulbecco's modified Eagle's medium. Optimized concentrations of adenovirus type 5 or type 35 with a luciferase reporter gene was added, followed by A549 human lung carcinoma cells. The plates were then incubated at 37°C/10% CO_2_ for 24 hours. The culture medium was aspirated, and cells resuspended in Glo Lysis Buffer (Promega). The cell suspensions were transferred to a Black and White isoplate (Perkin Elmer) and Steady-Glo Luciferase Assay System Reagent (Promega) was added to the wells. The luminescence was measured on a luminometer. Neutralizing titer was defined as the highest titer that inhibited 90% luciferase activity of control wells tested without sera. Atypical control results invalidated an entire assay run. A titer of <1∶12 was considered negative [26].

### Statistical Methods

The sample size of 20 vaccine and four placebo recipients per group provided a 90% chance of observing at least one serious adverse experience if the true rate of such an event were at least 11%; there was a 90% chance that we would not observe at least one serious adverse experience if the true rate was no more than 0.5%. The precision to estimate immunogenicity was somewhat limited and therefore not a primary objective of the study. The width of a two-sided 95% confidence interval for each vaccine group was at most 0.44; for a 50% response rate, the 95% CI was approximately (0.28, 0.72).

Kruskal-Wallis tests were performed to compare severity of reactogenicity symptoms among the three groups. If the resulting p-value indicated a significant difference among the groups, further testing (Kruskal-Wallis) was then done to compare severity of reactogenicity symptoms in the two active treatment groups. To account for the multiple testing in these latter comparisons, the Simes procedure was used to control the false discovery rate at 0.05 [27]. The procedure was implemented separately for the local and systemic reactogenicity symptom comparisons.

To summarize the T-cell response data, positive response rates to any peptide pool and to each individual peptide pool were reported. Positivity of the individual IFN-γ response by ELISpot was determined by a one-sided bootstrap test of the null hypothesis that the responses in the experimental wells were twice those of the background (α = 0.05) [28]. A Westfall-Young approach [29] was used to adjust for the multiple comparisons across peptide pools. Peptide pools with adjusted one-sided p-values ≤0.05 were declared positive. In addition, the mean difference in the experimental and negative control wells had to exceed 50 SFC per 10^6^ PBMC for the response to be positive.

Positive responses were determined in the ICS assay by a one-sided Fisher's exact test applied to each response to a peptide pool vs. the response to the negative control with a discrete Bonferroni adjustment for the multiple comparisons. Peptide pools with adjusted p-values less than α = 0.00001 were considered positive. Positivity was determined for the combined cytokine subset: IFN-γ^+^ and/or IL-2^+^. If at least one cytokine subset was positive, the overall peptide pool was considered positive.

Confidence intervals for response rates were calculated with Agresti and Coull's method [30].

SAS (version 9.1; SAS Institute) and Splus (version 6.0; Insightful) were used for all analyses.

## Results

### Enrollment and Follow Up

Forty-eight participants consented, enrolled, and received their assigned injection at study entry between the months of April and September 2005, in two sequential groups to permit dose escalation. Data from placebo recipients in both groups were pooled in the analysis. As shown in Table 1, demographic characteristics were comparable among the treatment groups.

**Table 1:** Demographic characteristics of the study groups.

		Placebo (N = 8)	10^10^ PU (N = 20)	10^11^ PU (N = 20)	Total (N = 48)
**Sex**					
	Male	2 (25%)	14 (70%)	11 (55%)	27 (56%)
	Female	6 (75%)	6 (30%)	9 (45%)	21 (44%)
**Age in years**					
	Median	27.0	28.0	27.5	27.5
	Range	20–37	20–48	20–50	20–50
**Race/Ethnicity**					
	White*	5 (63%)	17 (85%)	15 (75%)	37 (77%)
	African-American*	1 (13%)	0	2 (10%)	3 (6%)
	Hispanic	0	1 (5%)	0	1 (2%)
	Native American/Alaskan	1 (13%)	0	0	1 (2%)
	Multiracial/Other	1 (13%)	2 (10%)	3 (15%)	6 (12%)
**Sexual orientation**					
	Heterosexual	6 (75%)	9(45%)	11 (55%)	26 (54%)
	Homo/bisexual	2 (26%)	11 (55%)	9 (45%)	22 (45%)

*Non-Hispanic.

The analysis was by intention to treat, modified to exclude individuals who entered in the randomization process but did not proceed with study enrollment; five such individuals were not included in the analysis. All 48 participants provided data for the reactogenicity assessments and blood samples for the primary immunogenicity analyses at day 28. Forty-six of the 48 participants (96%) completed planned follow up through one year post injection. The remaining two participants (one each in the 10^10^ PU and 10^11^ PU groups) relocated away from study sites prior to the one-year follow-up (Figure 1).

### Safety

No serious adverse events (such as permanent disability or incapacity, life-threatening medical conditions, or deaths) and no HIV infections occurred among study participants. Of 48 participants, 46 reported one or more adverse event as of the final study visit; the majority of these events were mild in severity and not related to study treatment. Treatment-associated events that were graded as severe by study staff occurred in four participants; all involved systemic reactogenicity consisting of two or more of the following: fever, chills, malaise, myalgia, fatigue, and/or headache. These symptoms occurred within 24 hours following injection of the 10^11^ PU dose and improved noticeably within 48 hours. The two additional adverse events rated severe (one viral syndrome occurring 201 days post vaccination, and one episode of low back pain, both in vaccine recipients) were reported as not related to study treatment.

**Figure 2:**
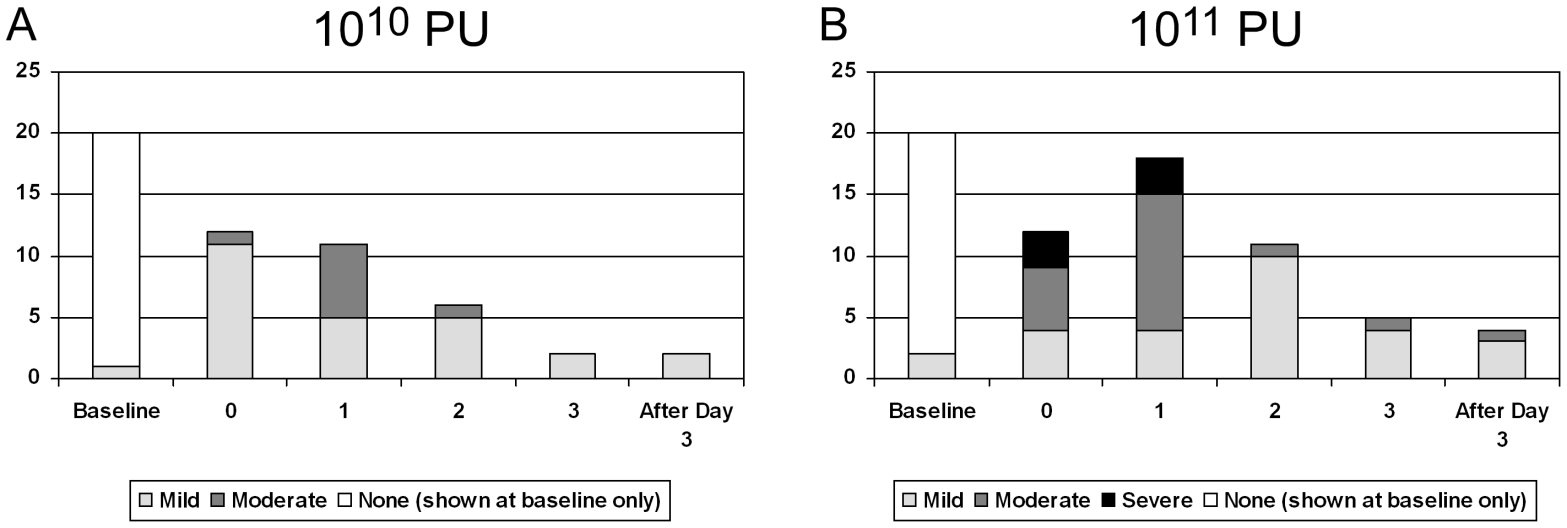
Duration and intensity of systemic reactogenicity. Number of vaccine recipients experiencing each level of severity in one or more systemic symptoms (malaise and/or fatigue, myalgia, headache, nausea, vomiting, chills, or arthralgia) is shown at baseline and for each day of the reactogenicity period. Panel A: 10^10^ PU dose; Panel B: 10^11^ PU dose. Only the most severe symptom level for each individual on each day is included.

Adverse events associated with vaccine treatment and beginning within 72 hours of injection are reported in Tables 2 (local reactions) and 3 (systemic reactions), which summarize the maximum severity during the reactogenicity period of a given symptom, sign, or specified combination in a given participant. Local reactogenicity occurred with greater severity in vaccine recipients than in placebo recipients (Table 2), but no significant difference was detected between the two vaccine dose levels. Seven of the nine systemic reactogenicity outcomes (malaise and/or fatigue, myalgia, headache, chills, arthralgia, maximum systemic symptom, and fever) occurred with greater severity in the higher dose (10^11^ PU) group than in the lower dose (10^10^ PU) group (Table 3), and were usually graded mild to moderate when they occurred. Figure 2 shows, for each dosing group, the intensity of all participants' maximum systemic reactogenicity symptom on each day of the reactogenicity period, indicating that these symptoms tended to peak by day 1 following injection, and that most resolved by day 3.

**Table 2:** Maximum local reactogenicity.

		Treatment – n (%)				Unadjusted p*
Symptom or sign**		Placebo (N = 8)	10^10^ PU (N = 20)	10^11^ PU (N = 20)	Total (N = 48)	(Kruskal-Wallis) 10^10^ vs. 10^11^PU
Pain						0.2726
	None	8 (100)	9 (45)	5 (25)	22 (45.8)	
	Mild	0	8 (40)	11 (55)	19 (39.6)	
	Moderate	0	3 (15)	4 (20)	7 (14.6)	
	Severe	0	0	0	0	
Tenderness						0.0967
	None	6 (75)	4 (20)	2 (10)	12 (25)	
	Mild	2 (25)	13 (65)	10 (50)	25 (52.1)	
	Moderate	0	3 (15)	8 (40)	11 (22.9)	
	Severe	0	0	0	0	
Pain and/or tenderness						0.1230
	None	6 (75)	4 (20)	0	10 (20.8)	
	Mild	2 (25)	11 (55)	12 (60)	25 (52.1)	
	Moderate	0	5 (25)	8 (40)	13 (27.1)	
	Severe	0	0	0	0	
Erythema						0.0796
	None	8 (100)	19 (95)	14 (70)	41 (85.4)	
	>0 to 10 cm^2^	0	1 (5)	5 (25)	6 (12.5)	
	>10 to 25 cm^2^	0	0	0	0	
	>25 cm^2^	0	0	1 (5)	1 (2.1)	
Induration						0.0335
	None	8 (100)	19 (95)	13 (65)	40 (88.3)	
	>0 to 10 cm^2^	0	1 (5)	5 (25)	6 (12.5)	
	>10 to 25 cm^2^	0	0	1 (5)	1 (2.1)	
	>25 cm^2^	0	0	1 (5)	1 (2.1)	
Erythema and/or induration						0.0435
	None	8 (100)	18 (90)	12 (60)	38 (79.2)	
	>0 to 10 cm^2^	0	2 (10)	6 (30)	8 (16.7)	
	>10 to 25 cm^2^	0	0	1 (5)	1 (2.1)	
	>25 cm^2^	0	0	1 (5)	1 (2.1)	

*For each local reactogenicity outcome, a statistically significant difference was found among the three treatment arms (Kruskal-Wallis p ≤0.05; values not shown). Unadjusted P values specifically for 10^10^ PU vs. 10^11^ PU arms (shown in table) were subsequently calculated. None of these are significant after applying Simes procedure to control the false discovery rate at 0.05 to account for the multiple comparisons.

**Mild: Pain/tenderness causing no or minimal limitation of use of limb; Moderate: pain/tenderness limiting use of limb or causing greater than minimal interference with usual social & functional activities; Severe: pain/tenderness causing inability to perform usual social & functional activities.

**Table 3:** Maximum systemic reactogenicity.

		Treatment – n (%)				Unadjusted p*
Symptom or sign**		Placebo (N = 8)	10^10^ PU (N = 20)	10^11^ PU (N = 20)	Total (N = 48)	(Kruskal-Wallis) 10^10^ vs. 10^11^PU
Malaise and/or fatigue						0.0245
	None	5 (62.5)	6 (30)	4 (20)	15 (31.3)	
	Mild	2 (25)	9 (45)	3 (15)	14 (29.2)	
	Moderate	1 (12.5)	5 (25)	9 (45)	15 (31.3)	
	Severe	0	0	4 (20)	4 (8.3)	
Myalgia						0.0039
	None	6 (75)	12 (60)	5 (25)	23 (47.9)	
	Mild	2 (25)	6 (30)	4 (20)	12 (25.0)	
	Moderate	0	2 (10)	9 (45)	11 (22.9)	
	Severe	0	0	2 (10)	2 (4.2)	
Headache						0.0073
	None	4 (50)	13 (65)	5 (25)	22 (45.8)	
	Mild	4 (50)	6 (30)	9 (45)	19 (39.6)	
	Moderate	0	1 (5)	5 (25)	6 (12.5)	
	Severe	0	0	1 (5)	1 (2.1)	
Nausea						NS
	None	7 (87.5)	17 (85)	13 (65)	37 (77.1)	
	Mild	1 (12.5)	3 (15)	4 (20)	8 (16.7)	
	Moderate	0	0	3 (15)	3 (6.3)	
	Severe	0	0	0	0	
Vomiting						NS
	None	8 (100)	20 (100)	9 (95)	47 (97.9)	
	Mild	0	0	1 (5)	1 (2.1)	
	Moderate	0	0	0	0	
	Severe	0	0	0	0	
Chills						<0.0001
	None	8 (100)	20 (100)	8 (40)	36 (75)	
	Mild	0	0	6 (30)	6 (12.5)	
	Moderate	0	0	3 (15)	3 (6.3)	
	Severe	0	0	3 (15)	3 (6.3)	
Arthralgia						0.0136
	None	7 (87.5)	18 (90)	10 (50)	35 (72.9)	
	Mild	1 (12.5)	1 (5)	9 (45)	11 (22.9)	
	Moderate	0	1 (5)	1 (5)	2 (4.2)	
	Severe	0	0	0	0	
Maximum systemic symptoms						0.0027
	None	2 (25)	6 (30)	2 (10)	10 (20.8)	
	Mild	5 (62.5)	8 (40)	3 (15)	16 (33.3)	
	Moderate	1 (12.5)	6 (30)	11 (55)	18 (37.5)	
	Severe	0	0	4 (20)	4 (8.3)	
Temperature (°C)						0.0052
	34.0–37.6	8 (100)	19 (95)	11 (55)	38 (79.2)	
	37.7–38.6	0	1 (5)	5 (25)	6 (12.5)	
	38.7–39.3	0	0	3 (15)	3 (6.3)	
	39.4–40.5	0	0	1 (5)	1 (2.1)	
	40.6 or greater	0	0	0	0	

*For each systemic reactogenicity outcome except nausea and vomiting (NS: not significant), a statistically significant difference was found among the three treatment arms (Kruskal-Wallis p ≤0.01; values not shown). For those outcomes showing an overall significant difference, unadjusted p values specifically for 10^10^ PU vs. 10^11^ PU arms (shown in table) were subsequently calculated. All of these are significant after applying Simes procedure to control the false discovery rate at 0.05 to account for the multiple comparisons.

**Severity definitions are given in the *HVTN Table for Grading Severity of Adverse Experiences (Revised Sep 2002)*; see also *Division of AIDS Table for Grading the Severity of Adult and Pediatric Adverse Events (December, 2004)*. In general, mild systemic reactions are those causing no or minimal interference with usual social and functional activities; moderate reactions cause greater than minimal interference with such activities; and severe reactions cause inability to perform such activities.

Clinical laboratory monitoring of blood samples revealed no significant changes in safety parameters following vaccination. All laboratory abnormalities were graded as mild except three elevations in creatine phosphokinase graded as moderate, all of which were reported as either not related or probably not related to study treatment. To screen for the theoretical possibility of recombination between vaccine product and circulating adenoviruses, swab cultures were performed on participants reporting symptoms of upper respiratory infection or conjunctivitis within four weeks following study injection. Of two conjunctival and 18 throat cultures taken, all were negative for adenovirus.

When participants completed study visits, they were tested with commercially available HIV antibody tests to inform them about the likelihood of testing positive for antibodies if tested outside of the study site. Of those with commercial HIV antibody testing results available at 12 months following injection, 18/19 participants who received the 10^10^ PU dose, 15/19 who received the 10^11^ PU dose, and 0/8 placebo recipients tested positive for HIV antibodies by at least one of three commercially available enzyme immunoassays. The majority (87% of vaccine recipients) had vaccine-induced antibody responses detected by the Abbott HIVAB HIV 1/2 diagnostic assay. Lower antibody response rates were seen with the BioRad Genetic Systems HIV 1/2 Plus O EIA (11%) and bioMerieux Vironostika HIV-1 (8%) diagnostic assays.

### T-Cell Immunogenicity

**Figure 3:**
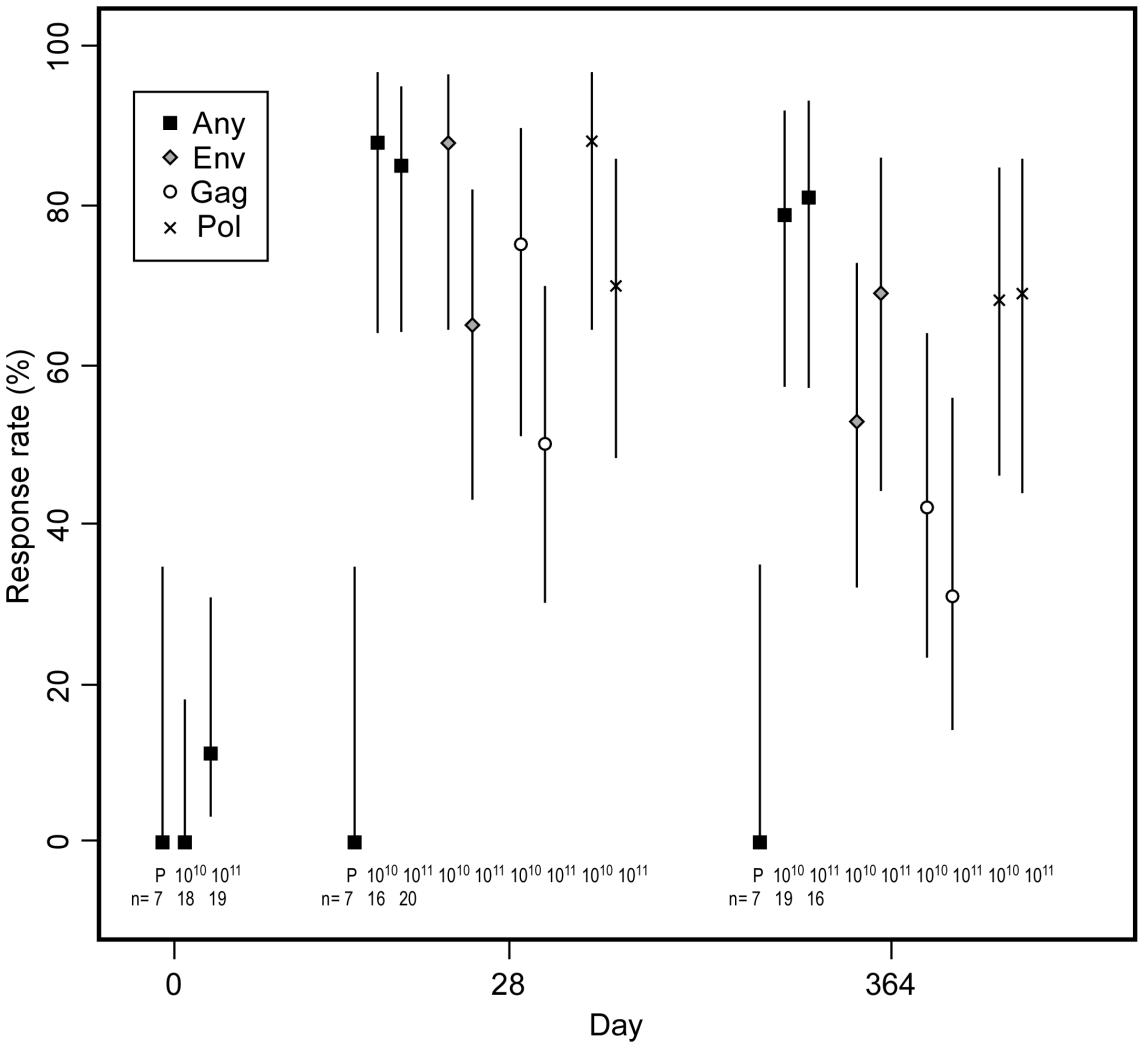
T-cell responses to HIV antigens by IFN-γ ELISpot. Points indicate percentage of participants with positive responses; n denotes the number in each group with ELISpot data. Lines indicate 95% confidence intervals.

In IFN-γ ELISpot assays at day 28 post vaccination, 14/16 (88%) of participants who received the 10^10^ PU dose, 17/20 (85%) of those who received the 10^11^ PU dose, and 0/7 placebo recipients mounted HIV-1-specific T cell responses to at least one antigen pool (Figure 3). Data at day 28 from five participants (one in the placebo group and four in the 10^10^ PU group) could not be analyzed because of poor cell viability or high assay background levels. Of the 36 vaccine recipients with ELISpot data, 64% had PBMC that recognized more than one of the vaccine antigens (Gag, Pol, or Env) and 47% recognized all three antigens at day 28. Two of 19 (11%) participants in the 10^11^ PU group also had positive IFN-γ ELISpot responses at day 0, but both individuals showed responses to a larger number of antigens at day 28 (data not shown). The proportion of vaccine recipients responding to a given antigen pool at day 28 ranged between 50% and 88% of assayable participants, depending on pool and vaccine dose. Persistent T-cell responses from PBMC collected at day 364 following vaccination were observed at both vaccine doses and remained distributed across all three HIV-1 antigens (Figure 3).

**Figure 4:**
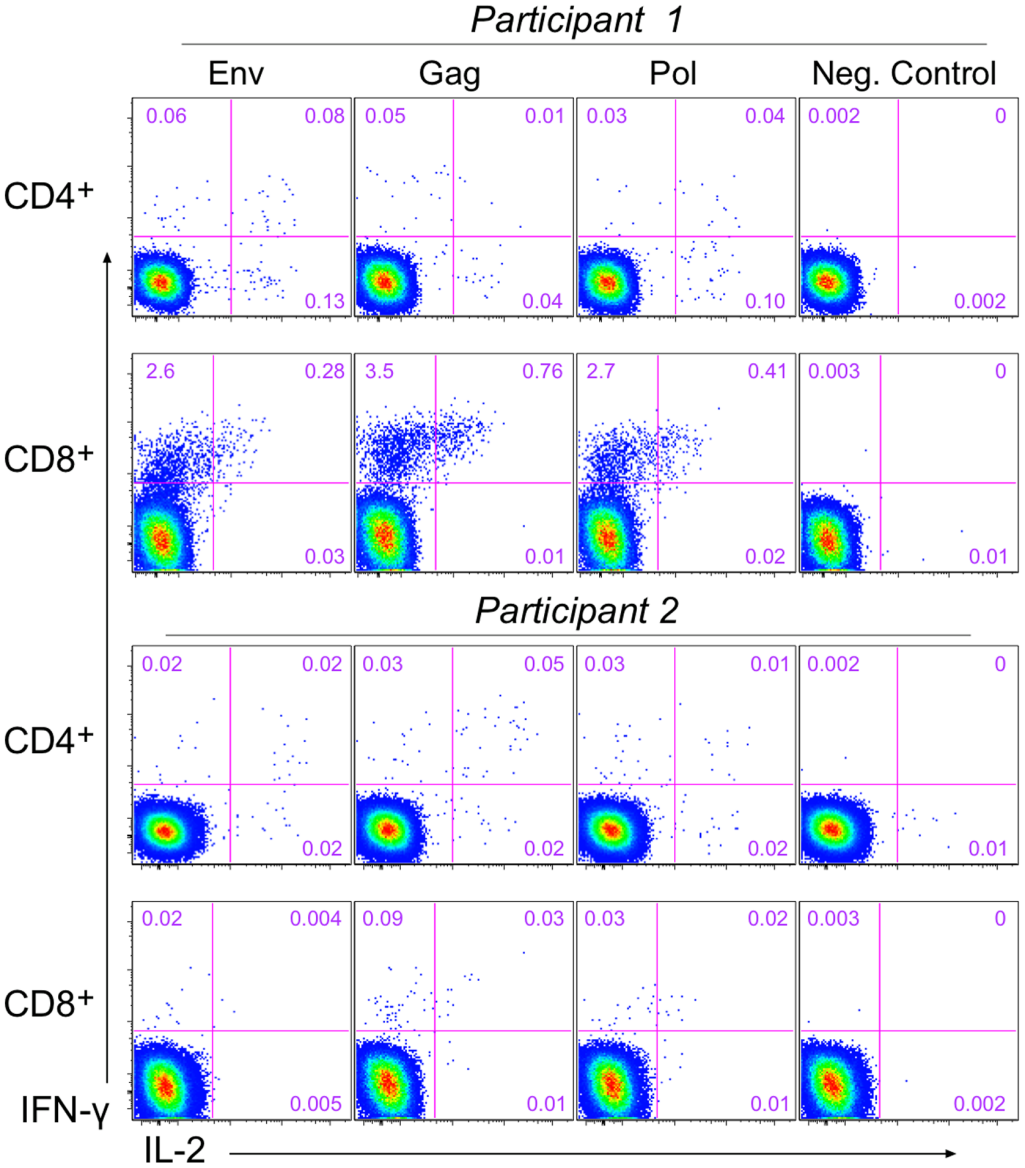
Staining profiles for ICS analysis for two trial participants. The expression of IFN-γ and IL-2 are shown for CD4^+^ and CD8^+^ T cells in response to stimulation with the first pools for Env, Gag, and Pol and for the negative control (peptide diluent, 1% DMSO) at day 28. Numbers on the plots are the percentages of CD4^+^ or CD8^+^ T cells producing the cytokine or combination of cytokines. Participant 1 represents an example of a high CD8^+^ T-cell response and Participant 2 represents low CD4^+^ and CD8^+^ T-cell responses. All responses are positive as tested for cells producing IFN-γ and/or IL-2 except for Env and Pol for participant 2. Both participants were in the 10^11^ PU dose group.

**Figure 5:**
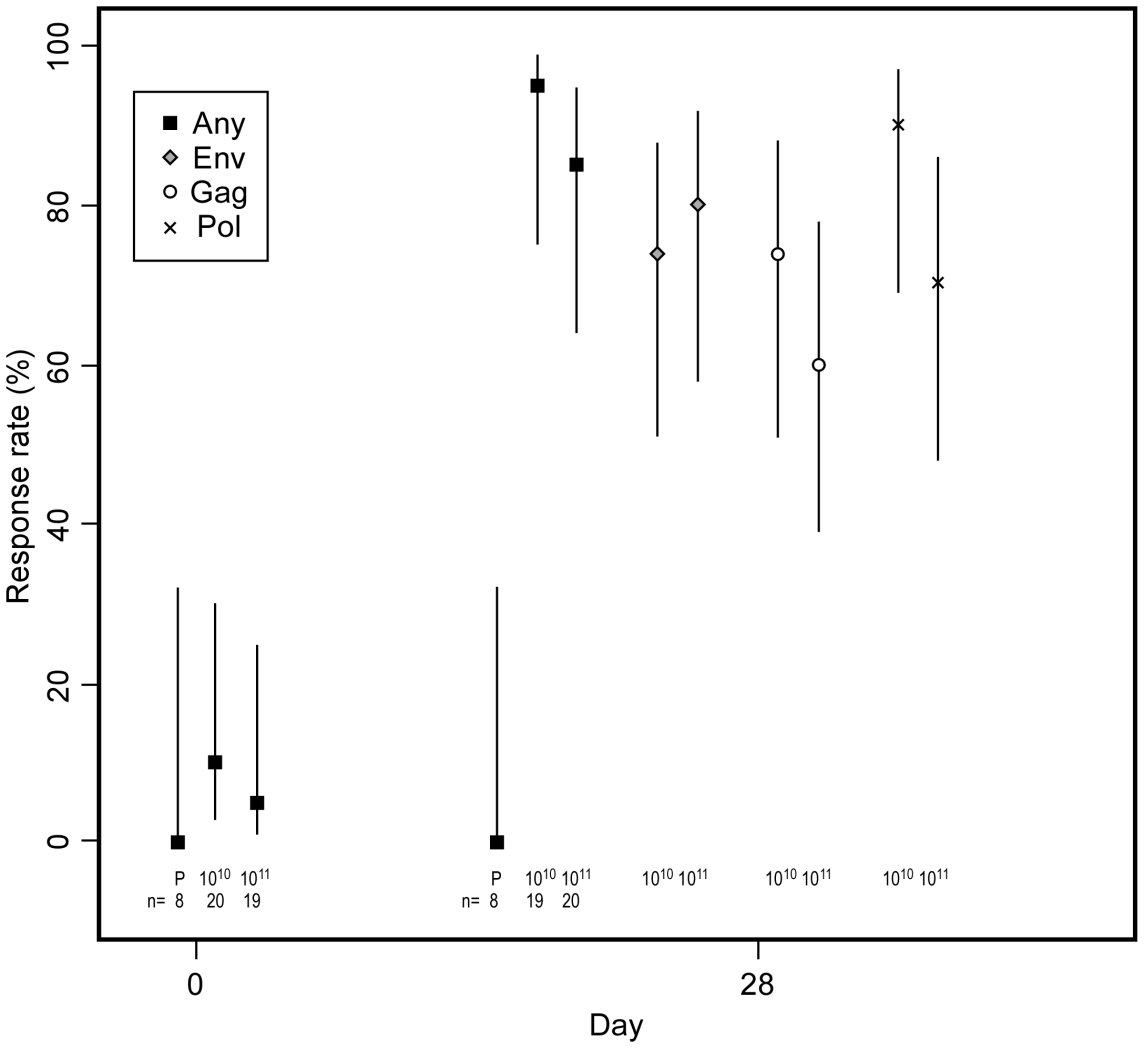
T-cell responses to HIV antigens by ICS. Points indicate percentage of participants with positive responses (for CD4^+^ or CD8^+^ T cell subsets, and for IFN-γ or IL-2 detection); n denotes the number in each group with ICS data. Lines indicate 95% confidence intervals.

By ICS analysis, both CD4^+^ and CD8^+^ T cell responses were detectable (representative high and low positive samples shown in Figure 4). HIV-specific CD4^+^ T cells were distributed among cells co-producing IFN-γ and IL-2, and cells producing IFN-γ without IL-2 or IL-2 without IFN-γ. HIV-specific CD8^+^ T cells largely produced IFN-γ without IL-2, although some cells co-producing both cytokines were detected. Examining the overall CD4^+^ or CD8^+^ T-cell response, ICS analyses detected vaccine responses in 60–90% of vaccine recipients according to antigen pool (Figure 5). The proportion of participants with CD8^+^ T-cell responses was comparable, but nominally higher, than those with CD4^+^ T-cell responses. At day 28, CD8^+^ T-cell responses (to any peptide pool, and for IFN-γ or IL-2 detection) were found in 17/18 (94%; 95% CI: 74–99%) of participants in the 10^10^ PU dose group with CD8^+^ ICS data and in 17/20 (85%; 64–95%) of those in the 10^11^ PU dose group; CD4^+^ T-cell responses were found in 16/19 (84%; 62-95%) of participants in the 10^10^ PU dose group with CD4^+^ ICS data and in 11/20 (55%; 34–74%) of those in the 10^11^ PU dose group. Two vaccinees in the 10^10^ PU dose group were excluded from the CD8^+^ analysis, one due to high background and one due to low numbers of T cells (this participant was also excluded from the CD4^+^ analysis due to low numbers of T cells). At day 28, T-cell responses in both CD4^+^ and CD8^+^ subsets were detected in 26 of 38 (68%) vaccine recipients with specimens for both subsets. Of these 26, two vaccine recipients had positive ICS responses in both T cell subsets at day 0, but both individuals showed responses to a larger number of antigens at day 28 (data not shown).

The frequency and specificity of T-cell responses, assessed by ELISpot and ICS assays, appeared similar at both vaccine doses, but the study was not sufficiently powered to formally compare dose groups.

**Figure 6:**
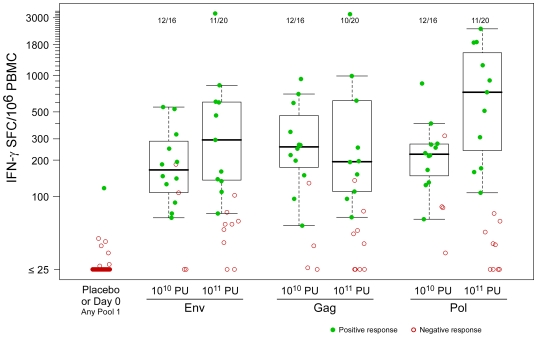
Magnitudes of T-cell responses to pool 1 global PTE peptides, measured by IFN-γ ELISpot assay. Day 0 (left column) is a combined analysis including responses from PBMC from placebo recipients; day 28 (remaining columns) is sorted by HIV-1 gene product. The box plots are based only on the positive responses. The box indicates the median and interquartile range (IQR); whiskers extend to the furthest point within 1.5 times the IQR from the upper or lower quartile. The number of vaccinees with a positive response out of the total number of vaccinees tested is indicated above the bars.

**Figure 7:**
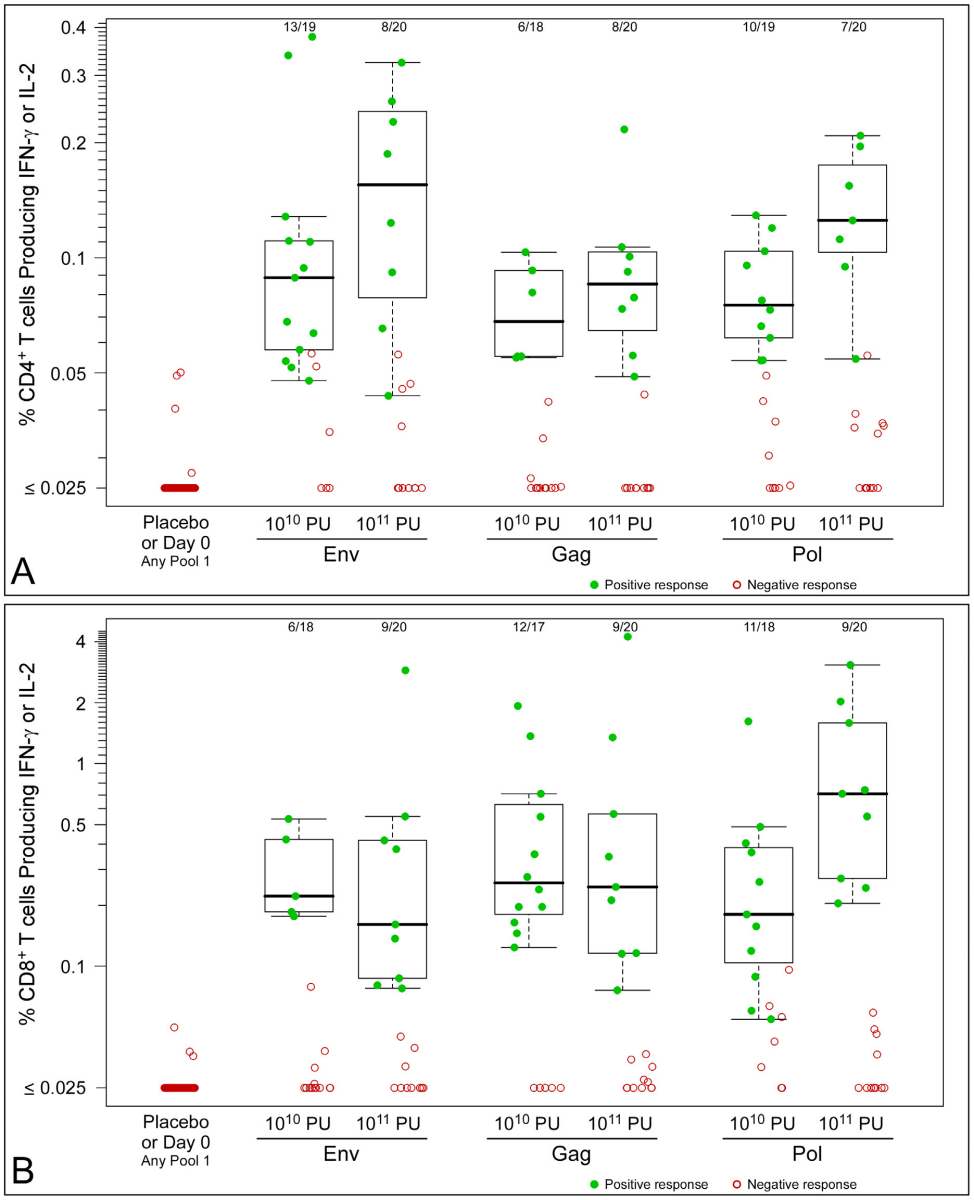
Magnitudes of responses by ICS to pool 1 peptides at day 28 following study injection. A) CD4^+^ T cell responses. B) CD8^+^ T cell responses. The box plots are based only on the positive responses. The box indicates the median and interquartile range (IQR); whiskers extend to the furthest point within 1.5 times the IQR from the upper or lower quartile. The number of vaccinees with a positive response out of the total number of vaccinees tested is indicated above the bars.

HIV-1 antigen-specific response magnitudes at day 28 for the IFN-γ ELISpot assay are shown in Figure 6, and for ICS assays in Figure 7A (CD4^+^ T-cell responses) and 7B (CD8^+^ T-cell responses). In the IFN-γ ELISpot assay, median response magnitudes ranged between approximately 150 and 800 SFC/10^6^ PBMC according to antigen pool. In the ICS analyses, median percentages of T cells showing IFN-γ or IL-2 responses at day 28 to each of the three antigens in each of the two vaccine dose groups ranged from approximately 0.05% to 0.15% (CD4^+^ T-cell responses) and from approximately 0.1% to 0.7% (CD8^+^ T-cell responses) according to antigen (Figures 7A and 7B).

Of the 39 participants with detectable HIV-1-specific CD4^+^ T-cell responses to any PTE pool by ICS on day 28, 18 (46%) had responses detected in the IFN-γ^+^IL-2^+^ subset, 9 (23%) had IL-2^+^IFN-γ^−^ responses, and 9 (23%) had IFN-γ^+^IL-2^−^ responses. Of the 38 participants with HIV-1-specific CD8^+^ T-cell responses, 21 (55%) had responses in the IFN-γ^+^IL-2^+^ subset, none had IL-2^+^IFN-γ^−^ responses, and 32 (84%) had responses in the IFN-γ^+^IL-2^−^ subset (data not shown).

### Antibody Immunogenicity

Sera from days 0 and 28 were evaluated by multiple assays for HIV-1-specific antibodies. Using an endpoint dilution ELISA, sera from 0/8 placebo recipients were positive for antibodies against any tested vaccine-encoded HIV-1 antigen, and 19/20 (95%) in each of the 10^10^ PU and 10^11^ PU dose groups were positive to one or more vaccine-encoded HIV-1 antigen (clade A, B, or C Env; clade B Gag, Pol, or Nef). Antibody response rates to Env antigens were most frequent, ranging from 75% to 95% of participants across clades and doses, with geometric mean titer (GMT) ranging between 102 and 187 among dose or clade groups. Responses to Gag (20% and 45% of participants in the 10^10^ PU and 10^11^ PU groups, respectively, with GMT 90 in both dose groups) and Pol (15% and 5%, with GMTs 30 and 90 respectively) appeared to be less frequent. Antibody responses to Nef, which was not included in the vaccine, were minimal (15% [GMT 30] in the 10^10^ PU group and 0% in the 10^11^ PU group).

Another ELISA for binding antibodies against laboratory-adapted HIV-1 gp120 Env and p24 Gag proteins showed sera from 0/8 placebo recipients, 0/20 participants in the 10^10^ PU dose group and 2/20 (10%) in the 10^11^ PU dose had an elevation in anti-Gag antibodies at day 28 after vaccination, while no participants were positive for antibodies against the gp120 antigen. Using a more sensitive flow cytometry-based multiplex assay and consensus envelope antigens, sera from 0/7 placebo recipients, 14/20 (70%) participants in the 10^10^ PU dose group and 15/20 (75%) in the 10^11^ PU dose group were positive for antibodies against Bcon.env03 gp140 CF; 0/7 placebo recipients and 20/20 vaccine recipients at both dose levels were positive for antibodies against ConS gp140 CFI. Among positive responders, geometric mean fluorescence for Bcon.env03 gp140 CF was 5008 (3925 in the 10^10^ PU dose group and 6286 in the 10^11^ PU dose group) and geometric mean fluorescence for ConS gp140 CFI was 9074 (7966 in the 10^10^ PU dose group and 10336 in the 10^11^ PU dose group). Functional activity of the vaccine-induced antibodies was assessed in a neutralizing assay. Neutralizing activity against HIV-1 strain MN was minimal. At day 28, 2/8 placebo recipients (25%, GMT  = 12), 13/20 from the 10^10^ PU dose group (65.0%, GMT  = 23), and 1/20 from the 10^11^ PU group (5.0%, GMT  = 6) had detectable responses.

## Discussion

In this randomized, double-blind, placebo-controlled clinical trial, the multiclade, multiprotein rAd5 HIV-1 vaccine VRC-HIVADV014-00-VP appeared safe and was highly immunogenic among participants seronegative for prior nAb to Ad5. This study extends the prospective safety testing of recombinant type 5 adenoviral vectors specifically to include Ad5-seronegative volunteers, and provides evidence that in this group the 10^10^ PU dose is of comparable immunogenicity to, and better tolerated than, the 10^11^ PU dose.

A single dose of the vaccine induced T-cell responses against all three HIV-1 proteins encoded by the vector. Responses to one or more antigen pools were detected in 85% to 95% of vaccine recipients. Among responders, median ELISpot response magnitudes were on the order of several hundred SFC/10^6^ PBMC, and ICS detected CD4^+^ and CD8^+^ T-cell responses on the order of 0.1% to 1% of T cells. Sixty-five percent of vaccinees showed responses in both CD4^+^ and CD8^+^ T cell subsets at 28 days post vaccination. Responses in individual participants reached magnitudes of up to 3000 SFC/10^6^ PBMC in the IFN-γ ELISpot assay and up to 4% of total CD8^+^ T cells in the ICS assay. The majority of vaccinees (∼80%) had IFN-γ-secreting, HIV-1-specific T cells persisting at one year post vaccination. Responses to Pol were observed at higher frequencies than reported in previous in trials that used a DNA-prime/rAd5 boost regimen, as discussed below.

All vaccinees (and no placebo recipients) developed binding antibodies against a consensus HIV-1 gp140 oligomer at day 28. The vaccine also induced binding antibodies against vaccine antigens (but not against gp120 antigens from laboratory-adapted HIV-1 strains) in the majority of recipients, and induced neutralizing antibodies at low titers in approximately half of participants in the lower dose group.

Given the diversity of circulating HIV strains and the rapidity with which variants arise within infected individuals, a vaccine's ability to induce a broad response across viral proteins may be important to the success of a T cell-based vaccine strategy. In this study, three quarters of vaccinees developed T-cell responses to two or more HIV-1 antigens detectable by stimulation with pools of global potential T-cell epitopes. The induction of both IL-2 and IFN-γ secreting cells among the CD4^+^ and CD8^+^ T-cell populations responding to the vaccine indicates that the vaccine induces T cells representing a variety of functional characteristics, including both MHC class I-restricted cytotoxic and class II-restricted helper cells.

A Phase IIb proof-of-concept trial (the Step study) evaluating an HIV-1 *gag/pol/nef* vaccine using a different adenoviral vector developed by Merck Research Laboratories was stopped early after an interim efficacy analysis found the vaccine to be ineffective, despite inducing frequent and diverse immune responses [31]. Moreover, although in that study no statistically significant differences in incidence of HIV infection were observed in the vaccine vs. placebo group, based on exploratory subgroup analyses uncircumcised men with prior Ad5 nAbs have been excluded in further trials with Ad5. Of note, unlike the VRC Ad5 vaccine investigated in the current study and the canarypox vector that was used in the Thai trial, the Merck vaccine did not include HIV Env antigens. Although immune correlates of protection in the Thai trial are not yet known, that study found CD4^+^ T cell and antibody responses against HIV Env antigen in vaccine recipients [2]. In addition, the specific deletions (E1, E3, E4) used to render the VRC adenovector replication defective may have different effects on vaccine immunogenicity compared to the E1-deleted Merck vector. The addition of the E4 deletion has been found *in vitro* to result in lower levels of rAd5 expression than seen in E1,E3-deleted vectors, and an E1,E3,E4-deleted vector did not prevent vaccine “take”, as measured by increases in Ad5 neutralizing antibodies four weeks following vaccination in volunteers with prior Ad5 nAb [32].

The immune responses following a single injection in the current study (which was performed before the results of the Step study became available) appear to compare favorably with responses in studies of multiple dose regimens of adenovectors alone or in combination with DNA plasmids, but there are some differences. For example the Step study [19], [31] of three doses of an HIV-1 gag/pol/nef Ad5 vaccine found HIV-specific T cells recognizing one or more gene products in 86% of participants with Ad5 titers ≤200, with geometric mean ELISpot response magnitudes in the range of several hundred SFC/10^6^ PBMC at week 8, the primary immunogenicity time point [19]. Similarly, analyses of T-cell responses from clinical trial subjects who received Merck vaccine candidates that included an HIV-1 gag insert – either by DNA priming followed by rAd5 boosting, or by a homologous rAd5/rAd5 prime-boost regimen – found IFN-γ ELISpot response rates of 42% to 65% in subjects not stratified by prior Ad5 immunity, with evidence of higher response rates in those with prior Ad5 Nab titers <1∶200 [33], [34]. In an international Phase IIa study of a three-injection DNA plasmid priming series followed by a single boost with VRC-HIVADV014-00-VP (HVTN study 204), T-cell responses to Env and Gag peptides were seen in approximately half of participants, twice as many as responded to Pol [35]. Similarly, an East African Phase I/II study that compared a prime-boost regimen using the same DNA and rAd5 products to rAd5 alone found T-cell responses to Env to be several times more frequent than those to Pol following the prime-boost regimen, while rAd5 alone elicited more frequent responses to Pol (and less frequent responses to Gag) than did the DNA prime/rAd5 boost regimen [36], [37]. These results raise the possibility that the prominent responses to Pol seen in the current study, as well as in the much larger Step study [31], may be associated with the use of an adenoviral vector alone, and that choice of vaccine vector or prime-boost regimen, or both, may affect the relative responses to different vaccine antigens.

The high (approximately 85%) rate of vaccine-induced antibody responses detected by the Abbott HIV diagnostic assay even 12 months following vaccination, and lower (but non-zero) rates with BioRad and bioMerieux diagnostic assays illustrate the importance of appropriate screening algorithms for HIV infection in the setting of vaccine trials, as participants obtaining routine antibody screening outside of the study site are at risk of unblinding, or facing uninterpretable or false-positive results. In this study, participants were counseled throughout the trial not to obtain HIV testing outside of the clinical research units, which provided HIV testing through a central laboratory that was able to distinguish between vaccine-induced seropositivity and actual HIV infection. Although anti-Env binding and neutralizing antibodies were elicited with the VRC Ad5 vaccine, these responses were low in titer and not broadly neutralizing. They are lower than antibody responses induced by DNA priming prior to rAd5 boosting, vector prime and Env gp120 subunit protein boosts, and Env subunit boost alone [22], [38], [39], [40].

Limitations of this study include the use of an Ad5 nAb-seronegative study population, which, although intended to provide optimal sensitivity for assessing the safety of the vaccine in a small study population, would be expected to result in more frequent or higher magnitude T-cell responses than would be anticipated in volunteers with substantial pre-existing nAb immunity to Ad5. It is therefore not possible to extrapolate these results to individuals with prior antibody immunity to Ad5. However, larger studies indicate that vector immunity attenuates, but does not eliminate, immune responses. In earlier dose-escalation clinical trials of HIV-1 clade B *gag* Ad5 monovalent [41] and HIV-1 *gag/pol/nef* Ad5 trivalent [42] vaccines developed by Merck Research Laboratories, the frequency of injection-site reactions as well as systemic adverse effects was dose dependent, and systemic effects (but not local reactions) occurred more frequently in subjects with baseline Ad5 nAb titers <1∶200. The proportion of vaccine recipients mounting IFN-γ-secreting T cells recognizing HIV-1 antigens, and the magnitudes of these responses, were generally higher in those with lower baseline Ad5 nAb titers. However, the effect of prior vector immunity on vaccine response in these studies was diminished at higher doses [41], although not uniformly [42]. While response rates and magnitudes in Step participants with Ad5 titers ≤1∶18 appeared comparable to those with titers ≤1∶200, responses in those with titers >1∶200 appeared somewhat lower [19]. Analysis of immune responses from a Phase IIa clinical trial of a prime-boost regimen consisting of DNA plasmids followed by VRC-HIVADV014-00-VP (HVTN study 204) found that preexisting Ad5 neutralizing antibodies blunted both CD4^+^ and CD8^+^ T-cell responses to Gag, but did not produce a significant effect on Env responses [43].

Additional limitations of this study include its modest sample size, which leaves open the possibility of infrequent vaccine-associated adverse events that might be observed in a larger study, and the enrollment of participants at only US sites, where self-reporting of safety outcomes may differ from that in other locations.

To the extent that prior Ad5 nAb immunity dampens vaccine-induced T-cell responses, such an effect might potentially be addressed by priming with other viral vectors or with DNA plasmids followed by boosting with an Ad5 vector [36], [44], or by developing adenoviral vectors of alternative serotypes to which prior antibody immunity is less frequent. In any of these approaches, predicting the vector dose that optimizes safety, particularly in vector-naïve individuals, yet provides optimal immunogenicity will facilitate the design of clinical trials.

The current study supports the clinical tolerability of adenoviral vector vaccines in the absence of prior vector nAb immunity, and shows that a single, well tolerated 10^10^ PU dose of the VRC adenoviral vector can produce T-cell responses of high magnitude, breadth and durability, high-frequency CD8^+^ and CD4^+^ T-cell responses, as well as antibody responses, against HIV-1 antigens.

## Supporting Information

Checklist S1 CONSORT Checklist.
(0.22 MB DOC)

Protocol S1 Trial Protocol.
(0.83 MB PDF)
