# Supplementary material for: Safety and Immunogenicity of a Replication-Defective Adenovirus Type 5 HIV Vaccine in Ad5-Seronegative Persons: A Randomized Clinical Trial (HVTN 054)
Source: PLoS One. 2010 Oct 27;5(10):e13579. doi: 10.1371/journal.pone.0013579 (PMC2965084; doi:10.1371/journal.pone.0013579)
Supplement: Protocol S1 — Trial Protocol. (0.83 MB PDF) [file pone.0013579.s002.pdf]

This protocol is being provided in support of the manuscript, "Safety and immunogenicity of an adenovirus type 5 HIV-1 vaccine in persons without prior vector immunity: A randomized clinical trial (HVTN 054)". For any other use, please contact template@hvtn.org.

PROTOCOL
HVTN 054

A Phase I dose-escalation clinical trial to evaluate the safety and immunogenicity of a multiclade, multivalent recombinant adenoviral vector HIV vaccine, VRC-HIVADV014-00-VP, in healthy, HIV-1 uninfected adult participants who have low titers of pre-existing Ad5 neutralizing antibodies

BB IND #11661 HELD BY DAIDS

CLINICAL TRIAL SPONSORED BY

Division of AIDS (DAIDS)
National Institute of Allergy and Infectious Diseases (NIAID)
National Institutes of Health (NIH)
Department of Health and Human Services (DHHS)
Bethesda, Maryland, USA

VACCINE PROVIDED BY

Dale and Betty Bumpers Vaccine Research Center (VRC), NIAID, NIH
Bethesda, Maryland, USA

FOR COPYING ONLY

December 6, 2004

This page is intentionally blank

FOR COPYING ONLY

Brief contents

Detailed contents 4
Tables and figures 7
Schema 8
Overview 9
Introduction 11
Ethical considerations 12

Study products

1 Study product background 16
2 Study product descriptions 20
3 Preclinical studies 23
4 Clinical study 27
5 Summary 28

Study design

6 Study objectives 32
7 Study type, study population, and eligibility criteria 33
8 Safety and immunogenicity evaluations 36
9 Statistical considerations 40

Study operations

10 Protocol conduct 48
11 Informed consent 49
12 Procedures 51
13 Study product preparation and administration 54
14 Safety monitoring and review 57
References 62
Protocol history 68
Protocol team 69

Appendices

Appendix B: Schedule of laboratory procedures 72
Appendix C: Schedule of procedures at HVTU 73

FOR COPYING ONLY

Detailed contents

Brief contents 3
Tables and figures 7
Schema 8
Overview 9
Introduction 11
Ethical considerations 12

Study products

1 Study product background 16
1.1 Design concept of a multiclade adenoviral vector vaccine for HIV-1 16
1.2 Previous human experience with recombinant adenoviral vector vaccines 17
1.2.1 Safety 17
1.2.2 Immunogenicity 19
1.3 Expected distribution of Ad5 neutralizing antibody titers 19

2 Study product descriptions 20
2.1 Recombinant adenoviral vector product: VRC-HIVADV014-00-VP 20
2.1.1 Production of the *gag-pol* adenoviral vector 21
2.1.2 Production of *env* adenoviral vectors 21
2.1.3 All four adenoviral vectors 22
2.1.4 Preparation of the bulk plasmid, final products and placebo 22

3 Preclinical studies 23
3.1 Preclinical safety studies 23
3.1.1 GLP toxicology study of VRC-HIVADV014-00-VP in rabbits 23
3.1.2 GLP biodistribution study for VRC-HIVADV014-00-VP in rabbits 24
3.2 Preclinical immunogenicity studies 25
3.2.1 Immune responses to VRC-HIVADV014-00-VP vaccine in mice 26
3.2.2 Immune responses to viral gene products in rabbits 26
3.2.3 Immunogenicity of recombinant adenoviral vector immunization of Cynomolgus macaques (VRC 03-060) 26

4 Clinical study 27
4.1 VRC Phase I study, Protocol VRC 006 27

5 Summary 28
5.1 Rationale for trial design 28
5.2 Plans for future product development and testing 28

Study design

6 Study objectives 32
6.1 Primary objectives 32
6.2 Secondary objectives 32

7 Study type, study population, and eligibility criteria 33

8 Safety and immunogenicity evaluations 36
8.1 Initial safety evaluation 36
8.2 Safety considerations for dose escalation 36
8.3 Screening for adenoviral vector recombinants 36
8.4 Distinguishing intercurrent HIV infection from vaccine-induced positive serology 36
8.5 Immunogenicity evaluation 37

FOR COPYING ONLY

8.5.1 Humoral immunogenicity studies ... 37
8.5.1.1 Binding antibodies by ELISA (HVTN) ... 37
8.5.1.2 Neutralizing antibody assay (HVTN) ... 37
8.5.2 Cellular immunogenicity studies ... 38
8.5.2.1 IFN-γ ELISpot (HVTN) ... 38
8.5.2.2 Intracellular cytokine staining (HVTN and VRC) ... 38
8.6 HLA typing ... 38
8.7 Ancillary studies ... 38
9 Statistical considerations ... 40
9.1 Overview ... 40
9.2 Objectives ... 40
9.3 Endpoints ... 40
9.3.1 Safety ... 40
9.3.2 Immunogenicity ... 40
9.3.3 Social impacts ... 40
9.4 Accrual and sample size ... 41
9.4.1 Sample size calculations for safety ... 41
9.4.2 Sample size calculations for immunogenicity ... 41
9.5 Statistical analysis ... 42
9.5.1 Analysis variables ... 42
9.5.2 Baseline comparability ... 42
9.5.3 Safety analysis ... 42
9.5.4 Immunogenicity analysis ... 43
9.5.5 Social impact analysis ... 44
9.5.6 Interim analyses ... 44
9.6 Randomization of treatment assignments ... 44

Study operations

10 Protocol conduct ... 48
11 Informed consent ... 49
11.1 Screening consent form ... 49
11.2 Protocol-specific consent form ... 49
11.3 Assessment of understanding ... 49
12 Procedures ... 51
12.1 Pre-enrollment procedures ... 51
12.2 Post-enrollment procedures ... 51
12.3 Total blood volumes ... 53
12.4 Laboratory procedures ... 53
13 Study product preparation and administration ... 54
13.1 Schema and vaccine regimen ... 54
13.2 Study product formulation and preparation ... 54
13.2.1 VRC-HIVADV014-00-VP (Multiclade HIV-1 Recombinant Adenoviral Vector Vaccine, rAD) ... 54
13.2.2 VRC-DILUENT013-DIL-VP (Final Formulation Buffer, FFB, Placebo) ... 55
13.2.3 Study product preparation ... 55
13.2.4 Labeling procedures to preserve blinding ... 55
13.3 Study product administration ... 55
13.4 Study product acquisition ... 55
13.5 Pharmacy records ... 56
14 Safety monitoring and review ... 57

FOR COPYING ONLY

14.1 Assessing reactogenicity 57
14.2 Grading of adverse experiences 57
14.3 Adenovirus culture 57
14.4 Adverse experience reporting and safety pause 58
14.4.1 Follow up and resolution after a safety pause 58
14.4.2 Reporting serious adverse experiences to RCC and SCHARP 58
14.5 Participant termination from the study 59
14.6 Study termination (for all participants) 59
14.7 HVTN review of cumulative safety data 59
14.7.1 Daily review 60
14.7.2 Weekly review 60
14.7.3 Monthly review 60
14.7.4 Quarterly review 60
14.7.5 Review for dose escalation 61
References 62
Protocol history 68
Protocol team 69

Appendices

Appendix B: Schedule of laboratory procedures 72
Appendix C: Schedule of procedures at HVTU 73

FOR COPYING ONLY

Tables and figures

Table 3-1 Summary of number of rabbits with positive findings in six tissue types and average number of copies of target/μg DNA 25
Table 7-1 Study inclusion criteria 34
Table 7-2 Study exclusion criteria 35
Table 9-1 Probability (Pr) of response for different safety and immunogenicity scenarios 41
Table 12-1 Pre-enrollment procedures 52
Table 12-2 Post-enrollment procedures 52
Table 14-1 Schedule of reactogenicity assessments 57
Table 14-2 Adverse experience notification and safety pause rules 59
Protocol history and modifications 68

FOR COPYING ONLY

Schema

Study products

Vaccine: Recombinant adenoviral vector VRC-HIVADV014-00-VP (Clade B Gag and Pol; Clades A, B,C Env)

Placebo: Adenoviral vector final formulation buffer VRC-DILUENT013-DIL-VP

Administration: 1 mL intramuscular injection via needle and syringe given as a single dose on Day 0

Study arm	Number	Dose	Administration
Group 1	20	10^{10} PU	vaccine
	4		placebo
Group 2	20	10^{11} PU	vaccine
	4		placebo
Total	48		

Groups 1 and 2 will be enrolled sequentially, following evaluation of safety data (see Section 8.2).

PU = particle units

FOR COPYING ONLY

Overview

Title

A Phase I dose-escalation clinical trial to evaluate the safety and immunogenicity of a multiclade, multivalent recombinant adenoviral vector HIV vaccine, VRC-HIVADV014-00-VP, in healthy, HIV-1 uninfected adult participants who have low titers of pre-existing Ad5 neutralizing antibodies

Participants

Healthy HIV-1-uninfected adult participants (18 to 50 years old) who have preexisting Ad5 neutralizing antibody titers of <1:12

Number of participants

Total 48: 40 vaccine, 8 placebo

Primary objectives:

To characterize the safety and tolerability of a single dose of the adenoviral vector vaccine delivered at each of two escalating doses (10^{10} PU and 10^{11} PU IM) in participants with low (<1:12) titers of pre-existing adenovirus, type 5 (Ad5) neutralizing antibodies.

Study products

Recombinant Adenoviral Vector Vaccine: VRC-HIVADV014-00-VP

VRC-HIVADV014-00-VP is a recombinant product composed of 4 adenoviral vectors (Ad) (in a 3:1:1:1 ratio) that encode the HIV-1 Gag/Pol polyprotein from Clade B (HXB2/NL4-3) and HIV-1 Env glycoproteins from Clades A (92rw020), B (HXB2/Bal), and C (97ZA012).

Placebo

The placebo for the VRC-HIVADV014-00-VP is adenoviral vector final formulation buffer (FFB), VRC-DILUENT013-DIL-VP.

Study design

Multicenter, randomized, placebo-controlled, double-blind trial

Study duration

12 months per participant

Safety monitoring

HVTN 054 Protocol Safety Review Team

HVTN Safety Monitoring Board

Vaccine provider

Dale and Betty Bumpers Vaccine Research Center, National Institute of Allergy and Infectious Diseases (NIAID), National Institutes of Health (NIH) (Bethesda, Maryland, USA)

Sponsor

Division of AIDS (DAIDS), NIAID, NIH, Department of Health and Human Services (DHHS) (Bethesda, Maryland, USA)

FOR COPYING ONLY

IND holder

DAIDS, NIAID, NIH

Study sites

Continental US HIV Vaccine Trial Units (HVTUs)

HVTN Core Operations

HVTN Core Operations Center, Fred Hutchinson Cancer Research Center (FHCRC) (Seattle, Washington, USA)

Statistical and data management center

Statistical Center for HIV/AIDS Research and Prevention (SCHARP), FHCRC

Central laboratories

Duke University Medical Center (Durham, North Carolina, USA)

FHCRC/University of Washington

Viral and Rickettsial Disease Laboratory (Richmond, California, USA)

South Africa Immunology Laboratory - National Institute for Communicable Disease (Johannesburg, South Africa)

FOR COPYING ONLY

Introduction

The ongoing worldwide epidemic of the human immunodeficiency virus type 1 (HIV-1) remains one of the major global health challenges. HIV-1 causes the acquired immunodeficiency syndrome (AIDS), which is responsible for tremendous human suffering and economic loss throughout the world. Currently, over 42 million people are living with HIV-1 infection [1]. Without treatment, it is likely that nearly all of these will die of AIDS in the next 2 decades. Over 900,000 people are living with HIV infection in the US [2], but only 3/4 are aware of their infection [3], and incident infections remain extremely high at more than 40,000 per year [4].

Since 1996, potent new antiretroviral therapies, including combination regimens with protease inhibitors, have created the possibility that HIV-1 infection might become a chronic, manageable disease among individuals with access to these medications. In the US, AIDS deaths are down to 16,000 per year as a result of the new antiretrovirals [5].

However, for the developing world, where over 95% of the 5 million annual incident HIV-1 infections occur [1], it is unlikely that these drugs will be widely accessible, due to many logistical challenges associated with their use. Globally, more than 3 million AIDS deaths occur per year [1], and nearly 20 million have died since the HIV epidemic began [2]. AIDS is the leading killer in Africa, with over 28 million Africans living with HIV/AIDS. Sub-Saharan Africa has been affected most; in 7 Sub-Saharan African countries, over 20% of adults (aged 15-49) are living with HIV/AIDS [2]. For example, in Botswana, 38.8% of adults aged 15 to 49 are infected with HIV, while in South Africa 24.8% of women in antenatal clinics are infected [2]. Around the world, 14,000 new infections occur each day, and AIDS has become the leading infectious disease killer, and the fourth leading cause of death overall. In severely affected countries, life expectancy has fallen by more than 10 years [1].

The need for better education, better treatment access, better prevention programs, and better prevention technologies is therefore clear. Specifically, the need for a safe, effective, and affordable HIV-1 vaccine is paramount [6,7]. The ideal HIV-1 vaccine for global use should meet several of the following criteria:

- proven safety in healthy HIV-uninfected persons
- induction of long-lasting HIV-specific cell-mediated and humoral immunity capable of conferring protection against HIV
- tolerability
- potential for production in sufficient quantity to meet global needs
- affordability
- stability during distribution and storage

FOR COPYING ONLY

Ethical considerations

Multiple candidate HIV vaccines will need to be studied simultaneously in different populations in the United States and around the world before a successful HIV preventive vaccine is found. It is critical that universally accepted ethical guidelines are followed at all sites involved in the conduct of these clinical trials. The HVTN has addressed ethical concerns in the following ways:

- HVTN trials are designed and conducted to enhance the knowledge base necessary to find a preventive vaccine, with methodology that is scientifically rigorous and valid, and in accordance with Good Clinical Practice (GCP) guidelines.
- HVTN scientists and protocol team members incorporate the philosophies underlying major codes, declarations, and other guidance documents relevant to human subject research into the design and conduct of HIV vaccine clinical trials.
- HVTN scientists and protocol team members are committed to substantive community input into the planning, conduct, and follow up of the research which will help ensure that locally appropriate cultural and linguistic needs of study populations are met.
- The HVTN advocates that all HVTN sites should develop a plan for the care and treatment of participants who develop HIV infection during a trial. This plan should be formulated by representatives of host countries, communities from which potential trial participants will be drawn, sponsors, and the HVTN.
- Prior to implementation, HVTN trials are rigorously reviewed by both local and national regulatory bodies, in addition to scientists who have no involvement with the trial under consideration.
- The HVTN recognizes the importance of institutional review and values the role of in-country Institutional Review Boards (IRBs) and Institutional Ethics Committees (IECs) as custodians responsible for ensuring the ethical conduct of research in the local setting.
- The HVTN provides training so that all participating sites similarly ensure fair subject selection, protect the privacy of research subjects, and obtain meaningful informed consent.

FOR COPYING ONLY

This page is intentionally blank

FOR COPYING ONLY

STUDY PRODUCTS

FOR COPYING ONLY

1 Study product background

1.1 Design concept of a multiclade adenoviral vector vaccine for HIV-1

The recombinant adenoviral (rAd) vaccine product design is based on the concept of immunization by gene delivery. Recombinant adenoviral vector vaccines offer the positive attributes of immune stimulation inherent in live attenuated vaccines, without adjuvant, and without utilizing HIV-1 as the attenuated virus. The intended use for VRC-HIVADV014-00-VP is as a preventive HIV-1 vaccine.

Cellular immune responses play a vital role in containing HIV-1 replication, are temporally associated with declining HIV-1 viremia in acute infection and are often present at high levels during chronic HIV-1 infection [8-12]. VRC-HIVADV014-00-VP was designed with the rationale that effective preventive vaccines will have to stimulate strong virus-specific CD8+ cytotoxic T-lymphocytes (CTL) immune responses. The importance of CD8+ CTL in controlling SIV replication in rhesus monkeys has been directly established by studies in which *in vivo* depletion of CD8+ T-cells resulted in a rapid and dramatic increase in plasma viremia [13,14]. The importance of CD4+ helper T lymphocyte responses in controlling HIV-1 replication has also been established, however in contrast to robust CTL responses, HIV-1-specific CD4+ T lymphocyte responses are typically weak or absent in most chronically HIV-1-infected individuals, partly because HIV-1 preferentially infects and destroys HIV-specific CD4+ T cells [15]. A vaccine strategy that can stimulate a balanced CD4+ and CD8+ response is most likely to control HIV-1 infection upon challenge.

Preclinical evidence indicates that VRC-HIVADV014-00-VP elicits cellular immune responses against HIV-1 in mice, rabbits and monkeys by direct gene delivery of immunogen-expressing HIV genes via rAd vectors. Data from the preclinical studies with the investigational vaccine are summarized briefly in Section 3 of the protocol and in greater detail in the Investigator Brochure. The preclinical immunogenicity studies suggest that a rAd vaccine containing genes that express Gag and Pol in combination with HIV envelope proteins may induce a significant immune response in humans.

The major advantage of adenoviral vector immunization appears to be its efficacy in the induction of $CD8^+$ CTL responses, considered an important element in controlling HIV-1 viral replication [11,13,14,16-19]. There is an additional safety feature in that following entry into the target cells, the HIV-1 gene products will be produced without the production of infectious adenovirus. These gene products can be produced in cells that are not actively dividing. Studies in non-human primates have shown that replication-deficient serotype 5 adenovirus vectors (Ad5) can generate cellular immune responses against several viruses including HIV-1, simian-human immunodeficiency virus (SHIV) and Ebola [20-25]. Baboons immunized with 10^{11} particles of replication-deficient Ad5 have been shown to have strong cellular immune responses evidenced by gag-specific T-cells that were quantified by enzyme-linked immunospot (ELISpot) assay [23]. Rhesus macaques immunized with SIV Gag-based Ad5 vectors showed potent cytotoxic T lymphocyte (CTL) responses that correlated with protection (reduced CD4+ loss, contained acute and chronic viremia and reduced morbidity and mortality when challenged with a pathogenic strain of SIV) [21].

There is concern that because of the prevalence of pre-existing neutralizing antibodies to adenoviruses in the adult population, the utility of adenoviral vectors in humans may be limited. Merck Research Laboratories report that preclinical studies show priming for HIV-1 specific immunity using an adjuvant-formulated DNA vaccine followed with Ad5 vaccine

FOR COPYING ONLY

boost, generates levels of T-cell immune response that are comparable to those in adenovirus-naive animals receiving multiple high doses of Ad5 HIV-1 vaccines [25]. Cynomolgus macaques immunized with a combination DNA plasmid (a mixture of four DNA plasmids encoding glycoproteins from three Ebola strains and nucleoprotein from one strain) and boosted with a replication-deficient adenoviral vector encoding the glycoprotein resisted lethal viral challenge [22]. These studies suggest that replication-defective adenoviral vectors, as part of a carefully crafted vaccine strategy, can elicit potent and protective T-cell immune responses that may control HIV-1 and other viral infections.

Future clinical development plans for VRC-HIVADV014-00-VP include using it in a regimen of three DNA vaccine primes followed by a single rAd vaccine boost. Combination modality regimens using a DNA vaccine prime followed by a viral vector boost have shown promise in non-human primate models of HIV infection. Such regimens have the potential for raising high levels of immune responses. DNA vaccine priming followed by a recombinant viral vector boost with a modified vaccinia Ankara (rMVA) [26] or replication-deficient Ad5 [21] have been shown to attenuate a pathogenic SHIV infection in rhesus macaques, most likely by the generation of a $CD8^+$ CTL response.

A major challenge in the design of rAd HIV vaccines is to identify and target viral structures that are the critical determinants for protective humoral and cellular responses across the widest possible range of diversity. The use of multivalent vaccines, containing a defined mixture of immunogens from a number of prevalent subtypes might be a feasible approach to achieve broadly protective HIV vaccines. The World Health Organization UNAIDS HIV Vaccine Advisory Committee has recommended that candidate HIV vaccines be designed based upon the strains prevalent in the country in which trials are to be conducted [27]. This approach is the foundation for the design of the investigational vaccine VRC-HIVADV014-00-VP, which is composed of a combination of four rAd that incorporate HIV *gag, pol* and *env* genes. The Gag protein along with the highly conserved Pol protein are derived from a Clade B strain of HIV-1; the Env proteins are derived from Clades A, B and C, which together represent the viral subtypes responsible for about 90% of new HIV infections in the world [28]. The Vaccine Research Center, NIAID, NIH and the World Health Organization-Joint United Nations Programme on HIV/AIDS organized a meeting focused on the genetic diversity of HIV and strategies to develop vaccine candidates. A consensus was reached that generation of multiclade candidate vaccines is a high international scientific priority [29].

1.2 Previous human experience with recombinant adenoviral vector vaccines

1.2.1 Safety

Replication-competent adenovirus vaccines in oral form have been administered to millions of military personnel, and over 42,000 have participated in well-controlled clinical trials to evaluate the safety and efficacy of these agents. These studies are well summarized in the literature [30-34] and have shown these vaccines to be safe and highly effective in preventing acute respiratory disease in recruits. The potential risk for live adenovirus to cause serious infection in immunocompromised hosts and potential concerns for oncogenic potential of some serotypes have lead to the development of replication-defective adenoviral vectors as vaccines and gene therapy vectors.

Adenoviral vectors incorporating a variety of expressed proteins have been used for gene therapy studies in many different human diseases, including cancer, cystic fibrosis, and cardiovascular disease, and have been delivered via several routes of administration including

FOR COPYING ONLY

aerosol, intradermal, intramyocardial, intrapleural, intravenous, and intratumoral [35-49]. Hundreds of human subjects have taken part in studies to evaluate adenoviral vectors as gene therapy agents, with many of these evaluating vectors based upon adenovirus type 5. Data from these and other studies found that side effects of adenoviral vectors were minor, local, or absent in most cases where the agents were administered intradermally or intramuscularly, with no significant vector-induced toxicities. One study assessed ten clinical trials for the safety parameters [46] and risk factors [47] of low ($<10^9$ particle units, PU)- and intermediate (10^9-10^{10} PU) – dose adenoviral vectors, delivered by various routes (nasal, bronchial, percutaneous injection into solid tumor, intradermal, epicardial injection of myocardium, and injection of skeletal muscle) to 90 individuals and 12 controls for treatment of a variety of conditions (cystic fibrosis, colon cancer metastases, severe coronary artery disease, and peripheral vascular disease). Local administration of these doses of adenoviral vectors appeared to be well tolerated. The major adverse events seemed to be primarily associated with characteristics of the study population (age, co-morbid conditions) and/or trial procedures (surgery) rather than dose, route of administration, expressed transgene, or number of administrations.

In the context of a large body of clinical research experience with adenovirus-based vectors, the death of a teenager in a gene therapy trial at the University of Pennsylvania prompted extensive reviews of safety data from both human and animal studies of these agents, many at the direction of the NIH Recombinant DNA Advisory Committee (RAC) and the Food and Drug Administration (FDA). The 18 year-old patient, suffering from ornithine transcarbamylase deficiency (OTCD), died after receiving a dose of 3.8 x 10^{13} PU of an E1/E4–deleted serotype 5 adenoviral vector directly into the hepatic artery [50]. An NIH report summarizing a review of clinical data from the case concluded that the participant’s death was most likely due to a systemic adenovector-induced shock syndrome, caused by a cytokine cascade that led to disseminated intravascular coagulation, acute respiratory distress, and multiorgan failure. Post-mortem bone marrow biopsy revealed red cell aplasia. The data suggested that the high dose of adenovector delivered directly to the liver quickly saturated available receptors for the vector in that organ and disseminated systemically to induce the fatal immune response [51].

In the University of Pennsylvania study, 19 OTCD patients received doses ranging from 1.86 x 10^{11} to 3.8 x 10^{13} E1/E4–deleted adenoviral vector particles infused directly into the liver. The trial was halted after the death described above. Essentially all study subjects experienced one or more of the following: fevers, myalgias, nausea, and occasional emesis. Nearly all subjects showed a mild and transient thrombocytopenia without consistent abnormalities in coagulation, and higher dose levels were associated with subsequent abnormal liver function studies [50]. Similar results have been observed in animal studies [52-57] with the implication that systemic administration of adenoviral vectors might cause liver abnormalities and fever and might be associated with these symptoms more often than in patients treated via other routes of administration. Close clinical monitoring to detect such symptoms will be carried out in the present study, as will careful screening and exclusion of participants with hepatitis C and active hepatitis B. The adenoviral vectors will be administered by intramuscular injection, with a maximum study dose of 10^{11} PU.

Adenoviral vector-mediated gene transfer by intradermal injection [38,47,48] has been tested in healthy volunteers, in an effort to define the normal host responses and persistence of the vector. Six healthy volunteers received a single 8 x 10^7 or 8 x 10^8 PU intradermal injection of an $E1^-$, $E3^-$ adenovirus type 5-based vector carrying the *E. coli* cytosine deaminase gene (Ad_{GV}CD.10) [38,48]. One additional subject received three administrations of vector, and two others received a single administration with concomitant oral prednisone. No adverse

FOR COPYING ONLY

effects were observed in any of the nine participants that could be attributed to the vector [47]. Detailed cellular reactogenicity was described for a subset of the participants [38]. Skin induration was observed at the injection site in six participants, peaking at day 3 and gradually declining by day 14. Mild/moderate local cellular responses were observed in skin biopsies as measured by cellular infiltration in skin biopsy specimens at all doses studied and adenoviral DNA could be detected in tissue collected 18 days after injection.

The effect of pre-existing immunity on the safety and tolerability of adenoviral vector vaccines is unclear. In macaques receiving a relatively high dose of an adenoviral vector vaccine directly into the portal circulation, prior immunity did not eliminate systemic vector-induced toxicity [52]. Dose-escalation trials of HIV-1 Gag-Ad5 vaccines administered by intramuscular injection in humans found moderate and sporadic injection site reactions, as well as sporadic fever with malaise, chills, and body aches, apparently more common at higher doses of the vaccine [58,59]. These adverse events, which resolved within 48 hours, were more common in participants with low Ad5 antibody titers at baseline, and were attenuated following a booster dose of the adenoviral vector [59]. Data from several studies suggest that approximately one-third of adults in developed countries will have Ad5 neutralizing antibodies <1:20 [48,58-61]. The proportion of seronegatives for Ad5 in developing countries appears to be lower [61-63].

1.2.2 Immunogenicity

Reports from Phase I clinical trials of recombinant adenovirus type 5 (Ad5) vector vaccines developed by Merck & Co., Inc. and encoding Clade B HIV-1 Gag described the vaccines as well tolerated [58,59]. ELISpot responses 4 weeks following the second of two injections of one vector, at doses ranging from 10^8 to 10^{11} PU per dose, were reported in between 43% and 91% of participants grouped by pre-existing Ad5 antibody titer and dose level. Responses to the Ad5 HIV-1 gag vaccine appeared to be influenced by pre-existing humoral immunity to Ad5 at all dose levels. The immunogenic effect of increasing the vaccine dose appeared most marked in the high (>1:200) titer pre-existing Ad5 antibody group, but the number of subjects was small [59].

1.3 Expected distribution of Ad5 neutralizing antibody titers

The limited data currently available suggest that pre-existing immunity to Ad5 may attenuate the immune response to an Ad5 vector vaccine. Although such attenuation has been demonstrated in mice [64,65], little clinical data are available on the correlation between antibody titer and magnitude of vaccine response. Moreover, extensive population data on the distribution of neutralizing antibodies (NAb) to Ad5 are limited. Ad5 is endemic in the regions where it causes infection, with one study finding antibody to Ad5 in virtually all subjects screened, although only 55% of these antibodies were found to be neutralizing [66]. In an analysis of 117 children attending a day-care center studied between 1967 and 1981, 59% had NAb to Ad5 (titer ≥1:10) by age 5 [67]. A study that included 47 mothers of children with cystic fibrosis and a comparison group of 33 additional adult volunteers found that 48.9% of mothers were seropositive (titer > 3.5log2) for NAb to Ad5, with geometric mean titer (GMT) = 5.5 log2 (SD = 1.2). Of the other adults, 27.3% were seropositive for NAb to Ad5, with GMT = 6.6 log2 (SD=1.5) [68]. Data from these studies and from other small cohorts, in addition to the Merck Ad5-based HIV-gag vaccine studies mentioned above, suggest that at baseline roughly one third of adults in developed countries will be seronegative (titer <1:10 - 1:20) for Ad5-NAb, and roughly one third will have titers above the 1:200 range [48,58-61]. Considerable variation exists among studies, however, with some reporting detectable NAb to Ad5 in as few as one third of U.S. subjects [69,70].

FOR COPYING ONLY

2 Study product descriptions

2.1 Recombinant adenoviral vector product: VRC-HIVADV014-00-VP

The recombinant adenoviral vector product VRC-HIVADV014-00-VP (rAd) is a replication-deficient, combination vaccine containing four recombinant serotype 5 adenoviral vectors. These vectors contain gene sequences that code for Clade B HIV-1 Gag and Pol as well as Clade A, Clade B, and Clade C Env protein. *In vivo* expression by these vectors produces immunogens that induce an immune response against HIV. The envelope genes were chosen as representative primary isolates from each of the three clades.

The process for constructing the four VRC-HIVADV014-00-VP recombinant adenoviral vectors is based upon a rapid vector construction system (AdFAST™, GenVec, Inc.) used to generate adenoviral vectors that express the four HIV antigens gp140(A), gp140(B)dv12, gp140(C) and GagPol(B) driven by the cytomegalovirus (CMV) immediate-early promoter. Manufacturing is based upon production in a proprietary cell line (293-ORF6), yielding adenoviral vectors that are replication deficient. The vectors are purified using CsCl gradient centrifugation. The product is formulated as a sterile liquid injectable dosage form for intramuscular injection.

The GV11 adenoviral backbone was chosen to reduce the risk of replication-competent adenovirus (RCA) generation during clinical production. The GV11 backbone contains deletions of two essential regions, E1 and E4, as well as a partial E3 deletion that render the vaccine product replication-deficient. The generation of RCA would require two independent recombination events in a single adenovirus genome, predicted to be an extremely rare event [71].

The Ad_{GV} (HIV).11D vectors contain HIV-1 antigen open reading frame (ORF) expression cassettes inserted to replace the deleted adenovirus E1 gene region. Other deleted adenovirus regions include a partial E3 and all of E4, which has been replaced with a transcriptionally inert spacer element (T1S1) that enhances production of the adenoviral vectors [72].

The 293-ORF6 cell line used to propagate these E1, E4 and partial E3 deleted vectors was developed at GenVec, Inc. These cells were constructed by stably transforming 293 cells (which are of human embryonic kidney origin) with an inducible E4-ORF6 expression cassette. This enables the cells to efficiently complement the E1-, E4-, and partial E3-deleted adenoviral vectors, provide increased transgene capacity and greatly reduce the potential to generate replication-competent adenovirus. The particular clone that has given rise to the cell line is the A232 clone. All references to the 293-ORF6 cell line refer to cells derived from the original A232 clone. This replication-deficient adenoviral vector system has been used to produce TNFerade, a TNF-alpha gene-based delivery product [73]. An assay for replication-competent adenovirus (RCA) is performed in the final release testing for all vectors; RCA has not been observed in this packaging system during the manufacture of multiple gene-based products.

The four vaccine adenoviral vectors are generated by introducing a DNA plasmid consisting of the adenoviral genome into the 293-ORF6 cells. The adenoviral vector in the lysate from the transfected cells is serially passaged to expand the titer of adenoviral vector. The identity and integrity of the passages is verified by PCR and expression of the HIV-1 gene is confirmed by Western Blot analysis. Purified adenoviral vector is produced by infecting the 293-ORF6 cells with the adenoviral vector in the lysate; after the infection of the cells is complete, the material is collected and the vector is purified from the cells. The four vaccine

FOR COPYING ONLY

adenoviral vectors are purified using a cesium choride (CsCl) gradient centrifugation process. CsCl is removed by dialyzing the virus preparation against the final formulation buffer (VRC-DILUENT013-DIL-VP). Purified adenoviral vector serves as a vector bank for subsequent production of the four vaccine adenoviral vectors. This vector bank is tested for sterility, mycoplasma and other adventitious agents prior to its being used for manufacturing of clinical supplies.

2.1.1 Production of the *gag-pol* adenoviral vector

AdtGagPol(B).11D

The protein sequences of the Gag and Pol proteins from an HIV-1 Clade B were used to create a synthetic polyprotein version of the *gag-pol* genes using codons optimized for expression in human cells. The synthetic *gag* gene is from HIV-1 Clade B strain HXB2 (GenBank accession number K03455), and the synthetic *pol* gene (*pol/h*) is from HIV-1 Clade B NL4-3 (GenBank accession number M19921). The *pol* gene is nonfunctional because it is present as a fusion protein. Mutations were introduced in the synthetic protease and reverse transcriptase genes. The protease modification prevents processing of the *pol* gene product, and reduces the potential for functional protease, reverse transcriptase and integrase enzymatic activity. The cDNA used to produce AdtGagPol(B).11D is similar to an HIV-1 DNA vaccine VRC-4302 (BBIND 9782) which was tested and shown to have no reverse transcriptase activity. No modifications were made to the *gag* gene. To construct the adenoviral vector, the HIV-1 DNA sequence was subcloned using standard recombinant DNA techniques into an expression cassette in an E1-shuttle plasmid.

2.1.2 Production of *env* adenoviral vectors

Adgp140(A).11D

The protein sequence of the envelope polyprotein (gp160) from 92rw020 (CCR5-tropic, GenBank accession number U08794) was used to create a synthetic version of the gene (Clade-A gp140delCFI) using codons altered for expression in human cells. Plasmids expressing the HIV-1 genes were made synthetically with sequences designed to disrupt viral RNA structures that limit protein expression by using codons typically found in human cells. To construct the adenoviral vector, the HIV-1 DNA sequence was subcloned using standard recombinant DNA techniques into an expression cassette in an E1-shuttle plasmid.

Adtgp140dv12(B).11D

The protein sequence of the envelope polyprotein (gp160) from HXB2 (X4-tropic, GenBank accession number K03455) was used to create a synthetic version of the gene (X4gp160/h) using codons optimized for expression in human cells. To produce an CCR5-tropic version of the envelope protein (R5gp160/h), the region encoding HIV-1 envelope polyprotein amino acids 275 to 361 from X4gp160/h (VRC3300) were replaced with the corresponding region from the BaL strain of HIV-1 (Genbank accession number M68893, again using human preferred codons). The full-length CCR5-tropic version of the envelope protein gene from pR5gp160/h (VRC3000) was terminated after the codon for amino acid 680. The truncated Env glycoprotein (gp140) contains the entire surface protein and the ectodomain of gp41 including the fusion domain, and regions important for oligomer formation, specifically two helical coiled coil motifs. The Env V1 and V2 loops were deleted to improve the stability and yield of the vector in the producer cell line (G. Nabel, personal communication). Two additional amino acids were incorporated immediately after the deletion due to creation of a restriction enzyme site. In order to construct the adenoviral vector, the HIV-1 DNA sequence

FOR COPYING ONLY

was subcloned using standard recombinant DNA techniques into an expression cassette in an E1-shuttle plasmid.

Adgp140(C).11D

The protein sequence of the envelope polyprotein (gp140delCFI) from 97ZA012 (CCR5-tropic, GenBank accession number AF286227) was used to create a synthetic version of the gene (Clade-C gp140delCFI) using codons optimized for expression in human cells. To construct the adenoviral vector, the HIV-1 DNA sequence was subcloned using standard recombinant DNA techniques into an expression cassette in an E1-shuttle plasmid.

2.1.3 All four adenoviral vectors

The four E1-shuttle plasmids were recombined in *Escherichia coli* (*E. coli)* BjDE3 bacteria with the GV11 adenovector based AdFAST^{TM} plasmid pAdE1(BN)E3(10)E4(TIS1) to generate the adenoviral vector plasmids. The replication-deficient adenoviral vectors AdtGagPol(B).11D, Adgp140(A).11D, Adtgp140dv12(B).11D, and Adgp140(C).11D were then generated by introducing the adenoviral vector plasmid into the packaging cell line, 293-ORF6.

2.1.4 Preparation of the bulk plasmid, final products and placebo

The investigational vaccine, VRC-HIVADV014-00-VP, was manufactured by GenVec, Inc. (Gaithersburg, MD) at a contract manufacturer, Molecular Medicine (San Diego, CA). DNA plasmids produced by the Vaccine Research Center, NIAID, NIH (Bethesda, MD) were used to construct the adenoviral vector clinical seed stock. The Phase I clinical production for each adenoviral vector was performed by Molecular Medicine from clinical seed stock produced by BioReliance (Rockville, MD).

The multiclade adenoviral vector vaccine product, VRC-HIVADV014-00-VP, is a 3:1:1:1 ratio of the adenoviral vectors that encode for HIV-1 Gag/Pol polyprotein from Clade B and HIV-1 Env glycoproteins from Clades A, B, and C, respectively. Final product meeting all test specifications has been released for use in the proposed clinical study. Vials were filled to 1.2 mL volume with 1×10^{10} or 1×10^{11} particle units /mL.

The final formulation buffer (FFB) is custom manufactured by BioWhittaker (Frederick, MD). It is identified in this study as VRC-DILUENT013-DIL-VP and it will be used as the placebo. The FFB is composed of sodium chloride, Tris buffer, trehalose•$2H_2O$ (low endotoxin), magnesium chloride•$6H_2O$, monooleate (Tween 80) and water for injection (WFI).

FOR COPYING ONLY

3 Preclinical studies

3.1 Preclinical safety studies

The Investigator Brochure provides more extensive information about the preclinical safety studies. Briefly described here are the biodistribution and toxicology studies conducted in rabbits with VRC-HIVADV014-00-VP.

3.1.1 GLP toxicology study of VRC-HIVADV014-00-VP in rabbits

Gene Logic (formerly TherImmune Research Corporation, Gaithersburg, MD) conducted preclinical toxicology studies under Good Laboratory Practice (GLP) using lots of the four adenoviral vectors that are identical to those in the study vaccine (VRC-HIVADV014-00-VP). The vaccine is 50% (by weight) adenoviral vector for expression of Clade B Gag-Pol and 50% (by weight) adenoviral vectors for expression of Clades A, B and C Env. The objective was to assess the potential toxicity of VRC-HIVADV014-00-VP when administered at two time points by intramuscular injection into New Zealand White rabbits using needle and syringe. Ten animals/sex/group received 1.0 mL of Final Formulation Buffer (FFB, VRC-DILUENT013-DIL-VP) or $1x10^{11}$ PU (highest human dose to be tested) of VRC-HIVADV014-00-VP on Study Day (SD) 1 and SD 22. On SD 1, two 0.5 mL injections were administered into the right thigh muscle at injection site 1 and 2. On SD 22, two 0.5 mL injections were administered into the left thigh muscle at injection site 3 and 4. Necropsies were performed on five animals per sex per group per time point on SD 24 and SD 36.

Parameters evaluated included mortality, clinical signs of toxicity, Draize observations, body weights, body weight changes, food consumption, ophthalmologic examinations, clinical pathology (chemistry, hematology and coagulation), body temperatures, gross pathology, organ weights, and histopathology. Observations were also made of motor function and behavior during the study. Tissues analyzed included: adrenals, aorta, brain, cecum, colon, cervix, duodenum, epididymides, esophagus, eyes, femur, gall bladder, gross lesions, harderian glands, heart, ileum, injection sites, jejunum, kidneys, liver, lung, lymph nodes, mandibular salivary glands, mammary glands, optic nerves, ovaries, pancreas, pituitary, prostate, rectum, seminal vesicles, skeletal muscle, skin, spinal cord, spleen, sternum with marrow, stomach, sciatic nerve, testes, thymus, thyroid/parathyroid, trachea, tongue, urinary bladder, uterus, and vagina.

All animals in the repeat-dose toxicity survived until scheduled sacrifice. No significant changes in mortality, Draize observations, body weights, body weight changes, ophthalmologic findings, and organ weights (with the exception of an increased spleen weight, which is likely an expected result of exposure to an immunostimulatory agent) were observed other than sporadic findings that were not considered to be test article related. A statistically significant increase in mean body temperature was observed in Group 2 males on SD 2 (39.3°C vs 38.26°C in controls, 24 hours after administration of the first dose only of the test article). Also statistically significant, but of less difference, were increases in body temperatures in Group 2 females on SD 2 (38.99°C vs 38.46°C in controls) and in Group 2 males on SD 1 (38.72°C vs 38.4°C in controls) 3 hours post-inoculation after the first dose of the test article. A reduction in food consumption in both males and females was observed for a 24-48 hour period following each injection, but then returned to a level comparable to the controls. At least twice daily, cageside observations were made of the rabbits and there were no observations of movement impairment noted.

FOR COPYING ONLY

There was a statistically significant increase on SD 3 to values outside the historical normal range in mean serum cholesterol for males (102 vs 80 in controls) and in triglyceride levels for males and females on SD 3 (males – 287 vs 159 mg/dL in controls; females – 188 vs 126 mg/dL in controls), but not on SD 24 or SD 36. However, there was no associated pathology or clinical observations to correlate with these findings. On SD 24, there was a transient, statistically significant difference in mean serum creatine phosphokinase (CPK) in males (1498 vs 997 IU in controls), which was possibly correlated with muscle inflammation.

On the SD 24 necropsy, there was minimal to mild chronic and chronic/active inflammation at some of the four injection sites in treated animals of both sexes. Inflammation was seen in the SD 1 injection sites in 2/10 animals at injection site 1 and in 4/10 animals at injection site 2, compared to the SD 22 injection sites in which inflammation was seen in 6/10 animals at injection site 3 and in 5/10 animals at injection site 4. No inflammation was seen in control animals. The incidence and severity of inflammation at injection sites was reduced in the recovery animals by SD 36.

On the SD 24 necropsy perineural inflammation around the sciatic nerve was noted on the left side (right side not evaluated) in 8/10 treated animals. This was not observed in control animals. The inflammatory cellular infiltration consisted of small macrophages and lymphocytes around the sciatic nerve and involved the surface of the perineurium only. Neither the nerve fibers nor the endoneurium were involved. The inflammation involving sciatic nerve sections was considered related to local injection site responses that extended to the sciatic nerve perineurium via lymphatics and capillaries. The incidence of chronic inflammation in the perineural tissue of the sciatic nerve was reduced to 5/10 animals at SD 36, and was considered to be subsiding as a result of the healed injection sites.

In summary, under these study conditions, repeat intramuscular administration of VRC-HIVADV014-00-VP to New Zealand White rabbits resulted in a transient increase in body temperature after the first injection and a transient decrease in food consumption after each injection, which were not associated with body weight changes. Transient increases in cholesterol and triglyceride levels at SD3 were not associated with clinical symptoms or pathology and the transient increase in CPK at SD 24 was possibly related to muscle inflammation. Recoverable inflammation at the injection site and in the perineural tissue around the sciatic nerve was observed.

3.1.2 GLP biodistribution study for VRC-HIVADV014-00-VP in rabbits

Gene Logic Inc. conducted a single-dose biodistribution study of the rAd vaccine VRC-HIVADV014-00-VP in New Zealand White rabbits under Good Laboratory Practices (GLP) using intramuscular injections delivered by a needle and syringe. Animals were treated with 0.5 mL of placebo Final Formulation Buffer (FFB, VRC-DILUENT013-DIL-VP) or 0.95 x 10^{11} PU of VRC-HIVADV014-00-VP in 0.5 mL on study day (SD) 1. Five animals per sex per time point in the test article group and one animal per sex per time point in the control group were sacrificed on SD 9, SD 61 and SD 91. All tissues were shipped to Althea Technologies, Inc. (San Diego, CA) and processed for the presence of the adenoviral vector in the tissues using a GLP validated TaqMan® Polymerase Chain Reaction (PCR), developed and qualified to detect a specific target sequence in each of the four different adenoviral vectors comprising VRC-HIVADV014-00-VP. The assay detects an amplicon from each of the adenoviral vectors. The 5’- PCR primers, 3’-PCR primers and fluorescently labeled probes span regions containing the insert, polylinker and promoter. The lower limit of detection for this assay is 10 copies of the target/µg of DNA, the lower limit of quantification for the assay is 50 copies of the target/µg of DNA.

FOR COPYING ONLY

Tissues analyzed included: blood, gonads, heart, lung, liver, kidney, lymph nodes, spleen, thymus, subcutis and thigh muscle (at injection site), bone marrow (from femur on side of injection) and brain. No treatment related changes in mortality, clinical signs of toxicity, body weights or body weight changes were observed. Food consumption in the male group receiving the test article was decreased during the 24-hour period following the injection, but returned to normal after that period. The distribution profile (detailed in the table below) consisted of the VRC-HIVADV014-00-VP test article present at the injection site subcutis (5/10 animals, SD 9; 2/10 animals, SD 61) and muscle (4/10 animals, SD 9), and in the spleen (10/10 animals, SD 9; 6/10 animals, SD 61; 5/10 animals, SD 91) and liver (9/10 animals, SD 9; 2/10 animals, SD 61), with sporadic findings in the bone marrow (1/10 animals, SD 9, counts below 200 copies/µg of DNA). The number of copies of the VRC-HIVADV014-00-VP test article decreased considerably from SD 9 to SD 61 in all tissues with positive findings, and between SD 61 and SD 91 for the liver and injection site.

Table 3-1 Summary of number of rabbits with positive findings in six tissue types and average number of copies of target/µg DNA

	Marrow	Liver	Spleen	Subcutis	Muscle
**Day 9**					
Number with positive reactions	1/10	9/10	10/10	5/10	4/10
Average number of copies	23	945	1934	8088	2751
**Day 61**					
Number with positive reactions	0/10	2/10	6/10	2/10	0/10
Average number of copies	N/A	118	113	232	N/A
**Day 91**					
Number with positive reactions	0/10	0/10	5/10	0/10	0/10
Average number of copies	N/A	N/A	124	N/A	N/A

3.2 Preclinical immunogenicity studies

The Investigator Brochure provides more extensive information about the pre-clinical immunogenicity studies. There is no adequate animal model of HIV-1 infection and, as a consequence, there can be no animal studies in which to test pharmacology and protective immunization with the adenoviral vector vaccine VRC-HIVADV014-00-VP. Non-clinical immunogenicity studies were conducted with VRC-HIVADV014-00-VP in mice and non-human primates by investigators at the Vaccine Research Center (VRC), National Institutes of Allergy and Infectious Diseases, National Institutes of Health (Bethesda, MD) and Beth Israel Deaconess Medical Center, Harvard Medical School (Boston, MA). In addition, humoral immune responses were assessed in rabbits as part of the Good Laboratory Practice (GLP) preclinical toxicity studies (Gene Logic Study # 1195-114).

Several assays were used to evaluate immune responses elicited by the vaccine. Cellular immune responses were tested by the interferon gamma (IFN-γ) ELISpot assay and the flow cytometry-based intracellular cytokine staining (ICS) assay.

Humoral immune responses were measured using ELISA or a modified assay where the vaccine proteins were bound to the test plate using a lectin-capture system.

FOR COPYING ONLY

3.2.1 Immune responses to VRC-HIVADV014-00-VP vaccine in mice

A non-GLP study, "Immune responses to vaccination with VRC-HIVADV014-00-VP in mice" was conducted at the VRC. This study was designed to examine humoral and cellular immune responses. Two groups of mice were vaccinated once by intramuscular injection. In each group five mice received empty plasmid (placebo) injections and ten mice received an injection of 10^{11} PU of test article (VRC-HIVADV014-00-VP). Immune responses were tested 10 days after the injection.

HIV-1-specific cellular immune responses in vaccinated mice were demonstrated by intracellular flow cytometry. All mice also had demonstrable antibody titers (measured by ELISA) to HIV-1 proteins, following immunization with VRC-HIVADV014-00-VP.

3.2.2 Immune responses to viral gene products in rabbits

This immunogenicity study, which was part of the repeated-dose toxicology study described in protocol section 3.1.1, was conducted at Gene Logic, Inc. (Gaithersburg, MD), and the immunogenicity assessment was performed at the VRC. All test article vaccinated animals had detectable antibody titers for HIV-1 Env A, Env B, and Env C and Gag antigens in serum collected on Day 24 whereas there were no detectable antibodies in the placebo group.

3.2.3 Immunogenicity of recombinant adenoviral vector immunization of Cynomolgus macaques (VRC 03-060)

A non-GLP study in Cynomolgus macaques was conducted at Harvard Medical School and the VRC. This study was designed to investigate the magnitude and breadth of cellular immune responses elicited by recombinant adenovirus (rAd) immunizations intended for use in human trials in rhesus monkeys. The study included six outbred Cynomolgus macaques. Immunizations were administered at 10^{11} PU (delivered as two 0.5 mL injections in the quadriceps muscles) IM by needle and syringe. The macaques were bled Week 2 and Week 4 post-immunization.

ELISpot assays were utilized to monitor the emergence of vaccine-elicited T cell immune responses to multiple viral antigens. All six macaques generated responses to Clade A, B and C Env peptide pools. The study data demonstrated that the clinical rAd product is immunogenic and induces cellular immune responses against Clades A, B, C Env, as well as against Gag and Pol.

FOR COPYING ONLY

4 Clinical study

4.1 VRC Phase I study, Protocol VRC 006

The Vaccine Research Center (VRC) is conducting VRC 006 (04-I-0172), "A Phase I Clinical Trial to Evaluate the Safety and Immunogenicity of a Recombinant Multiclade HIV-1 Adenoviral Vector Vaccine, VRC-HIVADV014-00-VP, in Uninfected Adult Volunteers." This is a randomized, placebo-controlled, double-blinded, dose escalation study to examine safety, tolerability and immune response following a single injection of VRC-HIVADV014-00-VP at a dose of 10^9 PU, 10^{10} PU, or 10^{11} PU. Each group includes 12 subjects (10 vaccine; 2 placebo). VRC 006 was initiated on July 19, 2004 and the study completed enrollment of 36 subjects on November 10, 2004. The NIAID Intramural Data and Safety Monitoring Board (DSMB) reviewed the preliminary safety data through 14 days of follow-up prior to each dose escalation. The preliminary data indicate that the vaccine appears to be safe for healthy subjects at the three dose levels evaluated. The 10^9 PU and 10^{10} PU dose levels are associated with less reactogenicity than the 10^{11} PU dose level. In both the 10^9 and 10^{10} PU dose groups the local and systemic parameters recorded on the 5-day diary card were none to mild in severity and none of the subjects experienced fever. In the 10^{11} PU dose group, four subjects reported fever on Day 1 (3 mild and 1 moderate in severity). Each of the four subjects with fever also reported moderate headache on Day 1 and three of these subjects also reported at least one other moderate systemic parameter (malaise, myalgia, chills). Two subjects without fever reported at least one moderate systemic symptom (malaise, myalgia, nausea). One subject in the 10^{11} PU dose group reported moderate injection site pain; injection site reactogenicity was otherwise none or mild. There have been no serious adverse events to date. As of November 18, 2004, two events of grade 2 (moderate) severity that are possibly related to vaccination have been recorded. These are grade 2 asymptomatic neutropenia noted 21 days after study injection in a subject known to sometimes have asymptomatic low neutrophil counts and grade 2 diarrhea (duration one day) in a different subject on the third day after study injection. The safety data are expected to remain blinded until June 2005.

FOR COPYING ONLY

5 Summary

5.1 Rationale for trial design

Adenoviral vector vaccines against HIV-1 have shown good tolerability and promising immunogenicity in early human trials of a single-clade, single-transgene vector, and an adenoviral vector encoding SIV gag produced protective immune responses to SHIV challenge in a macaque model [21]. While neutralizing antibodies to adenovirus type 5 are prevalent in the human population, the effect of prior immunity on tolerability and immunogenicity of Ad5-based vaccine vectors has not been determined.

This protocol will evaluate the safety and tolerability, and will obtain preliminary data on the immunogenicity, of a multiclade, multivalent HIV-1 Ad5-based vector vaccine. Because pre-existing immunity to Ad5 may attenuate any side effects of the adenoviral vector vaccine, this protocol will study the safety of this vaccine specifically in individuals with low or absent pre-existing immunity to Ad5 (Ad5 neutralizing antibody titer <1:12). The protocol will assess vaccine safety at two escalating doses (10^{10} PU in group 1, followed by 10^{11} PU in group 2).

The results of this trial will expand the safety data available from the VRC 006 Phase I trial, which is not designed to assess safety specifically in individuals with low or absent Ad5 immunity. This safety assessment will assist in determining the appropriate vaccine dose to be used in subsequent clinical trials.

5.2 Plans for future product development and testing

In the current HVTN/VRC development plan, VRC-HIVADV014-00-VP represents the boost component of a DNA prime/adenoviral vector boost regimen. The prime consists of a mixture of DNA plasmids encoding HIV antigens analogous to those of the boost. Separate Phase I studies will investigate the safety and immunogenicity of the DNA prime alone (the ongoing VRC 004 and HVTN 052 trials), as well as the safety and immunogenicity of the combined DNA/rAd5 prime/boost strategy (VRC 010 and HVTN 057). The Phase II study, HVTN 204, will further investigate the safety and immunogenicity of a DNA/rAd5 (10^{10} PU dose) prime/boost regimen in preparation for efficacy testing.

FOR COPYING ONLY

This page is intentionally blank

FOR COPYING ONLY

STUDY DESIGN

FOR COPYING ONLY

6 Study objectives

6.1 Primary objectives

Safety

- To characterize the safety and tolerability of a single dose of the adenoviral vector vaccine delivered at each of two escalating doses (10^{10} PU and 10^{11} PU IM) in participants with low (<1:12) titers of pre-existing Ad5 neutralizing antibodies.

6.2 Secondary objectives

Immunogenicity

- To evaluate the HIV-specific immunogenicity, as assessed by IFN-γ ELISpot, ICS, ELISA for HIV-1-binding antibodies, and neutralizing antibody assays, of a single dose of the adenoviral vector vaccine delivered at each of two escalating doses (10^{10} PU and 10^{11} PU IM) in participants with low (<1:12) titers of pre-existing neutralizing antibodies to Ad5.
- Assess the magnitude of Ad5 neutralizing antibodies induced by the study vaccine.
- Assess the association between post-vaccine Ad5 neutralizing antibody titer and magnitude of HIV-1-specific immune reponses (IFN-γ ELISpot, ICS, and HIV-1 neutralizing antibodies) induced by the study vaccine.

Social impacts

- To evaluate the social impacts of trial participation.

FOR COPYING ONLY

7 Study type, study population, and eligibility criteria

The study is a Phase I multicenter, randomized, placebo-controlled, double-blind trial to evaluate the safety and immunogenicity of VRC-HIVADV014-00-VP alone. Participants will be recruited and screened; those determined to be eligible will be enrolled in the study.

Participants will receive a single vaccination at Day 0. All vaccinations will be administered by intramuscular injection in the outpatient setting.

See Table 7-1 for inclusion criteria and Table 7-2 for exclusion criteria. Final eligibility determination will depend on results of laboratory tests, medical history, physical examinations, and answers to the self-administered and/or interview questions.

See Section 12 for screening procedures.

FOR COPYING ONLY

Table 7-1 Study inclusion criteria

Note: Investigators should always use good clinical judgment in considering a volunteer's overall fitness for trial participation. Some volunteers might not be appropriate for enrollment even if they meet all inclusion /exclusion criteria because medical, psychiatric, or social conditions might make evaluation of safety and / or immunogenicity difficult.

General

Age: 18 to 50 years

Access to a participating HVTU and willingness to be followed for the planned duration of the study

Assessment of understanding: Complete a questionnaire prior to first vaccination; verbalize understanding of all questions answered incorrectly

Willingness to receive HIV test results

Informed consent: Be able and willing to provide informed consent

Health: Be in good general health as shown by medical history, physical exam, and screening laboratory tests performed within 56 days of enrollment.

Laboratory

Neutralizing antibody titers to Ad5 of <1:12

Hemoglobin: ≥ sex-specific institutional lower limit of normal

WBC count = 3,300 to 12,000 cells/mm^3.

Total lymphocyte count ≥ 800 cells/mm^3

Remaining differential either within institutional normal range or accompanied by site physician approval

Platelets = 125,000 to 550,000/mm^3

Chemistry panel

ALT, AST, alkaline phosphatase, total bilirubin, and creatinine values do not exceed institutional upper limit of normal; and CPK value does not exceed 2 times the institutional upper limit of normal

Negative HIV blood test. US participants must have a negative FDA-approved ELISA test

Negative Hepatitis B surface antigen (HBsAg)

Negative anti–Hepatitis C virus antibodies (anti-HCV), or negative HCV PCR if the anti-HCV is positive

Normal urine:

- Negative urine glucose, and
- Negative or trace urine protein, and
- Negative or trace urine hemoglobin (if trace hemoglobin is present on dipstick, a microscopic urinalysis is required to exclude participants with counts greater than the institutional normal range)

Additional inclusion criteria for female participants

Negative serum or urine β-HCG pregnancy test performed on the day of vaccination prior to vaccination

Reproductive status: a female participant must:

- agree to consistently use contraception for at least 21 days prior to enrollment until the last protocol visit, for sexual activity that could lead to pregnancy. Contraception is defined as using any of the following methods:
    - condoms (male or female) with or without a spermicide
    - diaphragm or cervical cap with spermicide
    - intrauterine device (IUD)
    - hormonal contraception
    - successful vasectomy in the male partner (considered successful if a woman reports that a male partner has [1] microscopic documentation of azoospermia, or [2] a vasectomy more than 2 years ago with no resultant pregnancy despite sexual activity post-vasectomy)
- or not be of reproductive potential, such as having reached menopause (no menses for one year) or having undergone hysterectomy, bilateral oophorectomy, or tubal ligation.
- agree not to seek pregnancy through alternative methods such as artificial insemination or in vitro fertilization until last protocol visit.

FOR COPYING ONLY

Table 7-2 Study exclusion criteria

Participant has received any of the following substances:

HIV vaccine(s) in a prior HIV vaccine trial. For potential participants who have received control/placebo in an HIV vaccine trial, documentation of the identity of the study control/placebo must be provided to the HVTN 054 Protocol Chair or Clinical Trials Physician to determine eligibility on a case-by-case basis.

Immunosuppressive medications *within 168 days before first vaccination, e.g., oral/parenteral corticosteroids, and/or cytotoxic medications. Not excluded: (1)corticosteroid nasal spray for allergic rhinitis; (2) topical corticosteroids for mild, uncomplicated dermatitis*

Blood products within 120 days before first vaccination

Immunoglobulin within 60 days before first vaccination

Live attenuated vaccines within 30 days before first vaccination

Investigational research agents within 30 days before first vaccination

Medically indicated subunit or killed vaccines, e.g., influenza within 14 days, pneumococcal within 14 days, or allergy treatment with antigen injections within 30 days prior to initial study vaccine administration

Current anti-TB prophylaxis or therapy

Participant has a clinically significant medical condition, physical examination findings, clinically significant abnormal laboratory results, or past medical history with clinically significant implications for current health. A clinically significant condition or process includes but is not limited to:

- a process that would affect the immune response
- a process that would require medication that affects the immune response
- any contraindication to repeated injections or blood draws
- a condition that requires active medical intervention or monitoring to avert grave danger to the participant's health or well-being during the study period
- a condition or process in which signs or symptoms could be confused with reactions to vaccine
- any condition specifically listed among the exclusion criteria below

Any medical, psychiatric, or social condition, or occupational or other responsibility that, in the judgment of the investigator, would interfere with, or serve as a contraindication to, protocol adherence, assessment of safety or reactogenicity, or a participant's ability to give informed consent

Serious adverse reactions to vaccines including anaphylaxis and related symptoms such as hives, respiratory difficulty, angioedema, and/or abdominal pain. *Not excluded: an adverse reaction to pertussis vaccine as a child.*

Autoimmune disease

Immunodeficiency

Active syphilis infection. *Not excluded: Syphilis fully treated over six months ago.*

Asthma *Not excluded: childhood asthma that has completely resolved*

Diabetes mellitus type I or type II, including cases controlled with diet alone. *Not excluded: Isolated gestational diabetes.*

Thyroid disease or thyroidectomy requiring medication during the last 12 months

Angioedema within the last 3 years if episodes are considered serious or have required medication within the last 2 years

Hypertension that is not well controlled by medication, or blood pressure ≥150/100 (either or both values) at enrollment.

BMI ≥ 40

Bleeding disorder diagnosed by a doctor, e.g., factor deficiency, coagulopathy, or platelet disorder requiring special precautions

Malignancy *Not excluded: A participant with a surgical excision and subsequent observation period that in the investigator's estimation has a reasonable assurance of sustained cure and/or is unlikely to recur during the period of the study*

Seizure disorder *Not excluded: A participant with a history of seizure who has not required medications or had seizure for 3 years*

Asplenia: any condition resulting in the removal of the spleen or absence of a functional spleen

Psychiatric condition that precludes compliance with the protocol. Specifically excluded are persons with any of the following:

- psychoses within the past 3 years
- ongoing risk for suicide
- history of suicide attempt or gesture within the past 3 years

Additional exclusion criteria for female participants: Pregnancy, or breast feeding, or planned to become pregnant during the period of study participation

FOR COPYING ONLY

8 Safety and immunogenicity evaluations

8.1 Initial safety evaluation

Enrollment for each group (i.e., enrollment across all participating HVTUs) will be restricted to a maximum of 1 participant per day until 5 participants have been enrolled. The HVTN 054 Protocol Safety Review Team will review the safety and reactogenicity data reported for the first 72 hours post-vaccination on each of these 5 participants and will determine whether it is safe to proceed with full enrollment in that group.

8.2 Safety considerations for dose escalation

In addition to monitoring participant safety throughout the study period, the HVTN 054 Protocol Safety Review Team (PSRT) will review cumulative safety data available on all participants in Group 1 up to and including the Day 14 visit and determine whether dose escalation may occur. The HVTN 054 PSRT may consult with the HVTN Safety Monitoring Board on an ad hoc basis for these evaluations.

8.3 Screening for adenoviral vector recombinants

Because multiple deletions render the vaccine strain replication deficient, recombination to form a replication-competent hybrid appears highly unlikely. However, because of the theoretical possibility that naturally occurring adenovirus might combine with the adenoviral vaccine vector, study participants who develop symptoms of upper respiratory infection (URI) or conjunctivitis within the four-week period following administration of the study injection will be asked to return to the study site as soon as possible within normal clinic hours for a throat swab (URI) or conjunctival swab (conjunctivitis) for adenovirus culture. If culture reveals the presence of adenovirus, a PCR analysis will be performed using the culture material to assess for the presence of vaccine vector sequences.

8.4 Distinguishing intercurrent HIV infection from vaccine-induced positive serology

The study product encodes synthetic antigen that resembles those contained in the HIV-1 virus. Therefore, vaccine-induced immune responses in study participants detected by screening serologic assays could potentially be confused with natural infection. Several precautionary measures will be taken to clarify this distinction:

- Participants will be counseled frequently during the trial on avoidance of HIV infection.
- Participants will be counseled on the risks of seeking HIV testing outside of the network during study participation, and discouraged from doing so.
- Participants will have clinical evaluations at visits specified in Appendix C. Signs or symptoms of an acute HIV infection syndrome, an intercurrent illness consistent with HIV-1 infection, or probable HIV exposure would prompt a diagnostic work-up per the standard HVTN algorithm to determine HIV infection.
    - The HVTN Laboratory Program will perform HIV-1 ELISAs from blood draws at multiple time points throughout the trial (see Appendix B).

FOR COPYING ONLY

    - If the screening visit ELISA is positive, local laboratory standards for confirmation of ELISA positive serology will be applied.
    - If positive test results are observed post-vaccination, the Laboratory Program or approved diagnostic laboratory will proceed with the HVTN algorithm to distinguish vaccine-induced antibody responses from actual HIV infection.
    - If intercurrent HIV-1 infection is suspected, further diagnostic workup will be performed per the standard HVTN algorithm to determine HIV infection.
- Continued follow-up will identify subsequent HIV infections or address concerns in participants whose HIV-1 ELISA is positive or indeterminate at the end of the study. All participants who have positive or indeterminate HIV-1 serology at the last study visit (as measured by the Abbott HIV 1, 2 kit or other standard anti-HIV antibody screening test used by blood banks) will be offered follow-up HIV-1 diagnostic testing (HIV-1 ELISA, Western blot, PCR) periodically and free of charge as medically/socially indicated (approximately every 6 months). This follow-up will be available until the ELISA/Western blot pattern no longer yields positive or indeterminate results or until HIV infection is confirmed.
- Potential participants identified as being HIV infected during screening and participants who become HIV infected during the study will be referred for medical treatment and management of the HIV infection. These individuals will also be referred to appropriate ongoing clinical trials or observation studies.

8.5 Immunogenicity evaluation

The ability of the vaccine to induce humoral responses and/or epitope-specific CD8+ and CD4+ T cell responses will be evaluated by the methods described below. For all assays, cryopreserved specimens from additional time points of immunological interest, as indicated in Appendix B, may be tested if positive responses are detected at any of the post vaccination visits.

8.5.1 Humoral immunogenicity studies

8.5.1.1 Binding antibodies by ELISA (HVTN)

Binding antibodies to commercially available Env will be assessed at the HVTN-CL by ELISA using single serum dilutions (1/50 or 1/100) on samples from all study participants taken at the baseline and at the four week post vaccination visit. Any of the time points that yield positive results, defined as an OD of ≥ 0.2, in the initial ELISA may be subject to endpoint titration ELISA employing 6 (2-7-fold) serial dilutions of serum beginning at a 1/50 or 1/100.

8.5.1.2 Neutralizing antibody assay (HVTN)

HIV-1 specific neutralizing Ab assays will be performed on serum samples from all study participants taken at baseline and at the four week post vaccination visit (see Appendix B). The assays will test neutralization of HIV-1 MN and the HIV-1 strains represented in the vaccine constructs (HXB2, Bal, 92rw020, and 97ZA012). The serum samples with the highest antibody response (as judged by either endpoint ELISA titer >100 or neutralization) may be further assayed against a panel of heterologous primary isolates and analyzed separately.

FOR COPYING ONLY

As an exploratory assay, the HVTN may examine neutralizing antibody responses against a panel of heterologous primary isolates using cross-sectional serum samples. The assays will be performed in a subset of participant samples with the highest antibody responses. This subset will include the 12 highest responders, as judged by endpoint ELISA titer >100, plus 3 placebo recipients.

8.5.2 Cellular immunogenicity studies

8.5.2.1 IFN-γ ELISpot (HVTN)

Bulk T cell responses will be assessed by IFN-γ ELISpot using cryopreserved peripheral blood mononuclear cells (PBMC) stimulated overnight with synthetic peptide pools that span the proteins encoded by the vaccine constructs. ELISpot assays will be performed at the baseline and at the four week post vaccination visit (see Appendix B). Responses will be reported as the number of spot forming cells (SFC) per 10^6 cells/well recognizing any specific peptide pool.

8.5.2.2 Intracellular cytokine staining (HVTN and VRC)

HVTN

Flow cytometry will be used to examine HIV-specific CD4+ and CD8+ T cell responses using intracellular cytokine staining (ICS) following stimulation with synthetic HIV peptides that span the proteins encoded by the vaccine construct. ICS assays will be performed at the baseline and at the four week post vaccination visit (see Appendix B). Responses will be reported as percentages of CD4+ or CD8+ T cells recognizing any specific peptide pool.

VRC

A flow cytometric assay for the detection of ICS will be performed at the VRC Lab after stimulation with synthetic peptides that span the specific multiclade HIV-1 proteins encoded by the vaccine construct at several time points of immunogical interest (see Appendix B). Responses will be reported as percentages of T cells (CD4+, CD8+, both) recognizing any specific peptide pool.

8.6 HLA typing

Molecular HLA typing may be performed on enrolled participants using cryopreserved PBMC at baseline initially in participants who demonstrate vaccine-induced T-cell responses at post-immunization time points. Other participants (including placebo recipients) may be HLA-typed to support future studies of immunological interest at the discretion of the protocol chair and the Laboratory Program. These assays may include, but are not limited to, fine epitope mapping by flow cytometry or ELISpot, or flow cytometric tetramer analysis.

8.7 Ancillary studies

Cryopreserved samples may be used to perform additional ELISpot and ICS assays to support the standardization and validation of these assays, and in other immunological assays of interest to guide future research. These assays may include, but are not limited to, fine epitope mapping by flow cytometery or ELISpot, or flow cytometric tetramer analysis.

Neutralizing antibody against viral vector (Ad5)

The VRC laboratory will evaluate pre-existing adenovirus neutralizing antibodies at screening and neutralizing antibodies produced against the adenoviral vector at four weeks

FOR COPYING ONLY

post vaccination. The VRC laboratory may also perform humoral assays at other time points of immunological interest, including ELISA and neutralizing antibody assays to both HIV-1 and adenovirus.

FOR COPYING ONLY

9 Statistical considerations

9.1 Overview

This study is a multicenter, randomized, placebo-controlled, double-blind trial. The data analysis will evaluate safety and immunogenicity of the multiclade, multivalent HIV-1 vaccine at each of 2 escalating doses (10^{10} PU and 10^{11} PU) in participants with low (<1:12) titers of pre-existing Ad5 neutralizing antibodies at continental US sites.

9.2 Objectives

The primary objectives of this trial concern safety; the secondary objectives concern immunogenicity and social impacts. See Section 6.1 and Section 6.2 for details.

9.3 Endpoints

9.3.1 Safety

Assessment of product safety will include clinical observation and monitoring of hematological and chemical parameters. Safety will be evaluated by monitoring participants for local and systemic adverse reactions for 12 months after injection.

The following parameters will be assessed:

- Local reactogenicity signs and symptoms
- Systemic reactogenicity signs and symptoms
- Laboratory measures of safety
- Adverse and serious adverse experiences

9.3.2 Immunogenicity

Immunogenicity is a secondary objective with the following endpoints:

- Unfractionated IFN-γ ELISpot responses to HIV-1 at the 4-week-post-vaccination visit
- CD4+ and CD8+ T-cell responses to HIV-1 as measured by flow cytometry-based intracellular cytokine staining (ICS) assays at the 4-week-post-vaccination visit
- HIV-1 ELISA binding antibodies at the 4-week-post-vaccination visit
- HIV-1 neutralizing antibodies at the 4-week-post-vaccination visit
- Ad5 neutralizing antibodies at the 4-week-post-vaccination visit

9.3.3 Social impacts

Social impact variables are secondary endpoints and include any positive or negative experiences or problems the participant experienced due to participation in this study. The following social impacts will be followed during the course of the study: social, travel, work, school, health care, life insurance, health insurance, housing, military and any additional impacts identified by a participant.

FOR COPYING ONLY

9.4 Accrual and sample size

Recruitment will target 48 healthy, HIV-uninfected adult participants with screening neutralizing antibody titers to Ad5 less than 1:12. Twenty-four participants will be enrolled in each of the two dose groups and randomized to vaccine or control in a 5:1 ratio. Enrollment in Group 2 can begin once the dose escalation criteria are met for all participants in Group 1. See Section 14.7.5 for details.

Since enrollment is concurrent with receiving the study vaccination, all participants will provide some safety data hence sample size calculations for safety in Section 9.4.1 are based on the target sample sizes.

9.4.1 Sample size calculations for safety

The goal of the safety evaluation for this study is to identify safety concerns associated with injection. Sample size calculations for safety are expressed in terms of the ability to detect safety events (e.g., serious adverse experiences).

The sensitivity of the study to identify serious adverse experiences is best expressed by the maximum true rate of events that would be unlikely to be observed and the minimum true rate of events that would very likely be observed. Specifically, for each vaccine arm of the study (n=20), there is a 90% chance of observing at least one serious adverse experience if the true rate of such an event is at least 11%; there is a 90% chance that we would not observe at least one serious adverse experience if the true rate was no more than 0.5%. Probabilities of observing zero or two or more serious adverse experiences among groups of size 20 are presented in Table 9-1 for a range of possible true event rates. These calculations provide a more complete picture of the sensitivity of this study design to identify potential safety problems with the vaccine.

If none of the 40 participants receiving the vaccine experience severe adverse experiences to the vaccine, the 95% two-sided exact upper confidence bound for the rate of such reactions in the population is 9%. Restricted to any of the vaccine arms (n=20), the exact two-sided upper confidence bound for this rate is 17%.

Table 9-1 Probability (Pr) of response for different safety and immunogenicity scenarios

True event rate	Pr(0/20)	Pr(1+/20)	Pr(2+/20)
3.5%	0.49	0.51	0.15
5%	0.36	0.64	0.26
10%	0.12	0.88	0.61

9.4.2 Sample size calculations for immunogenicity

The primary goal regarding immunogenicity is a preliminary estimation of response rates and does not involve formal comparison of these rates between vaccine arms. The precision with which response rates can be estimated, based on a sample of 20 vaccinees, is limited. The standard error of the estimated response rates depends on the true underlying response rate but can be bounded by 0.11 ($=\sqrt{0.5 \times 0.5 / 20}$). Thus, the width of a 95% confidence interval for the response rate in any one arm will be no greater than 0.44 (i.e. ±1.96 x 0.11).

Since individual groups are relatively small, the power to detect significant differences in response rates between placebo and vaccine recipients at a specific time point is low. For example, as in the safety power calculations, a difference between a 3.5% T cell response rate

FOR COPYING ONLY

in the combined placebo arms (n=8) and a 50.5% response rate in the combined vaccine arms (n=40) is detectable with 80% power for a two-sided exact test with Type I error rate of 0.05. However, the probability of observing at least one response among vaccinees in a treatment group is adequate. For example, if the T cell response rate at a particular time point is 10%, then there is a 0.88 probability of observing at least one positive response in a vaccine arm with 20 vaccinees.

9.5 Statistical analysis

All data from enrolled participants will be analyzed according to the initial randomization assignment. Since enrollment is concurrent with receiving the study vaccination, all participants will have received the vaccination and therefore will provide some safety data. The analysis is intent-to-treat; however, individuals who are randomized but not enrolled do not contribute data and hence are excluded. Because of blinding and the brief length of time between randomization and enrollment—typically within 4 working days, according to the *HVTN Manual of Operations* (HVTN MOP) (Study Operations >Enrollment >Randomization)—very few such individuals are expected.

All descriptive and inferential statistical analyses will be performed using SAS and S-Plus statistical software. When the use of descriptive statistics to assess group characteristics is required, the following statistics will be reported: for categorical variables, the number and percent in each category; for continuous variables, the mean, median, standard deviation, quartiles, and range (minimum, maximum).

No formal multiple comparison adjustments will be employed for safety endpoints, primary immunogenicity endpoints that address separate scientific questions (e.g., humoral- and cellular-based endpoints), or secondary endpoints. Multiple measurements of a specific type of immune response will be treated as a collection of hypotheses that requires a multiplicity adjustment. For example, determination of cellular immune responses to several different HIV-1 peptide pools as measured by the IFN-γ ELISpot assay will entail a multiplicity adjustment to account for the multiple peptide pools considered.

9.5.1 Analysis variables

The analysis variables consist of baseline variables, safety variables, immunogenicity variables, and social impact variables for primary and secondary objective analyses.

9.5.2 Baseline comparability

Groups will be compared for baseline characteristics including demographics and laboratory measurements, using descriptive statistics.

9.5.3 Safety analysis

Reactogenicity

The number and percentage of participants experiencing each type of reactogenicity sign or symptom will be tabulated by treatment. For a given sign or symptom, each participant's reactogenicity will be counted once under the maximum severity for all injection visits.

Adverse experiences

Adverse experiences are coded into MedDRA preferred terms. The number and percentages of participants experiencing each specific adverse event will be tabulated by severity and

FOR COPYING ONLY

relationship to treatment. For the calculations in these tables, each participant's adverse experience will be counted once under the maximum severity or strongest recorded causal relationship to treatment.

A complete listing of serious adverse experiences for each participant will provide details including severity, relationship to treatment, onset, duration and outcome.

Local laboratory values

Boxplots of local laboratory values by treatment will be generated for baseline values and for values measured during the course of the study. Each boxplot will show the first quartile, the median, and the third quartile. Outliers or values outside the boxplot will also be plotted. Horizontal lines representing boundaries for abnormal values will be plotted, if applicable.

9.5.4 Immunogenicity analysis

The statistical analysis for immunogenicity will employ the intent-to-treat principle whereby all data from enrolled participants will be used according to the initial randomization assignment. The only exception will be to exclude data from HIV-infected participants at or post infection. If the HIV positivity status of an infected participant is unknown at the time that the first sample for immunogenicity assessments is drawn, then all data from that participant will be excluded from the analysis.

If assay data are qualitative (i.e., positive or negative), then analyses will be performed by tabulating the frequency of positive response for each assay by dose group at each time point that an assessment is performed. All responses mentioned here are crude responses; there will be insufficient data to adjust for false positive responses from the placebo arms. Binomial response rates will be presented with their corresponding exact 95% confidence interval estimates. Significant differences between groups will be determined by a two-sided Fisher's exact test. In addition to reporting the point prevalence response rates, cumulative and cumulative repeat probabilities of cellular immune responses will be estimated with corresponding confidence intervals using maximum likelihood based methods [74]. Missing responses will be assumed to be missing at random, i.e., conditional on the observed data the missingness is independent of the unobserved responses.

For continuous assay variables, overall differences between groups at a specific time point will be tested by a two-sample t-test if the data appear to be normally distributed. If not, the nonparametric Wilcoxon rank sum test will be used. If a portion of the measurements are censored below the assay quantification limit, the Gehan-Wilcoxon test will be employed. More sophisticated analyses employing repeated measures methodology (for example, repeated measures ANOVA or generalized estimating equations) may be used to incorporate immune responses over several time points. All statistical tests will be two-sided and will be considered statistically significant if $p \leq 0.05$. Graphical descriptions of the longitudinal immune responses will also be given.

Some immunologic assays have underlying continuous or count-type readout that is often dichotomized into responder/nonresponder categories. For these assays, graphical and tabular summaries of the underlying distributions will be made. These summaries may be performed on transformed data (e.g., log transformation) to better satisfy assumptions of symmetry and homoscedasticity. If group comparisons in these underlying distributions reveal that differences are best summarized as a shift in the location of the distribution, then results will be presented in the form of group means (or medians) with associated confidence intervals and statistical tests for differences between groups as described above. If group comparisons in these underlying distributions reveal that differences are best summarized by a mixture

FOR COPYING ONLY

model (i.e., responder and nonresponder subgroups are clearly identifiable) then results will be presented in the form of response rates with associated confidence intervals and statistical tests as described above.

Missing data

If the probability of missing immunogenicity measurements depends on either covariates or on the immunogenicity outcomes of participants, then the methods described above may give biased inferences and point estimates. If a substantial amount of immunogenicity data are missing (at least 1 value missing from more than 20% of participants), then secondary analyses of the immunogenicity endpoints will be conducted using methods that relax the missing completely at random assumption to a missing at random assumption. For a univariate binary and quantitative outcome, respectively, a generalized linear model with a binomial or normal error distribution will be used for estimation and testing. For assessing repeated immunogenicity measurements, linear mixed effects models will be used. The models will be fit using maximum likelihood methods, and will include as covariates all available baseline predictors of the missing outcomes. The longitudinal models will also include all observed immunogenicity data.

9.5.5 Social impact analysis

Social impacts will be tabulated by type of event and impact on quality of life. The number and percentage of participants experiencing each type of social impact will also be tabulated by impact on quality of life. For this calculation multiple events of the same type for a participant will be counted once under the maximum impact for all post-vaccination visits.

In addition, for each type of social impact a longitudinal listing will be generated for all participants who experienced a major disturbance starting with the first major disturbance followed by all subsequent impacts. Included in this listing will be descriptions of the impact, duration, impact on quality of life, actions taken by the participant and staff, and whether or not there was a resolution.

9.5.6 Interim analyses

Unblinded interim analyses of safety data are prepared at regular intervals for the HVTN Safety Monitoring Board (SMB).

No formal interim analyses are planned.

9.6 Randomization of treatment assignments

The randomization sequence will be obtained by computer-generated random numbers and provided to each HVTU by the SDMC using the procedures described in the HVTN MOP (Study Operations>Enrollment>Randomization). At each institution, the pharmacist with primary responsibility for drug dispensing is charged with maintaining security of the randomization code. Randomization will be done sequentially for each dose group and in blocks within the dose groups to ensure balance across groups.

FOR COPYING ONLY

This page is intentionally blank

FOR COPYING ONLY

STUDY OPERATIONS

FOR COPYING ONLY

10 Protocol conduct

The protocol will be conducted according to standard HVTN policies and procedures specified in the HVTN MOP (Study Operations), including procedures for the following:

- Protocol registration, activation and implementation
- Informed consent, screening, enrollment
- Clinical and safety assessments
- Safety monitoring and reporting
- Data collection and documentation
- Study follow-up and close-out
- Unblinding of staff and participants
- Quality control
- Protocol monitoring and compliance
- Advocacy and assistance through local and governmental activities to participants regarding social harms associated with the vaccine trial
- Risk reduction counseling
- Outside testing and belief questionnaire

Any policies or procedures that vary from HVTN standards or require additional instructions will be described in the *HVTN 054 Study Specific Procedures* (e.g., instructions for randomization specific to this study).

FOR COPYING ONLY

11 Informed consent

Informed consent is the essential process of ensuring that participants fully understand what will and may happen to them while participating in a research study. The HVTN informed consent form documents that a participant (1) understands the potential risks, benefits, and alternatives to participation, and (2) is willing to participate in an HVTN study. Informed consent is not confined to the signing of the consent form; it also includes all written or verbal study information HVTU staff discuss with the participant, before and during the trial. HVTU staff will obtain informed consent of participants according to the HVTN policies and procedures specified in the HVTN MOP (Study Operations>Informed Consent).

An HVTU may employ recruitment efforts prior to the participant consenting. But before any protocol-specific questions are asked or procedures to determine protocol eligibility performed, the screening consent form or protocol-specific consent form (described below) must be signed. Participants will be provided with a copy of all consent forms that they sign.

11.1 Screening consent form

Some HVTUs have approval from their local Institutional Review Board (IRB) and/or Independent Ethics Committee (IEC) to use a general screening consent form that allows screening for an unspecified HIV vaccine trial. In this way, HVTU staff can continually screen potential participants, and when needed, proceed quickly to obtain protocol-specific enrollment consent. Sites conducting IRB/IEC–approved general screening or pre-screening may use the results from this screening for determining eligibility in this protocol, provided the tests are conducted within the time period specified in the eligibility criteria.

11.2 Protocol-specific consent form

The protocol-specific consent form describes the study products to be used and all aspects of protocol participation, including screening and enrollment procedures. A sample protocol-specific consent form is located in Appendix A.

Each HVTU is responsible for developing a protocol-specific consent form for local use, based on the sample protocol-specific consent form in Appendix A. The consent form must be developed in accordance with local IRB/IEC requirements and the principles of informed consent as described in Title 45, Code of Federal Regulations (CFR) Part 46 and Title 21 CFR, Part 50, and in the International Conference on Harmonisation (ICH) Guideline 4.8.10. It must be approved by all responsible ethical review bodies before any participants are consented for the study.

The sample form in Appendix A includes interspersed instructions for developing specific content.

11.3 Assessment of understanding

Study staff should ensure that participants fully understand the study before enrolling them. This involves reviewing the informed consent form with the participant, allowing time to reflect on the procedures and issues presented, and answering all questions completely.

An Assessment of Understanding is used to document the participant's understanding of key concepts in an HIV vaccine trial. Completion of the Assessment of Understanding is an inclusion criterion for the trial.

FOR COPYING ONLY

The Assessment of Understanding should not be administered until the participant has signed a consent form. This can be a screening consent form, if the HVTU uses one. If not, the participant must sign the protocol-specific consent prior to administration of the Assessment of Understanding.

The participant must complete the Assessment of Understanding before vaccination, and verbalize understanding of all questions answered incorrectly. This process, and the participant's understanding of the key concepts, should be documented in source documentation at the site.

FOR COPYING ONLY

12 Procedures

Participants are considered to be enrolled only upon receipt of the study vaccination at Day 0.

HVTU and Central Lab staff will conduct screening and post-enrollment study procedures according to HVTN procedures as specified in the HVTN MOP (Study Operations). Any procedures which vary from the HVTN standard will be defined in the *HVTN 054 Study Specific Procedures*.

Pre-enrollment and post-enrollment procedures are performed on all participants (unless otherwise noted) at the time points indicated in Appendices B and C, using the blood draw volumes specified.

12.1 Pre-enrollment procedures

An HVTU may employ recruitment efforts prior to the participant consenting. But before any protocol-specific questions are asked or procedures to determine protocol eligibility performed, the screening consent form or protocol-specific consent form (see Section 11) must be signed. Participants will be provided with a copy of all consent forms that they sign.

Screening assessments and other pre-enrollment procedures are listed in Table 12-1. Time points are specified in Appendices B and C.

Screening procedures are done to determine eligibility and to provide a baseline for comparison of safety data. Screening may occur over the course of several contacts/visits up to and including Day 0 before vaccination. All inclusion and exclusion criteria must be assessed within 56 days before enrollment, unless otherwise specified in Table 7-1 and Table 7-2.

The time interval between randomization and enrollment should not exceed 4 working days, as defined in the HVTN MOP (Study Operations). Subsequently, the HVTU registers the participant by scheduling the Day 0 visit (enrollment) via the web-based randomization system, and requests the randomization assignment.

12.2 Post-enrollment procedures

Safety assessments, immunogenicity determinations, and other post-enrollment procedures are listed in Table 12-2.

FOR COPYING ONLY

Table 12-1 Pre-enrollment procedures

Screening assessments			
Clinical assessments	Local lab assessments		HIV infection assessments
Medical history	Pregnancy test (females)	Chemistry panel:	HIV ELISA
Complete physical exam	Urine dipstick/urinalysis	ALT	
Concomitant medications	CBC with differential	AST	
Abbreviated physical exam	Platelet count	Alkaline phosphatase	
	Syphilis (RPR)	Total bilirubin	
	Hepatitis B	Creatinine	
	Hepatitis C	CPK	

Other pre-enrollment procedures	
Screening informed consent (if applicable)	Behavioral risk assessment
Protocol informed consent	Risk reduction counseling
Assessment of understanding	Pregnancy prevention counseling
Specimen collection	HIV pre- and post-test counseling
Obtain demographics	Participant randomization
Confirm eligibility	Adenovirus neutralizing antibodies

Table 12-2 Post-enrollment procedures

Safety assessments			
Clinical assessments	Local lab assessments	Other assessment	HIV infection assessments
Abbreviated physical exam	Urine dipstick/urinalysis	Adenovirus PCR (if applicable)	HIV ELISA
Complete physical exam	CBC with differential		HIV Western blot (if applicable)
Concomitant medications	Platelet count		HIV RNA PCR
Intercurrent illness/AE	Pregnancy test (females)		HIV DNA PCR
Reactogenicity	Adenovirus culture (if applicable)		
	Chemistry panel (see Table 12-1)		

Immunogenicity determinations		
HVTN humoral assays	HVTN cellular assays	VRC assays
HIV-1 neutralizing antibodies	IFN-γ ELISpot	Humoral studies
HIV-1 binding ELISA	ICS	Adenovirus neutralizing antibodies
		ICS

Other post-enrollment procedures		
Specimen collection and shipping	Pregnancy prevention counseling	Cryopreservation/storage of specimens
HIV pre- and post-test counseling	Social impact assessment	HLA typing
Risk reduction counseling	Outside testing/belief assessment	Participant unblinding

FOR COPYING ONLY

12.3 Total blood volumes

Required blood volumes are shown in Appendix B. Not shown is any additional blood volume that would be required if a safety lab needs to be repeated, or if a serum pregnancy test needs to be performed. The additional blood volume would likely be minimal. The total blood volume drawn for each participant will not exceed 500 mL in any 8-week period.

12.4 Laboratory procedures

A laboratory procedures manual will be available that provides further guidelines for operational issues concerning the clinical laboratories and phlebotomy. The procedures include general specimen collection guidelines, special considerations for blood collection, HIV testing guidelines, suggested tube types with catalog numbers, guidelines for processing whole blood, and labeling guidelines.

In specific situations the blood collection tubes will be redirected to another laboratory for special screening criteria or safety issues. In these cases special shipping instructions will be provided in Special Instructions posted on the HVTN website.

FOR COPYING ONLY

13 Study product preparation and administration

HVTU pharmacists should consult the *Pharmacy Guidelines and Instructions for DAIDS Clinical Trials Networks* manual for standard pharmacy operations procedures. The protocol schema and vaccine regimen are shown in Section 13.1. See the Investigator's Brochure for further information about study products.

13.1 Schema and vaccine regimen

Vaccine: VRC-HIVADV014-00-VP (Clade B Gag and Pol, Clades A, B, C Env; rAD)

Placebo: VRC-DILUENT013-DIL-VP

Administration: one 1 mL injection in either deltoid

Study arm	Treatment	Injection schedule in months (days)<br>0 (0)
Group 1	T1	VRC-HIVADV014-00-VP 1x10^{10} PU IM
	C1	Placebo 1 mL IM
Group 2	T2	VRC-HIVADV014-00-VP 1x10^{11} PU IM
	C2	Placebo 1 mL IM

Groups 1 and 2 will be enrolled sequentially (see Section 8.2).

Group 1

T1: VRC-HIVADV014-00-VP 1x10^{10} PU IM administered as a 1 mL injection in either deltoid on Day 0

C1: Placebo (VRC-DILUENT013-DIL-VP) 1 mL IM in either deltoid on Day 0

Group 2

T2: VRC-HIVADV014-00-VP 1x10^{11} PU IM administered as a 1 mL injection in either deltoid on Day 0

C2: Placebo (VRC-DILUENT013-DIL-VP) 1 mL IM in either deltoid on Day 0

13.2 Study product formulation and preparation

See the Investigator's Brochure for further information about study products.

13.2.1 VRC-HIVADV014-00-VP (Multiclade HIV-1 Recombinant Adenoviral Vector Vaccine, rAD)

VRC-HIVADV014-00-VP is manufactured by GenVec Incorporated at a contract manufacturer, Molecular Medicine. The vaccine is supplied as two different concentrations (1x10^{10} or 1x10^{11} PU). It is provided in 3 mL sterile glass vials containing 1.2 mL of a clear colorless isotonic sterile solution. The study agent must be stored frozen (-30°C to -10°C) until use. (Please note that although the product is labeled for storage at -25°C to -10°C, the Investigator's Brochure allows for temperatures as low as -30°C).

FOR COPYING ONLY

13.2.2 VRC-DILUENT013-DIL-VP (Final Formulation Buffer, FFB, Placebo)

VRC-DILUENT013-DIL-VP Final Formulation Buffer (FFB) is manufactured at BioWhittaker and will be used as the placebo in all groups. It is composed of sodium chloride, Tris buffer, trehalose•$2H_20$ (low endotoxin), magnesium chloride•$6H_20$, mono-oleate (Tween 80) and water for injection (WFI). The study agent is supplied in 3 mL vials containing 1.2 mL of a clear, colorless, isotonic solution with a pH of 7.8 ± 0.2 and should be refrigerated at 2-8°C.

13.2.3 Study product preparation

For all groups: Any unused portion of used vials and expired pre-filled syringes should be disposed of in a biohazard container and incinerated or autoclaved.

Group 1

Remove one vial of VRC-HIVADV014-00-VP $1x10^{10}$ PU/mL from the freezer or one vial of the VRC-DILUENT013-DIL-VP (placebo) from the refrigerator and allow to equilibrate to room temperature. Once thawed, the vial should not be refrozen. Using aseptic technique, withdraw 1 mL of the study product into a syringe. The study agent must be administered within 4 hours of removal from the freezer/refrigerator.

Group 2

Remove one vial of VRC-HIVADV014-00-VP $1x10^{11}$ PU/mL from the freezer or one vial of the VRC-DILUENT013-DIL-VP (placebo) from the refrigerator and allow to equilibrate to room temperature. Once thawed, the vial should not be refrozen. Using aseptic technique, withdraw 1 mL of the study product into a syringe. The study agent must be administered within 4 hours of removal from the freezer/refrigerator.

13.2.4 Labeling procedures to preserve blinding

The pharmacist will prepare all doses for administration and dispense to the clinic. All syringes must be labeled with the time the study agent was removed from the freezer/refrigerator. In addition, the syringes must be labeled with an expiration time that is 4 hours after this “removal” time. To preserve blinding, the pharmacist will place a yellow overlay on the syringes.

13.3 Study product administration

All injections are to be given intramuscularly into either deltoid muscle as a volume of 1 mL using a 21 gauge needle with a length of 1 or 1-1/2 inch (depending on subject arm size).

When preparing a dose in a syringe and administering the dose, consideration should be given to the volume of solution that may remain in the needle after the dose is administered. The pharmacy and clinic staff are encouraged to work together to administer the dose specified in the protocol. At sites where registered pharmacists are legally authorized to administer drug, the HVTU may choose to have the HVTU pharmacist administer the vaccinations.

13.4 Study product acquisition

Study products will be provided by the VRC. The HVTU pharmacist can obtain study products from the NIAID Clinical Research Products Management Center (CRPMC) by following the ordering procedures given in the section on Investigational Drug Control in *Pharmacy Guidelines and Instructions for DAIDS Clinical Trials Networks*.

FOR COPYING ONLY

13.5 Pharmacy records

The HVTU pharmacist is required to maintain complete records of all study products received from the CRPMC and subsequently dispensed. All unused study products must be returned to the CRPMC after the study is completed or terminated unless otherwise instructed by the CRPMC. The procedures are included in the sections on Investigational Drug Control and Drug Dispensing in *Pharmacy Guidelines and Instructions for DAIDS Clinical Trials Networks*.

The pharmacist of record is responsible for maintaining randomization codes and randomization confirmation notices for each participant in a secure manner.

FOR COPYING ONLY

14 Safety monitoring and review

14.1 Assessing reactogenicity

Reactogenicity assessments are performed for all participants following each vaccination. HVTU staff will assess reactogenicity according to standard HVTN procedures as specified in the HVTN MOP (Study Operations>Safety Assessment>Reactogenicity). Any procedures which vary from the HVTN standard will be defined in *HVTN 054 Study Specific Procedures*.

The reactogenicity assessment period is for 3 days following the vaccination. Participants are instructed to record symptoms using a post-vaccination symptom log and contact the site daily during this reactogenicity assessment period. Clinic staff will follow new or unresolved reactogenicity symptoms present at Day 3 to resolution. The schedule is shown in Table 14-1.

Assessments to be performed:

- Systemic symptoms: body temperature, malaise and/or fatigue, myalgia, headache, chills, arthralgia, nausea, vomiting
- Local symptoms (proximal to injection site): pain, tenderness
- Vaccine-related lesions: erythema, induration
- Axillary lymph nodes (required only when reactogenicity assessments are performed by HVTU staff): lymph node tenderness, enlargement

Table 14-1 Schedule of reactogenicity assessments

Day	Time	Performed by
0[a]	Baseline: before vaccination	HVTU staff
	Early: 25 to 45 minutes after vaccination	HVTU staff
	Between early assessment and 11:59pm Day 0	HVTU staff or participant
1	Between 12:00am and 11:59pm Day 1	HVTU staff or participant
2	Between 12:00am and 11:59pm Day 2	HVTU staff or participant
3[b]	Between 12:00am and 11:59pm Day 3	HVTU staff or participant

[a]Day of vaccination
[b]New or unresolved reactogenicity symptoms present on Day 3 are followed until resolution

14.2 Grading of adverse experiences

Assessments of local and systemic symptoms are graded based on the *HVTN Table for Grading Severity of Adverse Experiences* (revised September 2002-updated version).

14.3 Adenovirus culture

Study participants who develop symptoms of upper respiratory infection (URI) or conjunctivitis within the four-week period following administration of the study injection will be asked to return to the study site as soon as possible within normal clinic hours for a throat swab (URI) or conjunctival swab (conjunctivitis) for adenovirus culture. If culture reveals the presence of adenovirus, a PCR analysis will be performed using culture material to assess for the presence of vaccine vector sequences.

FOR COPYING ONLY

14.4 Adverse experience reporting and safety pause

All adverse experiences are reported to the SDMC on the appropriate case report form (CRF) according to procedures in the HVTN MOP (Study Operations>Safety Assessments>Adverse Experiences). The mechanism of reporting vaccine-related symptoms and adverse experiences to the SDMC clinical affairs staff, and the cumulative events triggering a safety pause, are depicted in Table 14-2.

Adverse experience(s) triggering a safety pause are vaccine related. 'Vaccine related' means the event is judged to be possibly related, probably related, or definitely related to the study vaccination.

If a safety pause is triggered, SDMC clinical affairs staff notifies the HVTN 054 Protocol Safety Review Team (PSRT), DAIDS Pharmaceutical Affairs Branch (PAB), Regulatory Compliance Center (RCC), Regulatory Affairs Branch (RAB), and participating HVTUs that vaccinations are held until further notice. In addition, in the case of a safety pause that can be triggered by only 1 event (any Grade 4 event, any Grade 3 event except subjective local and systemic symptoms, Grade 2 erythema or induration), SDMC clinical affairs staff notifies the HVTN SMB; DAIDS notifies the US FDA.

Each HVTU is responsible for submitting to its IRB/IEC protocol-related safety information (such as IND safety reports, notifications of vaccine holds due to safety pause rules, etc.) as required by the local institution.

14.4.1 Follow up and resolution after a safety pause

The HVTN 054 Protocol Safety Review Team reviews safety data and decides whether permanent discontinuation of vaccination is appropriate, consulting the HVTN SMB and the US FDA if necessary. DAIDS notifies the US FDA of the decision. SDMC clinical affairs staff notifies participating HVTUs, PAB, and RCC/RAB of the decision.

14.4.2 Reporting serious adverse experiences to RCC and SCHARP

HVTUs must report adverse experiences that meet Serious Adverse Experience (SAE) reporting criteria and time frames set forth in the *DAIDS Serious Adverse Experience Reporting Manual for HVTN* (November 15, 2000; updated contact information and SAE Form, 6 October 2003) on the SAE Report Form and submit the form to DAIDS through the Regulatory Compliance Center (RCC). The HVTU must simultaneously send a copy of the SAE Report Form to Clinical Affairs at SCHARP.

The RCC will forward SAE information to DAIDS. HVTU staff may be contacted by the RCC for additional information needed for follow-up and IND safety reports.

FOR COPYING ONLY

Table 14-2 Adverse experience notification and safety pause rules

Toxicity	Symptom/AE	HVTU action	Criterion for pause
Grade 4	Any lab abnormality, local or systemic symptom, adverse experience	Immediate	≥1 of the same vaccine-related symptom at specified grade
Grade 3	Lab abnormality, fever, vomiting, other clinical AE (except subjective local and systemic symptoms)	Immediate	≥1 of the same vaccine-related symptom at specified grade
	Subjective local and systemic symptoms: pain, tenderness, malaise and/or fatigue, headache, chills, nausea, myalgia, arthralgia	Prompt	≥2 ppts report the same vaccine-related symptom at specified grade or higher
Grade 2 or 3	Erythema, induration	Prompt	≥1 of the same vaccine-related symptom at specified grade
Grade 2	Lab abnormality, fever, vomiting, other clinical AE (except subjective local and systemic symptoms)	Prompt	≥2 ppts experience the same vaccine-related symptom at specified grade or higher

Immediate: HVTU notifies SDMC clinical affairs staff immediately by pager or live phone (not voice mail)

Prompt: HVTU notifies SDMC clinical affairs staff by pager or live phone (not voice mail) within 1 working day of receiving notice of event

Phone number and pager number are listed in *HVTN 054 Study Specific Procedures*

14.5 Participant termination from the study

Under certain circumstances, an individual participant may be terminated from participation in this study. Specific events that will result in early termination include:

- Participant refused to participate further
- Participant relocated to an area without a nearby HVTU and remote follow-up is not possible
- HVTU determined that the participant is lost to follow-up
- Participant becomes HIV-infected

14.6 Study termination (for all participants)

This study may be terminated by the determination of the HVTN 054 Protocol Safety Review Team, HVTN Safety Monitoring Board, US FDA, US NIH, vaccine developer, or regulatory authority (e.g., IRB or IEC). See Section 14.7 for discussion of the safety review process.

14.7 HVTN review of cumulative safety data

Routine safety review occurs at the start of enrollment, and then daily, weekly, monthly and quarterly during the study.

Reviews proceed from a standardized set of protocol-specific safety data reports. These reports are produced by SDMC and annotated with queries to the HVTU and additional notes. Events are tracked by the internal reports until resolution. Other reports, containing the queries and notes, are distributed to the HVTN 054 Protocol Safety Review Team. The following reports are produced:

- Clinical quality control

FOR COPYING ONLY

- Safety review
- Pre-existing conditions
- Adverse events (AEs) requiring review
- Adverse event/concomitant medication
- WBC/differential
- Safety summary

More detailed information regarding the contents and distribution of these reports can be found in the HVTN MOP.

14.7.1 Daily review

Blinded daily safety reviews are routinely conducted by the SDMC clinical affairs staff for SAEs and events that meet safety pause criteria (Table 14-2).

14.7.2 Weekly review

Blinded weekly safety reviews are routinely performed by the SDMC clinical affairs staff and by the HVTN 054 Protocol Safety Review Team. The SDMC clinical affairs staff reviews reports of all clinical values that fall outside of the standard HVTN safety parameters (see HVTN MOP [Study Operations>Standard Reports>Clinical Safety Review>Weekly Safety Review Reports]). Values identified during the review that are considered questionable, inconsistent, or unexplained are referred to the HVTU clinic coordinator for verification.

The HVTN 054 Protocol Safety Review Team is composed of the following required members:

- Protocol chair, co-chair
- HVTN clinical trials physician
- SDMC clinical affairs staff member
- DAIDS medical officer

The protocol team clinic coordinator, protocol specialist, and vaccine developer representative may also be included at the request of the HVTN 054 PSRT.

Weekly during the vaccination phase of the trial, the HVTN 054 PSRT reviews an annotated version of the appropriate clinical safety reports containing queries to the HVTU. After the vaccinations and the final 2-week safety visits are completed, less frequent safety reviews may be scheduled at the discretion of the HVTN 054 PSRT.

14.7.3 Monthly review

In addition to the detailed clinical monitoring reports discussed above, protocol-specific summary reports of reactogenicity and AE data are provided to the HVTN Phase I/II Committee in a blinded fashion.

14.7.4 Quarterly review

The HVTN safety monitoring board is composed of the following members, who are not directly affiliated with the protocols under review:

- SMB Chair

FOR COPYING ONLY

- DAIDS Medical Officer representative
- Non-US representative
- US representative
- Statistician
- Clinician
- HVTN Director

The safety monitoring board will review unblinded safety data approximately every quarter. This review is designed to provide confirmation with respect to ad hoc review requests as well as increase overall sensitivity for detecting potential safety problems by looking across multiple protocols that use the same or similar vaccine candidates. The review consists of evaluation of unblinded safety data, including comparisons of adverse experiences in vaccine and placebo recipients in aggregate, as well as review of individual SAE reports. The safety monitoring board will conduct additional special reviews in conjunction with the HVTN 054 PSRT when safety pause rules requiring immediate notification (Table 14-2) are triggered.

14.7.5 Review for dose escalation

In addition to monitoring participant safety throughout the study period, the HVTN 054 Protocol Safety Review Team will review all available safety data collected on all participants in Group 1 through the Day 14 visit and determine whether dose escalation may occur. For this determination, the HVTN 054 PSRT may consult ad hoc with the HVTN safety monitoring board.

The HVTN 054 PSRT will examine the safety and reactogenicity events for all participants in each group to date and will discuss every event that triggers the pause rules and determine the advisability of continuing the dose escalation. At a minimum, if 2 or more participants enrolled in a given group report a Grade IV, vaccine-related reactogenicity or adverse experience, then accrual will be permanently suspended in that group and any other group at a higher dose level. As noted in the pausing rules, any such Grade IV event will require HVTN 054 PSRT consultation with the FDA and HVTN SMB. Accrual may be suspended for any safety concern if, in the judgment of the HVTN 054 PSRT, participant safety is threatened.

FOR COPYING ONLY

References

1. UNAIDS, WHO. AIDS epidemic update. **2002**;UNAIDS/02.46E:1-36.

2. UNAIDS. Report on the global HIV/AIDS epidemic. **2002**;UNAIDS/02.26E.

3. Centers for Disease Control and Prevention. Diagnosis and reporting of HIV and AIDS in states with HIV/AIDS surveillance; United States, 1994-2000. MMWR **2002**;51:595-8.

4. Centers for Disease Control and Prevention. Update: AIDS, United States, 2000. MMWR **2002**;51:592-5.

5. Centers for Disease Control and Prevention. HIV/AIDS surveillance report. **2001**;1-48.

6. Fauci AS. The AIDS epidemic--considerations for the 21st century. N Engl J Med **1999**;341:1046-50.

7. Varmus H, Nathanson N. Science and the control of AIDS. Science **1998**;280:1815.

8. Borrow P, Lewicki H, Hahn BH, Shaw GM, Oldstone MB. Virus-specific CD8+ cytotoxic T-lymphocyte activity associated with control of viremia in primary human immunodeficiency virus type 1 infection. J Virol **1994**;68:6103-10.

9. Koup RA, Safrit JT, Cao Y, et al. Temporal association of cellular immune responses with the initial control of viremia in primary human immunodeficiency virus type 1 syndrome. J Virol **1994**;68:4650-5.

10. Pantaleo G, Demarest JF, Soudeyns H, et al. Major expansion of CD8+ T cells with a predominant V beta usage during the primary immune response to HIV. Nature **1994**;370:463-7.

11. Musey L, Hughes J, Schacker T, Shea T, Corey L, McElrath MJ. Cytotoxic-T-cell responses, viral load, and disease progression in early human immunodeficiency virus type 1 infection. N Engl J Med **1997**;337:1267-74.

12. Altman JD, Moss PA, Goulder PJ, et al. Phenotypic analysis of antigen-specific T lymphocytes. Science **1996**;274:94-6.

13. Jin X, Bauer DE, Tuttleton SE, et al. Dramatic rise in plasma viremia after CD8(+) T cell depletion in simian immunodeficiency virus-infected macaques. J Exp Med **1999**;189:991-8.

14. Schmitz JE, Kuroda MJ, Santra S, et al. Control of viremia in simian immunodeficiency virus infection by CD8+ lymphocytes. Science **1999**;283:857-60.

15. Douek DC, Brenchley JM, Betts MR, et al. HIV preferentially infects HIV-specific CD4+ T cells. Nature **2002**;417:95-8.

16. Yang OO, Kalams SA, Rosenzweig M, et al. Efficient lysis of human immunodeficiency virus type 1-infected cells by cytotoxic T lymphocytes. J Virol **1996**;70:5799-806.

FOR COPYING ONLY

17. Klein MR, van Baalen CA, Holwerda AM, et al. Kinetics of Gag-specific cytotoxic T lymphocyte responses during the clinical course of HIV-1 infection: a longitudinal analysis of rapid progressors and long-term asymptomatics. J Exp Med **1995**;181:1365-72.

18. Moss PA, Rowland-Jones SL, Frodsham PM, et al. Persistent high frequency of human immunodeficiency virus-specific cytotoxic T cells in peripheral blood of infected donors. Proc Natl Acad Sci U S A **1995**;92:5773-7.

19. Nixon DF, Townsend AR, Elvin JG, Rizza CR, Gallwey J, McMichael AJ. HIV-1 gag-specific cytotoxic T lymphocytes defined with recombinant vaccinia virus and synthetic peptides. Nature **1988**;336:484-7.

20. Shiver, J. W. Merck's HIV vaccine program: research approaches and critical issues. AIDS Vaccine 2001. Philadelphia, **2001**.

21. Shiver JW, Fu TM, Chen L, et al. Replication-incompetent adenoviral vaccine vector elicits effective anti-immunodeficiency-virus immunity. Nature **2002**;415:331-5.

22. Sullivan NJ, Sanchez A, Rollin PE, Yang ZY, Nabel GJ. Development of a preventive vaccine for Ebola virus infection in primates. Nature **2000**;408:605-9.

23. Casimiro DR, Tang A, Chen L, et al. Vaccine-induced immunity in baboons by using DNA and replication-incompetent adenovirus type 5 vectors expressing a human immunodeficiency virus type 1 gag gene. J Virol **2003**;77:7663-8.

24. Sullivan NJ, Geisbert TW, Geisbert JB, et al. Accelerated vaccination for Ebola virus haemorrhagic fever in non-human primates. Nature **2003**;424:681-4.

25. Casimiro DR, Chen L, Fu TM, et al. Comparative immunogenicity in rhesus monkeys of DNA plasmid, recombinant vaccinia virus, and replication-defective adenovirus vectors expressing a human immunodeficiency virus type 1 gag gene. J Virol **2003**;77:6305-13.

26. Amara RR, Villinger F, Altman JD, et al. Control of a mucosal challenge and prevention of AIDS by a multiprotein DNA/MVA vaccine. Science **2001**;292:69-74.

27. Approaches to the development of broadly protective HIV vaccines: challenges posed by the genetic, biological and antigenic variability of HIV-1: Report from a meeting of the WHO-UNAIDS Vaccine Advisory Committee Geneva, 21-23 February 2000. AIDS **2001**;15:W1-W25.

28. Osmanov S, Pattou C, Walker N, Schwardlander B, Esparza J. Estimated global distribution and regional spread of HIV-1 genetic subtypes in the year 2000. J Acquir Immune Defic Syndr **2002**;29:184-90.

29. Nabel G, Makgoba W, Esparza J. HIV-1 diversity and vaccine development. Science **2002**;296:2335.

30. Plotkin S, Orenstein WA. Vaccines. Philadelphia: W.B. Saunders Co., **1999**;1230.

31. Schwartz AR, Togo Y, Hornick RB. Clinical evaluation of live, oral types 1, 2, and 5 adenovirus vaccines. Am Rev Respir Dis **1974**;109:233-9.

FOR COPYING ONLY

32. Rosenbaum MJ, Poudstone JW, Edwards EA. Acceptability and antigenicity of adjuvant soluble sub-unit adenovirus vaccines in navy recruits. Mil Med **1976**;141:383-8.

33. Rosenbaum MJ, Edwards EA, Hoeffler DF CAPT. Recent experiences with live adenovirus vaccines in navy recruits. Mil Med **1975**;140:251-7.

34. Gaydos CA, Gaydos JC. Adenovirus vaccines in the U.S. military. Mil Med **1995**;160:300-4.

35. Harvey BG, Leopold PL, Hackett NR, et al. Airway epithelial CFTR mRNA expression in cystic fibrosis patients after repetitive administration of a recombinant adenovirus. J Clin Invest **1999**;104:1245-55.

36. Bellon G, Michel-Calemard L, Thouvenot D, et al. Aerosol administration of a recombinant adenovirus expressing CFTR to cystic fibrosis patients: a phase I clinical trial. Hum Gene Ther **1997**;8:15-25.

37. Hay JG, McElvaney NG, Herena J, Crystal RG. Modification of nasal epithelial potential differences of individuals with cystic fibrosis consequent to local administration of a normal CFTR cDNA adenovirus gene transfer vector. Hum Gene Ther **1995**;6:1487-96.

38. Harvey BG, Worgall S, Ely S, Leopold PL, Crystal RG. Cellular immune responses of healthy individuals to intradermal administration of an E1-E3- adenovirus gene transfer vector. Hum Gene Ther **1999**;10:2823-37.

39. Rosengart TK, Lee LY, Patel SR, et al. Six-month assessment of a phase I trial of angiogenic gene therapy for the treatment of coronary artery disease using direct intramyocardial administration of an adenovirus vector expressing the VEGF121 cDNA. Ann Surg **1999**;230:466-70.

40. Rosengart TK, Lee LY, Patel SR, et al. Angiogenesis gene therapy: phase I assessment of direct intramyocardial administration of an adenovirus vector expressing VEGF121 cDNA to individuals with clinically significant severe coronary artery disease. Circulation **1999**;100:468-74.

41. Sterman DH, Treat J, Litzky LA, et al. Adenovirus-mediated herpes simplex virus thymidine kinase/ganciclovir gene therapy in patients with localized malignancy: results of a phase I clinical trial in malignant mesothelioma. Hum Gene Ther **1998**;9:1083-92.

42. Clayman GL, Frank DK, Bruso PA, Goepfert H. Adenovirus-mediated wild-type p53 gene transfer as a surgical adjuvant in advanced head and neck cancers. Clin Cancer Res **1999**;5:1715-22.

43. Swisher SG, Roth JA, Nemunaitis J, et al. Adenovirus-mediated p53 gene transfer in advanced non-small-cell lung cancer. J Natl Cancer Inst **1999**;91:763-71.

44. Gahery-Segard H, Molinier-Frenkel V, Le Boulaire C, et al. Phase I trial of recombinant adenovirus gene transfer in lung cancer. Longitudinal study of the immune responses to transgene and viral products. J Clin Invest **1997**;100:2218-26.

45. Tursz T, Cesne AL, Baldeyrou P, et al. Phase I study of a recombinant adenovirus-mediated gene transfer in lung cancer patients. J Natl Cancer Inst **1996**;88:1857-63.

FOR COPYING ONLY

46. Crystal RG, Harvey BG, Wisnivesky JP, et al. Analysis of risk factors for local delivery of low- and intermediate-dose adenovirus gene transfer vectors to individuals with a spectrum of comorbid conditions. Hum Gene Ther **2002**;13:65-100.

47. Harvey BG, Maroni J, O'Donoghue KA, et al. Safety of local delivery of low- and intermediate-dose adenovirus gene transfer vectors to individuals with a spectrum of morbid conditions. Hum Gene Ther **2002**;13:15-63.

48. Harvey BG, Hackett NR, El Sawy T, et al. Variability of human systemic humoral immune responses to adenovirus gene transfer vectors administered to different organs. J Virol **1999**;73:6729-42.

49. Rajagopalan S, Trachtenberg J, Mohler E, et al. Phase I study of direct administration of a replication deficient adenovirus vector containing the vascular endothelial growth factor cDNA (CI-1023) to patients with claudication. Am J Cardiol **2002**;90:512-6.

50. Raper SE, Yudkoff M, Chirmule N, et al. A pilot study of in vivo liver-directed gene transfer with an adenoviral vector in partial ornithine transcarbamylase deficiency. Hum Gene Ther **2002**;13:163-75.

51. Assessment of adenoviral vector safety and toxicity: report of the National Institutes of Health Recombinant DNA Advisory Committee. Hum Gene Ther **2002**;13:3-13.

52. Varnavski AN, Zhang Y, Schnell M, et al. Preexisting immunity to adenovirus in rhesus monkeys fails to prevent vector-induced toxicity. J Virol **2002**;76:5711-9.

53. Yang Y, Wilson JM. Clearance of adenovirus-infected hepatocytes by MHC class I-restricted CD4+ CTLs in vivo. J Immunol **1995**;155:2564-70.

54. Yang Y, Nunes FA, Berencsi K, Furth EE, Gonczol E, Wilson JM. Cellular immunity to viral antigens limits E1-deleted adenoviruses for gene therapy. Proc Natl Acad Sci U S A **1994**;91:4407-11.

55. Nunes FA, Furth EE, Wilson JM, Raper SE. Gene transfer into the liver of nonhuman primates with E1-deleted recombinant adenoviral vectors: safety of readministration. Hum Gene Ther **1999**;10:2515-26.

56. Lozier JN, Csako G, Mondoro TH, et al. Toxicity of a first-generation adenoviral vector in rhesus macaques. Hum Gene Ther **2002**;13:113-24.

57. Morral N, O'Neal WK, Rice K, et al. Lethal toxicity, severe endothelial injury, and a threshold effect with high doses of an adenoviral vector in baboons. Hum Gene Ther **2002**;13:143-54.

58. Emini, E. A. A potential HIV-1 vaccine using a replication-defective adenoviral vaccine vector. 9th conference of retroviruses and opportunistic infections. Seattle, **2002**.

59. Emini, E. A. Ongoing Development and Evaluation of a Potential HIV-1 Vaccine Using a Replication-Defective Adenoviral Vector. Keystone Conference on HIV Vaccines. Banff, **2003**.

FOR COPYING ONLY

60. Hofmann C, Loser P, Cichon G, Arnold W, Both GW, Strauss M. Ovine adenovirus vectors overcome preexisting humoral immunity against human adenoviruses in vivo. J Virol **1999**;73:6930-6.

61. Kostense, S, Vogel, S., Lemckert, A., Ophorst, O., Koudstaal, W., Sprangers, M., Goudsmit, J., Havenga, M. Adenovirus serotype 35-based vaccination in the absence or presence of serotype 5 specific immunity. Keystone Conference on HIV Vaccines. Banff, **2003**.

62. Nwanegbo E, Gao W, Gambotto A, Rowe D, Robbins P, Sun H, Whittle H, Vardas E. Prevalence of neutralizing antibody to adenoviral serotype 5 and 35 in the Gambia, South Africa, and United States adult populations. AIDS Vaccine 2003. New York, **2003**.

63. Mast TC, Gupta S, Nikas A, Kierstead L, Kallas EG, Mbewe B, Pitisuttithum P, Schecter M, Vardas E, Wolfe N, Coplan P, Aste-Amezaga M, Shiver J, Emini EA, Straus WL. International epidemiology of human preexisting anti-adenovirus type-5 (Ad5) antibodies in areas representative of potential HIV vaccine trial sites. AIDS Vaccine 2003. New York, **2003**.

64. Sumida S, Truitt D, Koudstaal W, Jackson S, Gorgone D, Lifton M, Kostense S, Pau M, Havenga M, Goudsmit J, Nabel G, Letvin N, Barouch D. Both humoral and cellular immune responses contribute to immunity to adenovirus serotype 5. AIDS Vaccine 2003. New York, **2003**.

65. Barouch D, Pau M, Custers J, Kostense S, Koudstall W, Havenga M, Truitt D, Sumida S, Arthur J, Kishko M, Letvin N, Goudsmit J. Immunogenicity of recombinant adenovirus serotype-35 expressing SIV gag in the presence of anti-Ad5 immunity. AIDS Vaccine 2003. New York, **2003**.

66. Chirmule N, Propert K, Magosin S, Qian Y, Qian R, Wilson J. Immune responses to adenovirus and adeno-associated virus in humans. Gene Ther **1999**;6:1574-83.

67. Pacini DL, Collier AM, Henderson FW. Adenovirus infections and respiratory illnesses in children in group day care. J Infect Dis **1987**;156:920-7.

68. Piedra PA, Poveda GA, Ramsey B, McCoy K, Hiatt PW. Incidence and prevalence of neutralizing antibodies to the common adenoviruses in children with cystic fibrosis: implication for gene therapy with adenovirus vectors. Pediatrics **1998**;101:1013-9.

69. Vogels R, Zuijdgeest D, van Rijnsoever R, et al. Replication-deficient human adenovirus type 35 vectors for gene transfer and vaccination: efficient human cell infection and bypass of preexisting adenovirus immunity. J Virol **2003**;77:8263-71.

70. Seshidhar RP, Ganesh S, Limbach MP, et al. Development of adenovirus serotype 35 as a gene transfer vector. Virology **2003**;311:384-93.

71. Seth P. Adenoviruses: basic biology to gene therapy. Austin, TX: RG Landes Co., **1999**.

72. Brough DE, Lizonova A, Hsu C, Kulesa VA, Kovesdi I. A gene transfer vector-cell line system for complete functional complementation of adenovirus early regions E1 and E4. J Virol **1996**;70:6497-501.

FOR COPYING ONLY

73. Rasmussen H, Rasmussen C, Lempicki M, et al. TNFerade Biologic: preclinical toxicology of a novel adenovector with a radiation-inducible promoter, carrying the human tumor necrosis factor alpha gene. Cancer Gene Ther **2002**;9:951-7.

74. Hudgens MG. Estimating cumulative probabilities from incomplete longitudinal binary responses with application to HIV vaccine trials. Statistics in Medicine **2003**;22:463-79.

FOR COPYING ONLY

Protocol history

The Protocol Team may modify the original version of the protocol. Modifications are made to HVTN protocols via clarification memos, letters of amendment, or full protocol amendments. HVTN protocols are modified and distributed according to the standard HVTN procedures as described in the HVTN MOP (Organization and Policy>Vaccine Selection and Protocol Development).

The table below describes the version history of, and modifications to, Protocol HVTN 054.

Protocol history and modifications

Date	Protocol version	Protocol modification	Comment
06-DEC-04	Version 1	Original protocol	

FOR COPYING ONLY

Protocol team

Information on protocol team member designation and responsibilities and on the protocol development process can be found in the HVTN MOP. Contact information for protocol team members, HVTUs, and labs can be found in the *HVTN 054 Study Specific Procedures*.

PROTOCOL TEAM REDACTED

FOR COPYING ONLY

This page is intentionally blank

FOR COPYING ONLY

APPENDICES

FOR COPYING ONLY

Appendix B: Schedule of laboratory procedures

						Tube volume (mL)						
					Visit							
				Visit:	1	2	3	4	5	6	7	
				Day:	Screening visit	D0	D14	D28	D84	D168	D364	
				Month:		M0	M0.5	M1	M3	M6	M12	
Procedure	Ship to	Assay location	Tube			**VAC1**					**Final**	**Total**
**Humoral assays**												
HIV Screening ELISA	Local lab	Local lab	SST		5	—	—	—	—	—	—	**5**
HIV diagnostic ELISA	CL-Richmond	CL-Richmond	SST		—	—	—	—	5	5	5	**15**
HIV Neut. antibody (Ab)	CSR	CL-Duke	SST		—	5	—	5	—	—	5	**15**
HIV Binding ELISA	CSR	CL-Duke	SST		—	5	—	5	—	—	5	**15**
Ad5 Neut. Ab studies	CSR	VRC	SST		5	—	—	5	—	5	5	**20**
**Viral assays**												
HIV RNA PCR	Richmond	CL-Richmond	PPT		—	5	—	—	5	5	5	**20**
HIV DNA PCR	Richmond	CL-Richmond	ACD		—	7	—	—	7	7	7	**28**
**Cellular assays**												
ELISpot/ICS	CSR	CL-FHCRC	Na Hep		—	80	—	80	—	—	80	**240**
ICS	CSR	VRC	Na Hep		—	40	40	40	—	40	40	**200**
HLA typing	Duke	CL-Duke	ACD		—	20	—	—	—	—	—	**20**
**Specimen storage**												
PBMC	CSR	CSR	Na Hep		—	—	—	60	150	110	150	**470**
Serum	CSR	CSR	SST		—	10	10	20	20	20	20	**100**
**Safety labs**												
CBC/differential/platelets	Local lab	Local lab	EDTA		5	—	5	5	5	5	5	**30**
Chemistry panel	Local lab	Local lab	SST		10	—	5	5	5	5	5	**35**
**Total**					**25**	**172**	**60**	**225**	**197**	**202**	**332**	**1213**
56-Day total					**25**	**197**	**257**	**482**	**422**	**202**	**332**	
**Urine collection**												
Urinalysis					X	—	X	—	—	X	—	
Pregnancy test					X	X	—	X	—	—	—	

CSR = Central Specimen Repository; CL = Central Laboratory

The HVTN Laboratory Program includes Central Laboratories at Richmond, Duke, FHCRC, and SAIL-NICD. Richmond = Viral and Rickettsial Disease Laboratory, California Department of Health Services (Richmond, California, USA); Duke = Duke University Medical Center (Durham, North Carolina, USA); FHCRC/UW = Fred Hutchinson Cancer Research Center/University of Washington (Seattle, Washington, USA); SAIL-NICD = South African Immunology Laboratory–National Institute for Communicable Diseases (Johannesburg, South Africa)

Screening may occur over the course of several contacts/visits up to and including Day 0 prior to vaccination. Additional tests performed at the screening visit include syphilis, HepB and HepC tests using serum samples.

Diagnostic HIV Western blot will be performed at CL-Richmond as indicated from samples sent for diagnostic ELISA per the HVTN algorithm for diagnosis of HIV infections

Humoral and cellular assays at Day 84, Day 168, and Day 364 may be performed if a positive response is observed in the same assay at 4 weeks post-vaccination.

HLA-typing will be performed only when positive responses to cellular assays are observed. These data will be used to guide immunological assays of future interest.

Serum for the screening of pre-existing adenovirus titers will be sent directly to the VRC.

A portion of serum for storage will be reserved for humoral studies at VRC.

FOR COPYING ONLY

Appendix C: Schedule of procedures at HVTU

	Visit	01	02	03	04	05	06	07	
	Day	Prior to VAC1	D0	D14	D28	D84	D168	D364	
	Month		M0	M 0.5	M1	M3	M6	M12	
		Screening[a]	**VAC**						Post
**Study Procedures**	Signed screening consent (if used)	X[b]							
	Assessment of understanding	X							
	Signed protocol consent	X[b]							
	Medical history	X							
	Complete physical exam	X						X	
	Abbreviated physical exam		X	X	X	X	X		
	Risk reduction/pregnancy prevention counseling	X	X	X	X	X	X	X	
	Behavioral risk assessment	X							
	Confirm eligibility, obtain demographics, randomize participant	X							
	Social impact assessment					X		X	
	Outside testing and belief questionnaire						X	X	
	Concomitant medications	X	X	X	X	X	X	X	
	Intercurrent illness / adverse experience		X	X	X	X	X	X	
	Specimen collection (see Appendix B)	X	X	X	X	X	X	X	
	HIV infection assessment/results[c]	X				X	X	X	
	Adenovirus neutralizing antibody	X[d]			X		X	X	
	Adenovirus culture[e]			X	X				
**Local Lab Assessment**	Pregnancy (urine or serum HCG)	X[f]	X[f]		X				
	Urine dipstick	X		X			X		
	CBC, differential, platelet	X		X	X	X	X	X	
	RPR	X							
	Hepatitis B, Hepatitis C	X							
	Chemistry panel (see Table 12-1 and 12-2)	X		X	X	X	X	X	
**Vaccination Procedures**	Vaccination		X						
	Reactogenicity assessments[g]		X						
**Post-study**	Unblind participant[h]								X

[a] Screening may occur over the course of several contacts/visits up to and including Day 0 prior to vaccination. All Inclusion/Exclusion criteria must be assessed within 56 days prior to study enrollment, unless otherwise specified in Table 7-1 and Table 7-2.
[b] Appropriate consent forms must be signed prior to performing procedures.
[c] Includes pre- and post-test counseling and follow-up contact to report results to participant.
[d] Screening for adenovirus neutralizing antibody will be performed at the VRC.
[e] Participants who develop symptoms of upper respiratory infection (URI) or conjunctivitis within the four-week period following administration of the study injection will be asked to return to the study site for a throat swab (URI) or conjunctival swab (conjunctivitis) for adenovirus culture. If culture reveals the presence of adenovirus, PCR analysis will be performed.
[f] Pregnancy test must be performed no more than 3 days prior to vaccination. Pregnancy test to determine eligibility can be performed at screening or on Day 0 prior to vaccination. Serum pregnancy tests may be used to confirm the results of or substitute for a urine pregnancy test.
[g] Reactogenicity assessments performed daily for up to 3 days post-vaccination (see Section 14.1).
[h] Participant unblinding occurs only after all enrolled participants have completed the final study visit.

FOR COPYING ONLY

Clarification Memo #1

February 10, 2005

Protocol HVTN 054, Version 1:

A Phase I dose-escalation clinical trial to evaluate the safety and immunogenicity of a multiclade, multivalent recombinant adenoviral vector HIV vaccine, VRC-HIVADV014-00-VP, in healthy, HIV-1 uninfected adult participants who have low titers of pre-existing Ad5 neutralizing antibodies

[BB IND #11661—HELD BY DAIDS]

HVTU Filing Instructions

Please distribute this clarification memo to all appropriate staff members, and file with your protocol documents. Consult your local IRB/Ethics Committee regarding submission requirements for clarification memos.

List of Changes

Item 1 Weekly safety reports are annotated as necessary 2
Item 2 Specimens for Ad5 neutralizing antibody studies at screening shipped directly to VRC......... 3
Item 3 Specimens for HLA typing sent to the central specimen repository....................................... 3
Item 4 Pregnancy tests performed on the day of vaccination prior to vaccination 3

The changes described herein will be incorporated in the next version of Protocol HVTN 054 if it undergoes full protocol amendment at a later time.

Item 1 Weekly safety reports are annotated as necessary

Weekly safety reports are annotated as necessary, as suggested by the sentence in the first paragraph of Section 14.7.2, *Weekly review*, “Values identified during the review that are considered questionable, inconsistent, or unexplained are referred to the HVTU clinic coordinator for verification.” The language in the last paragraph, however, suggests all safety reports reviewed weekly during the vaccination phase are annotated. In Section 14.7.2, *Weekly review*, the last sentence has been deleted, and the second to last sentence in the section has been revised for clarity and moved to the second sentence in the first paragraph.

Previous:

“Blinded weekly safety reviews are routinely performed by the SDMC clinical affairs staff and by the HVTN 054 Protocol Safety Review Team. The SDMC clinical affairs staff reviews reports of all clinical values that fall outside of the standard HVTN safety parameters (see HVTN MOP [Study Operations>Standard Reports>Clinical Safety Review>Weekly Safety Review Reports]). Values identified during the review that are considered questionable, inconsistent, or unexplained are referred to the HVTU clinic coordinator for verification.

The HVTN 054 Protocol Safety Review Team is composed of the following required members:

- Protocol chair, co-chair
- HVTN clinical trials physician
- SDMC clinical affairs staff member
- DAIDS medical officer

The protocol team clinic coordinator, protocol specialist, and vaccine developer representative may also be included at the request of the HVTN 054 PSRT.

Weekly during the vaccination phase of the trial, the HVTN 054 PSRT reviews an annotated version of the appropriate clinical safety reports containing queries to the HVTU. After the vaccinations and the final 2-week safety visits are completed, less frequent safety reviews may be scheduled at the discretion of the HVTN 054 PSRT.”

Revised (underline and strikethrough):

“Blinded weekly safety reviews are routinely performed by the SDMC clinical affairs staff and by the HVTN 054 Protocol Safety Review Team. Once the safety visits two weeks post-vaccination for all participants are complete, less frequent safety reviews may be scheduled at the discretion of the HVTN 054 Protocol Safety Review Team. The SDMC clinical affairs staff reviews reports of all clinical values that fall outside of the standard HVTN safety parameters (see HVTN MOP [Study Operations>Standard Reports>Clinical Safety Review>Weekly Safety Review Reports]). Values identified during the review that are considered questionable, inconsistent, or unexplained are referred to the HVTU clinic coordinator for verification.

The HVTN 054 Protocol Safety Review Team is composed of the following required members:

- Protocol chair, co-chair
- HVTN clinical trials physician
- SDMC clinical affairs staff member
- DAIDS medical officer

The protocol team clinic coordinator, protocol specialist, and vaccine developer representative may also be included at the request of the HVTN 054 PSRT.

~~Weekly during the vaccination phase of the trial, the HVTN 054 PSRT reviews an annotated version of the appropriate clinical safety reports containing queries to the HVTU. After the vaccinations and the final 2-week safety visits are completed, less frequent safety reviews may be scheduled at the discretion of the HVTN 054 PSRT.~~"

Item 2 Specimens for Ad5 neutralizing antibody studies at screening shipped directly to VRC

Specimens for Ad5 neutralizing antibody studies are sent directly to the Vaccine Research Center (VRC) at screening and to the central specimen repository (CSR) for later visits. For additional clarity, the following revisions have been made in the table in Appendix B, *Schedule of laboratory procedures* and are included in the attached, revised Appendix B.

A Ship to location for "Ad5 Neut. Ab studies"

Previous: "CSR"

Revised: "VRC/CSR"

B Seventh footnote

Added (underlined): "Serum for the screening of pre-existing adenovirus titers will be sent directly to the VRC. <u>Serum for assessment of adenovirus titers on subsequent visits will be sent to the CSR.</u>**"**

Item 3 Specimens for HLA typing sent to the central specimen repository

As indicated in Section 8.6 and in a footnote in Appendix B, HLA typing will be performed when post vaccination positive responses to cellular assays are seen. Since the testing of the specimens collected on Day 0 for HLA typing will be done at a future time, the specimens will be sent to the central specimen repository (CSR) to process and store the samples until the HLA typing is needed. In Appendix B, *Schedule of laboratory procedures*, the ship to location for "HLA typing" has been changed from "Duke" to "CSR" as indicated in the attached, revised Appendix B.

Item 4 Pregnancy tests performed on the day of vaccination prior to vaccination

The current HVTN procedures require pregnancy tests to be performed on the day of vaccination prior to vaccination. The inclusion criterion is consistent with this requirement, but a footnote in Appendix C reflects the older policy of performing pregnancy tests within 3 days prior to vaccination. The footnote in Appendix C, *Schedule of procedures at HVTU*, has been modified for consistency with current requirements as indicated below and in the attached, revised Appendix C.

Previous: "Pregnancy test must be performed no more than 3 days prior to vaccination."

Revised: "Pregnancy test must be performed on the day of vaccination prior to vaccination."

Appendix B: Schedule of laboratory procedures

					Tube volume (mL)							
					Visit							
				Visit:	**1**	**2**	**3**	**4**	**5**	**6**	**7**	
				Day:	Screening visit	D0	D14	D28	D84	D168	D364	
				Month:		M 0	M 0.5	M 1	M 3	M 6	M 12	
Procedure	Ship to	Assay location	Tube			**VAC1**					**Final**	**Total**
**Humoral assays**												
HIV Screening ELISA	Local lab	Local lab	SST		5	—	—	—	—	—	—	**5**
HIV diagnostic ELISA	CL-Richmond	CL-Richmond	SST		—	—	—	—	5	5	5	**15**
HIV Neut. antibody (Ab)	CSR	CL-Duke	SST		—	5	—	5	—	—	5	**15**
HIV Binding ELISA	CSR	CL-Duke	SST		—	5	—	5	—	—	5	**15**
Ad5 Neut. Ab studies	VRC/CSR	VRC	SST		5	—	—	5	—	5	5	**20**
**Viral assays**												
HIV RNA PCR	Richmond	CL-Richmond	PPT		—	5	—	—	5	5	5	**20**
HIV DNA PCR	Richmond	CL-Richmond	ACD		—	7	—	—	7	7	7	**28**
**Cellular assays**												
ELISpot/ICS	CSR	CL-FHCRC	Na Hep		—	80	—	80	—	—	80	**240**
ICS	CSR	VRC	Na Hep		—	40	40	40	—	40	40	**200**
HLA typing	CSR	CL-Duke	ACD		—	20	—	—	—	—	—	**20**
**Specimen storage**												
PBMC	CSR	CSR	Na Hep		—	—	—	60	150	110	150	**470**
Serum	CSR	CSR	SST		—	10	10	20	20	20	20	**100**
**Safety labs**												
CBC/differential/platelets	Local lab	Local lab	EDTA		5	—	5	5	5	5	5	**30**
Chemistry panel	Local lab	Local lab	SST		10	—	5	5	5	5	5	**35**
**Total**					**25**	**172**	**60**	**225**	**197**	**202**	**332**	**1213**
56-Day total					**25**	**197**	**257**	**482**	**422**	**202**	**332**	
**Urine collection**												
Urinalysis					X	—	X	—	—	X	—	
Pregnancy test					X	X	—	X	—	—	—	

CSR = Central Specimen Repository; CL = Central Laboratory

The HVTN Laboratory Program includes Central Laboratories at Richmond, Duke, FHCRC, and SAIL-NICD. Richmond = Viral and Rickettsial Disease Laboratory, California Department of Health Services (Richmond, California, USA); Duke = Duke University Medical Center (Durham, North Carolina, USA); FHCRC/UW = Fred Hutchinson Cancer Research Center/University of Washington (Seattle, Washington, USA); SAIL-NICD = South African Immunology Laboratory–National Institute for Communicable Diseases (Johannesburg, South Africa)

Screening may occur over the course of several contacts/visits up to and including Day 0 prior to vaccination. Additional tests performed at the screening visit include syphilis, HepB and HepC tests using serum samples.

Diagnostic HIV Western blot will be performed at CL-Richmond as indicated from samples sent for diagnostic ELISA per the HVTN algorithm for diagnosis of HIV infections

Humoral and cellular assays at Day 84, Day 168, and Day 364 may be performed if a positive response is observed in the same assay at 4 weeks post-vaccination.

HLA-typing will be performed only when positive responses to cellular assays are observed. These data will be used to guide immunological assays of future interest.

Serum for the screening of pre-existing adenovirus titers will be sent directly to the VRC. Serum for assessment of adenovirus titers on subsequent visits will be sent to the CSR.

A portion of serum for storage will be reserved for humoral studies at VRC.

Appendix C: Schedule of procedures at HVTU

	Visit	01	02	03	04 05	06		07	
	Day	Prior to VAC1	D0	D14	D28	D84	D168	D364	
	Month		M0	M 0.5	M1	M3	M6	M12	
		Screening[a]	**VAC**						Post
**Study Procedures**	Signed screening consent (if used)	X[b]							
	Assessment of understanding	X							
	Signed protocol consent	X[b]							
	Medical history	X							
	Complete physical exam	X						X	
	Abbreviated physical exam		X	X	X	X	X		
	Risk reduction/pregnancy prevention counseling	X	X	X	X	X	X	X	
	Behavioral risk assessment	X							
	Confirm eligibility, obtain demographics, randomize participant	X							
	Social impact assessment					X		X	
	Outside testing and belief questionnaire						X	X	
	Concomitant medications	X	X	X	X	X	X	X	
	Intercurrent illness / adverse experience		X	X	X	X	X	X	
	Specimen collection (see Appendix B)	X	X	X	X	X	X	X	
	HIV infection assessment/results[c]	X				X	X	X	
	Adenovirus neutralizing antibody	X[d]			X		X	X	
	Adenovirus culture[e]			X	X				
**Local Lab Assessment**	Pregnancy (urine or serum HCG)	X[f]	X[f]		X				
	Urine dipstick	X		X			X		
	CBC, differential, platelet	X		X	X	X	X	X	
	RPR	X							
	Hepatitis B, Hepatitis C	X							
	Chemistry panel (see Table 12-1 and 12-2)	X		X	X	X	X	X	
**Vaccination Procedures**	Vaccination		X						
	Reactogenicity assessments[g]		X						
**Post-study**	Unblind participant[h]								X

[a] Screening may occur over the course of several contacts/visits up to and including Day 0 prior to vaccination. All Inclusion/Exclusion criteria must be assessed within 56 days prior to study enrollment, unless otherwise specified in Table 7-1 and Table 7-2.
[b] Appropriate consent forms must be signed prior to performing procedures.
[c] Includes pre- and post-test counseling and follow-up contact to report results to participant.
[d] Screening for adenovirus neutralizing antibody will be performed at the VRC.
[e] Participants who develop symptoms of upper respiratory infection (URI) or conjunctivitis within the four-week period following administration of the study injection will be asked to return to the study site for a throat swab (URI) or conjunctival swab (conjunctivitis) for adenovirus culture. If culture reveals the presence of adenovirus, PCR analysis will be performed.
[f] Pregnancy test must be performed on the day of vaccination prior to vaccination. Pregnancy test to determine eligibility can be performed at screening or on Day 0 prior to vaccination. Serum pregnancy tests may be used to confirm the results of or substitute for a urine pregnancy test.
[g] Reactogenicity assessments performed daily for up to 3 days post-vaccination (see Section 14.1).
[h] Participant unblinding occurs only after all enrolled participants have completed the final study visit.

Protocol Modification History

Protocol modifications are made to HVTN protocols via clarification memos, letters of amendment, or full protocol amendments. HVTN protocols are modified and distributed according to the standard HVTN procedures as described in the HVTN MOP (Organization and Policy>Vaccine Selection and Protocol Development).

The table below describes the version history of, and modifications to, Protocol HVTN 054.

Date	Protocol version	Protocol modification	Summary of modifications
10-Feb-05	Version 1	Clarification Memo 1	Item 1 Weekly safety reports are annotated as necessary<br>Item 2 Specimens for Ad5 neutralizing antibody studies at screening shipped directly to VRC<br>Item 3 Specimens for HLA typing sent to the central specimen repository<br>Item 4 Pregnancy tests performed on the day of vaccination prior to vaccination
06-Dec-04	Version 1	N/A	Original protocol

FINAL

Clarification Memo #2

August 26, 2005

Protocol HVTN 054, Version 1:

A Phase I dose-escalation clinical trial to evaluate the safety and immunogenicity of a multiclade, multivalent recombinant adenoviral vector HIV vaccine, VRC-HIVADV014-00-VP, in healthy, HIV-1 uninfected adult participants who have low titers of pre-existing Ad5 neutralizing antibodies

[BB IND #11661—HELD BY DAIDS]

HVTU Filing Instructions

Please distribute this clarification memo to all appropriate staff members, and file with your protocol documents. Consult your local IRB/Ethics Committee regarding submission requirements for clarification memos.

List of Changes

Item 1 Permission for local labs to use appropriate alternative tube types for locally performed tests 1

The changes described herein will be incorporated in the next version of Protocol HVTN 054 if it undergoes full protocol amendment at a later time.

Item 1 Permission for local labs to use appropriate alternative tube types for locally performed tests

A template footnote in Appendix B: Schedule of Laboratory Procedures was inadvertently omitted from the protocol HVTN 054 document. This footnote specifies that local labs are permitted to use appropriate alternative tube types for locally performed tests.

Added: Local labs may assign appropriate alternative tube types for locally performed tests.

Previous:

CSR = Central Specimen Repository; CL = Central Laboratory

The HVTN Laboratory Program includes Central Laboratories at Richmond, Duke, FHCRC, and SAIL-NICD. Richmond = Viral and Rickettsial Disease Laboratory, California Department of Health Services (Richmond, California, USA); Duke = Duke University Medical Center (Durham, North Carolina, USA); FHCRC/UW = Fred Hutchinson Cancer Research Center/University of Washington (Seattle, Washington, USA); SAIL-NICD = South African Immunology Laboratory–National Institute for Communicable Diseases (Johannesburg, South Africa)

Screening may occur over the course of several contacts/visits up to and including Day 0 prior to vaccination. Additional tests performed at the screening visit include syphilis, HepB and HepC tests using serum samples.

Diagnostic HIV Western blot will be performed at CL-Richmond as indicated from samples sent for diagnostic ELISA per the HVTN algorithm for diagnosis of HIV infections

Humoral and cellular assays at Day 84, Day 168, and Day 364 may be performed if a positive response is observed in the same assay at 4 weeks post-vaccination.

HLA-typing will be performed only when positive responses to cellular assays are observed. These data will be used to guide immunological assays of future interest.

Serum for the screening of pre-existing adenovirus titers will be sent directly to the VRC.

A portion of serum for storage will be reserved for humoral studies at VRC.

Revised:

CSR = Central Specimen Repository; CL = Central Laboratory

The HVTN Laboratory Program includes Central Laboratories at Richmond, Duke, FHCRC, and SAIL-NICD. Richmond = Viral and Rickettsial Disease Laboratory, California Department of Health Services (Richmond, California, USA); Duke = Duke University Medical Center (Durham, North Carolina, USA); FHCRC/UW = Fred Hutchinson Cancer Research Center/University of Washington (Seattle, Washington, USA); SAIL-NICD = South African Immunology Laboratory–National Institute for Communicable Diseases (Johannesburg, South Africa)

Screening may occur over the course of several contacts/visits up to and including Day 0 prior to vaccination. Additional tests performed at the screening visit include syphilis, HepB and HepC tests using serum samples.

Diagnostic HIV Western blot will be performed at CL-Richmond as indicated from samples sent for diagnostic ELISA per the HVTN algorithm for diagnosis of HIV infections

Local labs may assign appropriate alternative tube types for locally performed tests.

Humoral and cellular assays at Day 84, Day 168, and Day 364 may be performed if a positive response is observed in the same assay at 4 weeks post-vaccination.

HLA-typing will be performed only when positive responses to cellular assays are observed. These data will be used to guide immunological assays of future interest.

Serum for the screening of pre-existing adenovirus titers will be sent directly to the VRC.

A portion of serum for storage will be reserved for humoral studies at VRC.

Protocol Modification History

Protocol modifications are made to HVTN protocols via clarification memos, letters of amendment, or full protocol amendments. HVTN protocols are modified and distributed according to the standard HVTN procedures as described in the HVTN MOP (Organization and Policy>Vaccine Selection and Protocol Development).

The table below describes the version history of, and modifications to, Protocol HVTN 054.

Date	Protocol version	Protocol modification	Summary of modifications
10-Feb-05	Version 1	Clarification Memo 1	Item 1 Weekly safety reports are annotated as necessary<br>Item 2 Specimens for Ad5 neutralizing antibody studies at screening shipped directly to VRC<br>Item 3 Specimens for HLA typing sent to the central specimen repository<br>Item 4 Pregnancy tests performed on the day of vaccination prior to vaccination
06-Dec-04	Version 1	N/A	Original protocol

HIV VACCINE
TRIALS NETWORK

FINAL

Clarification Memo #3

October 12, 2005

Protocol HVTN 054, Version 1:

A Phase I dose-escalation clinical trial to evaluate the safety and immunogenicity of a multiclade, multivalent recombinant adenoviral vector HIV vaccine, VRC-HIVADV014-00-VP, in healthy, HIV-1 uninfected adult participants who have low titers of pre-existing Ad5 neutralizing antibodies

[BB IND #11661—HELD BY DAIDS]

HVTU Filing Instructions

Please distribute this clarification memo to all appropriate staff members, and file with your protocol documents. Consult your local IRB/Ethics Committee regarding submission requirements for clarification memos.

List of Changes

Item 1 Discontinued: Specimen collection for HIV DNA PCR testing 1

The changes described herein will be incorporated in the next version of Protocol HVTN 054 if it undergoes full protocol amendment at a later time.

Item 1 Discontinued: Specimen collection for HIV DNA PCR testing

Protocol HVTN 054 (Table 12-2, Appendix B) instructs sites to collect specimens for HIV DNA PCR and HIV RNA PCR testing at Visits 02, 05, 06, and 07. A PCR test (either DNA or RNA) is a requirement of the HVTN HIV infection algorithm along with EIA and Western blot. The HVTN has routinely collected specimens for both HIV DNA and HIV RNA PCR tests. However, the HIV infection algorithm requires only that one or the other of the tests be performed. Access to the DNA PCR test at some non-US HVTUs has been limited.

Furthermore, a recent change by the test kit manufacturer limits the availability of the reagent normally used by the US processing laboratories for the DNA PCR test. The HVTN and DAIDS, in consultation with the FDA, have agreed that routine collection of specimens for both kinds of PCR testing is unnecessary. Therefore, routine collection of specimens for HIV DNA PCR testing

is to be discontinued. The HVTN Laboratory Program will use specimens collected for HIV RNA PCR to satisfy the HIV PCR test indicated in the HVTN HIV infection algorithm.

Per this modification, Table 12-2 and Appendix B have been modified to reflect discontinuation of specimen collection for HIV DNA PCR testing. The revised Appendix B is attached. The revised Table 12-2 is shown below.

This change has no impact on participant safety. It reduces slightly the volume of blood drawn from each participant.

Table 12-2, HIV infection assessments:

**Previous:**	**Revised:**
HIV ELISA	HIV ELISA
HIV Western blot (if applicable)	HIV Western blot (if applicable)
HIV RNA PCR	HIV RNA PCR
HIV DNA PCR	

Appendix B: Schedule of laboratory procedures

Procedure	Ship to	Assay location	Tube	Visit 1 Screening visit	Visit 2 D0 M0 VAC1	Visit 3 D14 M0.5	Visit 4 D28 M1	Visit 5 D84 M3	Visit 6 D168 M6	Visit 7 D364 M12 Final	Total
**Humoral assays**											
HIV Screening ELISA	Local lab	Local lab	SST	5	—	—	—	—	—	—	**5**
HIV diagnostic ELISA	CL-Richmond	CL-Richmond	SST	—	—	—	—	5	5	5	**15**
HIV Neut. antibody (Ab)	CSR	CL-Duke	SST	—	5	—	5	—	—	5	**15**
HIV Binding ELISA	CSR	CL-Duke	SST	—	5	—	5	—	—	5	**15**
Ad5 Neut. Ab studies	VRC/CSR	VRC	SST	5	—	—	5	—	5	5	**20**
**Viral assays**											
HIV RNA PCR	Richmond	CL-Richmond	PPT	—	5	—	—	5	5	5	**20**
**Cellular assays**											
ELISpot/ICS	CSR	CL-FHCRC	Na Hep	—	80	—	80	—	—	80	**240**
ICS	CSR	VRC	Na Hep	—	40	40	40	—	40	40	**200**
HLA typing	CSR	CL-Duke	ACD	—	20	—	—	—	—	—	**20**
**Specimen storage**											
PBMC	CSR	CSR	Na Hep	—	—	—	60	150	110	150	**470**
Serum	CSR	CSR	SST	—	10	10	20	20	20	20	**100**
**Safety labs**											
CBC/differential/platelets	Local lab	Local lab	EDTA	5	—	5	5	5	5	5	**30**
Chemistry panel	Local lab	Local lab	SST	10	—	5	5	5	5	5	**35**
**Total**				**25**	**165**	**60**	**225**	**190**	**195**	**325**	**1185**
56-Day total				**25**	**190**	**250**	**475**	**415**	**195**	**325**	
**Urine collection**											
Urinalysis				X	—	X	—	—	X	—	
Pregnancy test				X	X	—	X	—	—	—	

Tube volume (mL) by Visit.

CSR = Central Specimen Repository; CL = Central Laboratory

The HVTN Laboratory Program includes Central Laboratories at Richmond, Duke, FHCRC, and SAIL-NICD. Richmond = Viral and Rickettsial Disease Laboratory, California Department of Health Services (Richmond, California, USA); Duke = Duke University Medical Center (Durham, North Carolina, USA); FHCRC/UW = Fred Hutchinson Cancer Research Center/University of Washington (Seattle, Washington, USA); SAIL-NICD = South African Immunology Laboratory–National Institute for Communicable Diseases (Johannesburg, South Africa)

Screening may occur over the course of several contacts/visits up to and including Day 0 prior to vaccination. Additional tests performed at the screening visit include syphilis, HepB and HepC tests using serum samples.

Diagnostic HIV Western blot will be performed at CL-Richmond as indicated from samples sent for diagnostic ELISA per the HVTN algorithm for diagnosis of HIV infections

Local labs may assign appropriate alternative tube types for locally performed tests.

Humoral and cellular assays at Day 84, Day 168, and Day 364 may be performed if a positive response is observed in the same assay at 4 weeks post-vaccination.

HLA-typing will be performed only when positive responses to cellular assays are observed. These data will be used to guide immunological assays of future interest.

Serum for the screening of pre-existing adenovirus titers will be sent directly to the VRC.

A portion of serum for storage will be reserved for humoral studies at VRC.

Protocol Modification History

Protocol modifications are made to HVTN protocols via clarification memos, letters of amendment, or full protocol amendments. HVTN protocols are modified and distributed according to the standard HVTN procedures as described in the HVTN MOP (Organization and Policy>Vaccine Selection and Protocol Development).

The table below describes the version history of, and modifications to, Protocol HVTN 054.

Date	Protocol version	Protocol modification	Summary of modifications
12-Oct-05	Version 1	Clarification Memo 3	Item 1 Discontinued: Specimen collection for HIV DNA PCR testing
26-Aug-05	Version 1	Clarification Memo 2	Item 1 Permission for local labs to use appropriate alternative tube types for locally performed tests
10-Feb-05	Version 1	Clarification Memo 1	Item 1 Weekly safety reports are annotated as necessary<br>Item 2 Specimens for Ad5 neutralizing antibody studies at screening shipped directly to VRC<br>Item 3 Specimens for HLA typing sent to the central specimen repository<br>Item 4 Pregnancy tests performed on the day of vaccination prior to vaccination
06-Dec-04	Version 1	N/A	Original protocol

Clarification Memo #4

February 13, 2006

Protocol HVTN 054, Version 1:

A Phase I dose-escalation clinical trial to evaluate the safety and immunogenicity of a multiclade, multivalent recombinant adenoviral vector HIV vaccine, VRC-HIVADV014-00-VP, in healthy, HIV-1 uninfected adult participants who have low titers of pre-existing Ad5 neutralizing antibodies

[BB IND #11661—HELD BY DAIDS]

HVTU Filing Instructions

Please distribute this clarification memo to all appropriate staff members, and file with your protocol documents. Consult your local IRB/Ethics Committee regarding submission requirements for clarification memos.

List of Changes

Item 1 Replaced: Viral and Rickettsial Disease Laboratory (Richmond, California) by University of Washington Virology Specialty Laboratory (Seattle, Washington) 2

Item 2 Changed: Tube type for HIV RNA PCR specimens from PPT to EDTA in Appendix B 3

The changes described herein will be incorporated in the next version of Protocol HVTN 054 if it undergoes full protocol amendment at a later time.

Item 1 Replaced: Viral and Rickettsial Disease Laboratory (Richmond, California) by University of Washington Virology Specialty Laboratory (Seattle, Washington)

Protocol HVTN 054 Appendix B, *Schedule of laboratory procedures,* indicates that the Viral and Rickettsial Disease Laboratory (Richmond, California) is the central testing lab for HIV infection. The HVTN Laboratory Program is changing the location for central HIV testing from the Viral and Rickettsial Disease Laboratory in Richmond, California to the University of Washington Virology Specialty Laboratory (UW-VSL) in Seattle, Washington. HVTUs will be notified of the exact date of the change for specimen shipping purposes.

Per this modification, both the table and footnotes in Appendix B have been revised to indicate the UW-VSL will be the ship to and assay location of HIV testing (see attached).

Please note that sites may continue to perform HIV testing locally, if approved by the HVTN Laboratory Program.

This modification has no effect on participant safety.

A Laboratory designation changed in Overview

Previous:

"Central laboratories

Duke University Medical Center (Durham, North Carolina, USA)

FHCRC/University of Washington

Viral and Rickettsial Disease Laboratory (Richmond, California, USA)

South Africa Immunology Laboratory and National Institute for Communicable Disease (Johannesburg, South Africa)"

Revised:

"Endpoint laboratories

Duke University Medical Center (Durham, North Carolina, USA)

FHCRC/University of Washington (Seattle, Washington, USA)

University of Washington Virology Specialty Laboratory (Seattle, Washington, USA)

South Africa Immunology Laboratory and National Institute for Communicable Disease (Johannesburg, South Africa)"

B Laboratory designation changed in footnote 2 to Appendix B

Previous:

"The HVTN Laboratory Program includes Central Laboratories at Richmond, Duke, FHCRC, and SAIL-NICD. Richmond = Viral and Rickettsial Disease Laboratory, California Department of Health Services (Richmond, California, USA); Duke = Duke University Medical Center (Durham, North Carolina, USA); FHCRC/UW = Fred Hutchinson Cancer Research Center/University of Washington (Seattle, Washington, USA); SAIL-NICD = South African Immunology Laboratory–National Institute for Communicable Diseases (Johannesburg, South Africa)"

Revised:

"The HVTN Laboratory Program includes endpoint laboratories at UW-VSL, Duke, FHCRC, and SAIL-NICD. UW-VSL = University of Washington Virology Specialty Laboratory (Seattle, Washington, USA); Duke = Duke University Medical Center (Durham, North Carolina, USA); FHCRC/UW = Fred Hutchinson Cancer Research Center/University of Washington (Seattle, Washington, USA); SAIL-NICD = South African Immunology Laboratory–National Institute for Communicable Diseases (Johannesburg, South Africa)"

C Laboratory designation changed in footnote 4 to Appendix B

Previous:

"Diagnostic HIV Western blot will be performed at CL-Richmond as indicated from samples sent for diagnostic ELISA per the HVTN algorithm for diagnosis of HIV infections"

Revised:

"Diagnostic HIV Western blot will be performed at UW-VSL as indicated from samples sent for diagnostic ELISA per the HVTN algorithm for diagnosis of HIV infections"

Item 2 Changed: Tube type for HIV RNA PCR specimens from PPT to EDTA in Appendix B

Protocol HVTN 054 Appendix B, *Laboratory procedures*, indicates that specimens for HIV RNA PCR are to be collected in PPT tubes. The University of Washington Virology Specialty Laboratory has not validated plasma from PPT tubes in their HIV RNA PCR assay. EDTA tubes have been validated by the HIV RNA kit manufacturer. Therefore we are transitioning from PPT tubes to EDTA tubes for HIV RNA PCR specimen collection. This change is incorporated in the attached Appendix B. HVTUs will be notified of the exact date of this change.

This modification has no effect on participant safety.

Appendix B: Schedule of laboratory procedures

						Tube volume (mL)						
						Visit						
				Visit:	**1**	**2**	**3**	**4**	**5**	**6**	**7**	
				Day:	Screening visit	D0	D14	D28	D84	D168	D364	
				Month:		M0	M0.5	M1	M3	M6	M12	
Procedure	Ship to	Assay location	Tube			**VAC1**					**Final**	**Total**
**Humoral assays**												
HIV Screening ELISA	Local lab	Local lab	SST		5	—	—	—	—	—	—	**5**
HIV diagnostic ELISA	UW-VSL	UW-VSL	SST		—	—	—	—	5	5	5	**15**
HIV Neut. antibody (Ab)	CSR	CL-Duke	SST		—	5	—	5	—	—	5	**15**
HIV Binding ELISA	CSR	CL-Duke	SST		—	5	—	5	—	—	5	**15**
Ad5 Neut. Ab studies	VRC/CSR	VRC	SST		5	—	—	5	—	5	5	**20**
**Viral assays**												
HIV RNA PCR	UW-VSL	UW-VSL	EDTA		—	5	—	—	5	5	5	**20**
**Cellular assays**												
ELISpot/ICS	CSR	CL-FHCRC	Na Hep		—	80	—	80	—	—	80	**240**
ICS	CSR	VRC	Na Hep		—	40	40	40	—	40	40	**200**
HLA typing	CSR	CL-Duke	ACD		—	20	—	—	—	—	—	**20**
**Specimen storage**												
PBMC	CSR	CSR	Na Hep		—	—	—	60	150	110	150	**470**
Serum	CSR	CSR	SST		—	10	10	20	20	20	20	**100**
**Safety labs**												
CBC/differential/platelets	Local lab	Local lab	EDTA		5	—	5	5	5	5	5	**30**
Chemistry panel	Local lab	Local lab	SST		10	—	5	5	5	5	5	**35**
**Total**					**25**	**165**	**60**	**225**	**190**	**195**	**325**	**1185**
56-Day total					**25**	**190**	**250**	**475**	**415**	**195**	**325**	
**Urine collection**												
Urinalysis					X	—	X	—	—	X	—	
Pregnancy test					X	X	—	X	—	—	—	

CSR = Central Specimen Repository; CL = Central Laboratory

The HVTN Laboratory Program includes endpoint laboratories at UW-VSL, Duke, FHCRC, and SAIL-NICD. UW-VSL = University of Washington Virology Specialty Laboratory (Seattle, Washington, USA); Duke = Duke University Medical Center (Durham, North Carolina, USA); FHCRC/UW = Fred Hutchinson Cancer Research Center/University of Washington (Seattle, Washington, USA); SAIL-NICD = South African Immunology Laboratory–National Institute for Communicable Diseases (Johannesburg, South Africa)

Screening may occur over the course of several contacts/visits up to and including Day 0 prior to vaccination. Additional tests performed at the screening visit include syphilis, HepB and HepC tests using serum samples.

Diagnostic HIV Western blot will be performed at UW-VSL as indicated from samples sent for diagnostic ELISA per the HVTN algorithm for diagnosis of HIV infections

Local labs may assign appropriate alternative tube types for locally performed tests.

Humoral and cellular assays at Day 84, Day 168, and Day 364 may be performed if a positive response is observed in the same assay at 4 weeks post-vaccination.

HLA-typing will be performed only when positive responses to cellular assays are observed. These data will be used to guide immunological assays of future interest.

Serum for the screening of pre-existing adenovirus titers will be sent directly to the VRC.

A portion of serum for storage will be reserved for humoral studies at VRC.

Protocol Modification History

Protocol modifications are made to HVTN protocols via clarification memos, letters of amendment, or full protocol amendments. HVTN protocols are modified and distributed according to the standard HVTN procedures as described in the HVTN MOP (Organization and Policy>Vaccine Selection and Protocol Development).

The table below describes the version history of, and modifications to, Protocol HVTN 054.

Date	Protocol version	Protocol modification	Summary of modifications
13-Feb-06	Version 1	Clarification Memo 4	Item 1 Replaced: Viral and Rickettsial Disease Laboratory (Richmond, California) by University of Washington Virology Specialty Laboratory (Seattle, Washington))<br>Item 3 Changed: Tube type for HIV RNA PCR specimens from PPT to EDTA in Appendix B
12-Oct-05	Version 1	Clarification Memo 3	Item 1 Discontinued: Specimen collection for HIV DNA PCR testing
26-Aug-05	Version 1	Clarification Memo 2	Item 1 Permission for local labs to use appropriate alternative tube types for locally performed tests
10-Feb-05	Version 1	Clarification Memo 1	Item 1 Weekly safety reports are annotated as necessary<br>Item 2 Specimens for Ad5 neutralizing antibody studies at screening shipped directly to VRC<br>Item 3 Specimens for HLA typing sent to the central specimen repository<br>Item 4 Pregnancy tests performed on the day of vaccination prior to vaccination
06-Dec-04	Version 1	N/A	Original protocol
